# Primaquine and Chloroquine Fumardiamides as Promising Antiplasmodial Agents

**DOI:** 10.3390/molecules24152812

**Published:** 2019-08-01

**Authors:** Maja Beus, Diana Fontinha, Jana Held, Zrinka Rajić, Lidija Uzelac, Marijeta Kralj, Miguel Prudêncio, Branka Zorc

**Affiliations:** 1 University of Zagreb, Faculty of Pharmacy and Biochemistry, A. Kovačića 1, HR-10 000 Zagreb, Croatia; 2 Instituto de Medicina Molecular, Faculdade de Medicina, Universidade de Lisboa, Av. Prof. Egas Moniz, 1649-028 Lisboa, Portugal; 3 University of Tübingen, Institute of Tropical Medicine, Wilhelmstraße 27, 72074 Tübingen, Germany; 4 Rudjer Bošković Institute, Division of Molecular Medicine, Laboratory of Experimental Therapy, 10 000 Zagreb, Croatia

**Keywords:** primaquine, chloroquine, antiplasmodial activity, cytotoxicity, fumardiamide

## Abstract

This paper describes a continuation of our efforts in the pursuit of novel antiplasmodial agents with optimized properties. Following our previous discovery of biologically potent asymmetric primaquine (PQ) and halogenaniline fumardiamides (**1**–**6**), we now report their significant in vitro activity against the hepatic stages of *Plasmodium* parasites. Furthermore, we successfully prepared chloroquine (CQ) analogue derivatives (**11**–**16)** and evaluated their activity against both the hepatic and erythrocytic stages of *Plasmodium*. Our results have shown that PQ fumardiamides (**1**–**6)** exert both higher activity against *P. berghei* hepatic stages and lower toxicity against human hepatoma cells than the parent drug and CQ derivatives (**11**–**16)**. The favourable cytotoxicity profile of the most active compounds, **5** and **6**, was corroborated by assays performed on human cells (human breast adenocarcinoma (MCF-7) and non-tumour embryonic kidney cells (HEK293T)), even when glucose-6-phosphate dehydrogenase (G6PD) was inhibited. The activity of CQ fumardiamides on *P. falciparum* erythrocytic stages was higher than that of PQ derivatives, comparable to CQ against CQ-resistant strain *Pf*Dd2, but lower than CQ when tested on the CQ-sensitive strain *Pf*3D7. In addition, both sets of compounds showed favourable drug-like properties. Hence, quinoline fumardiamides could serve as a starting point towards the development of safer and more effective antiplasmodial agents.

## 1. Introduction

Despite enormous efforts made by the scientific community, malaria remains a deadly disease, especially among pregnant women and children. According to the 2018 World Malaria Report, malaria is responsible for 266,000 deaths of children aged under five years worldwide [1]. In order to achieve the 2020 milestones of the WHO Global Technical Strategy for Malaria 2016–2030, which include a global reduction of at least 40% in malaria incidence and mortality rates, and disease elimination in at least 10 countries, substantial amounts of funding and research are required [2].

Malaria is caused by *Plasmodium* parasites, which infect their mammalian host upon the bite of an infected female *Anopheles* mosquito. Injected sporozoites travel to the liver, where they undergo an asymptomatic but obligatory phase of intra-hepatic replication. The liver stage of infection ends with the release of thousands of merozoites into the blood, where they cyclically invade and replicate inside erythrocytes, causing the symptoms of malaria. Sexual forms of the parasites formed during this phase of infection can be ingested by a feeding mosquito, and subsequently undergo a developmental process that culminates in the formation of infective sporozoites, ready to start a new infection cycle [3].

Out of five *Plasmodium* species that cause malaria in humans, *P. vivax* and *P. ovale* can form hypnozoites in the liver, dormant forms of the parasite that can reactivate and cause clinical relapses of the disease, weeks or months after the primary infection [4,5]. Until July 2018, primaquine (PQ), an 8-aminoquinoline derivative, was the only drug effective against both the active hepatic stages of *Plasmodium* parasites, as well as *P. vivax* and *P. ovale* hypnozoites, registered worldwide. One of PQ’s main downsides is its low oral bioavailability, underlaid by its rapid metabolic degradation to inactive carboxyprimaquine [6,7]. As a consequence, PQ is administered once daily for two weeks, but this regimen is associated with poor compliance, resulting in a lower drug effectiveness [8]. Tafenoquine (TQ) is another 8-aminoquinoline derivative, recently approved for the *P. vivax* malaria therapy in combination with a drug acting on the erythrocytic stage of the infection [9,10,11] as well as for prophylaxis of *P. falciparum* malaria. Due to its extremely long elimination half-life (approximately 15 days), only a single dose of TQ is required for the treatment of hypnozoites, which ensures better adherence to the therapy than PQ [12]. However, both drugs induce hemolysis in patients with congenital glucose-6-phosphate dehydrogenase (G6PD) deficiency [10,11]. Thus, novel therapies that target the hepatic stages of *Plasmodium* parasites, including hypnozoites, while having reduced hemotoxic properties, are needed.

**Figure 1:**
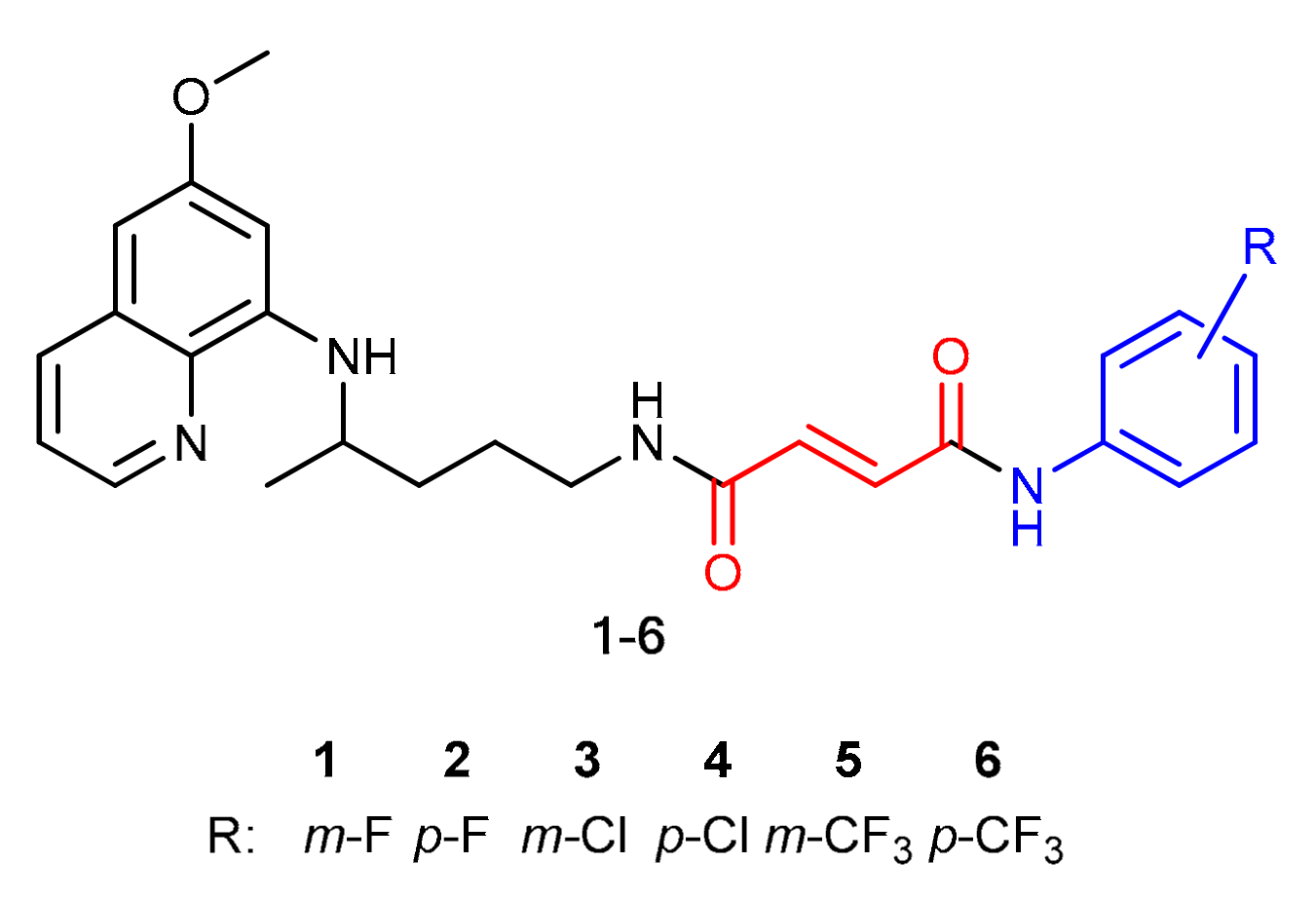
Hybrids **1**–**6** composed of PQ (black), fumaric acid (red) and halogenaniline (blue) fragments [20].

This paper represents a continuation of our work on the derivatization of antiplasmodial drugs, which included the successful development and evaluation of around 150 PQ derivatives [13,14,15,16,17,18,19]. Recently, we have prepared a series of hybrid molecules **1**–**6** composed of PQ, fumaric acid, and halogenaniline fragments (Figure 1), and we evaluated their antimicrobial activity against various microorganisms, their biofilm eradication ability, and their cytostatic activity [20]. The analogues without carbon-carbon double bonds, i.e., derivatives of succinic acid, were also prepared and tested. In all of the biological assays performed, the activity of fumardiamides was superior to succindiamides, indicating the importance of the double bond conjugated to the carbonyl group (Michael acceptor) in the molecules. Since the antiplasmodial potential of these compounds has not been evaluated until now, the objective of this work was to assess their antiplasmodial activity in vitro against the hepatic and erythrocytic stages of *Plasmodium*. In order to estimate the impact of the quinoline core on the biological activity, we prepared the analogous compounds with the chloroquine (CQ) pharmacophore. Here, we disclose their synthesis and the evaluation of their antiplasmodial activity.

## 2. Results and Discussion

### 2.1. Chemistry

**Scheme 1:**
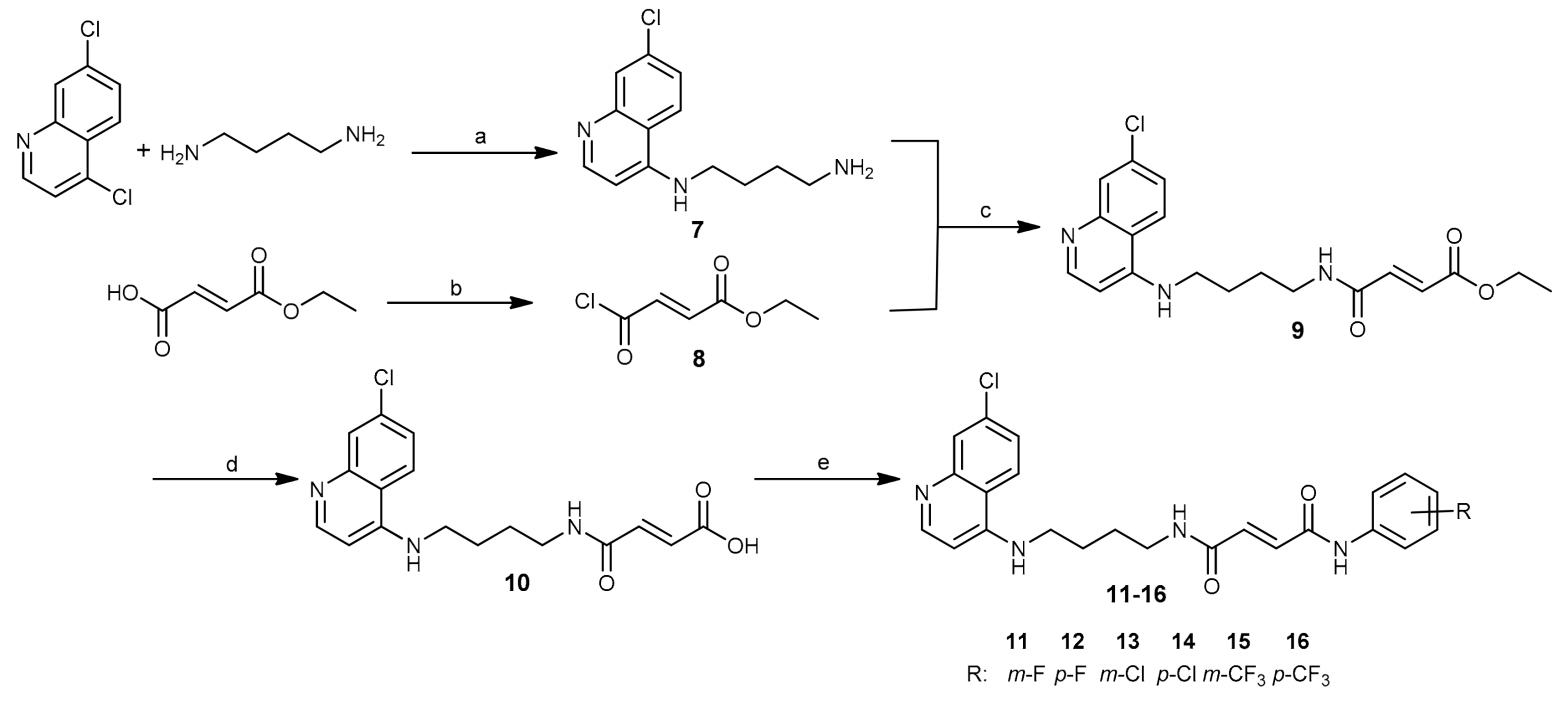
Synthesis of chloroquine derivatives **11**–**16**. Reagents and conditions: a) MW, 95 °C, 1 h; b) SOCl_2_, rt, 24 h; c) TEA, DCM, rt, 1 h; d) LiOH, MeOH, H_2_O, rt, 1 h; e) halogenaniline, HATU, DIEA, DMF, rt, 2–24 h or MW, 65 °C, 1 h.

Analogous to PQ-based hybrids **1**–**6**, in fumardiamides **11**–**16**, one amide bond was achieved with CQ, and the other one with halogenanilines (*m*- and *p*-fluoroaniline, *m*- and *p*-chloroaniline, *m*- and *p*-trifluoromethylaniline). A synthetic route developed earlier for the preparation of fumardiamides **1**–**6** was successfully applied here (Scheme 1). 

In short, the microwave-assisted method was applied for the first step to yield the primary amine **7** with the CQ core. Mono-ethyl fumarate was converted to the corresponding carboxylic acid chloride **8**, which reacted with the primary amino group of **7** to give the amidoester **9**. Basic hydrolysis of the product **9** by lithium hydroxide yielded the carboxylic acid **10**. In the last step, the coupling between the acid **10** and halogenanilines was accomplished by means of (1-[bis(dimethylamino)methylene]-1*H*-1,2,3-triazolo[4,5-*b*]pyridinium 3-oxid hexafluorophosphate (HATU) and *N,N*-diisopropylethylamine (DIEA). Purification of these compounds was carried out using crystallization methods and/or column chromatography. Yields for the first three reaction steps were high (73%−100%), but the yields varied from 25% to 91% for the last step. Taken together, our method offered title compounds **11**–**16** in good to moderate yields. All synthesized compounds were checked for purity and identified using elemental analysis, IR, ^1^H and ^13^C-NMR spectroscopy, and MS. The data obtained were in agreement with the proposed structures and are given in detail in the Supplementary Materials.

### 2.2. Antiplasmodial Activity

#### 2.2.1. Erythrocytic Stages

We conducted the in vitro screening of the activity of PQ (**1**–**6**) and CQ (**11**–**16**) fumardiamides against the erythrocytic stages of the CQ-sensitive (*Pf*3D7) and CQ-resistant (*Pf*Dd2) *P. falciparum* strains. Both PQ and CQ were included as positive controls, and DMSO served as a negative control in these assays. PQ derivatives were poorly active, as only compound **2** displayed an IC_50_ value against *Pf*3D7 below 10 µM (IC_50_ = 7.74 µM). Such findings were not surprising, as PQ itself possesses only modest erythrocytic stage activity [21]. Moreover, the basic aliphatic amine required for such a type of activity is masked through the amide bond with fumaric acid. In contrast, the *Pf*3D7 strain was susceptible to CQ hybrids **11**–**16** in nanomolar concentrations. However, the most active compounds **14** and **16** were still less active than CQ (IC_50_ = 0.035 µM vs. 0.0037 µM). On the other hand, their activity against the *Pf*Dd2 strain was similar to that of CQ (Table 1). It is worth noting that *m*-derivatives **11**, **13**, and **15** have consistently shown higher activity than their *p*-substituted counterparts.

#### 2.2.2. Hepatic Stages

**Figure 2:**
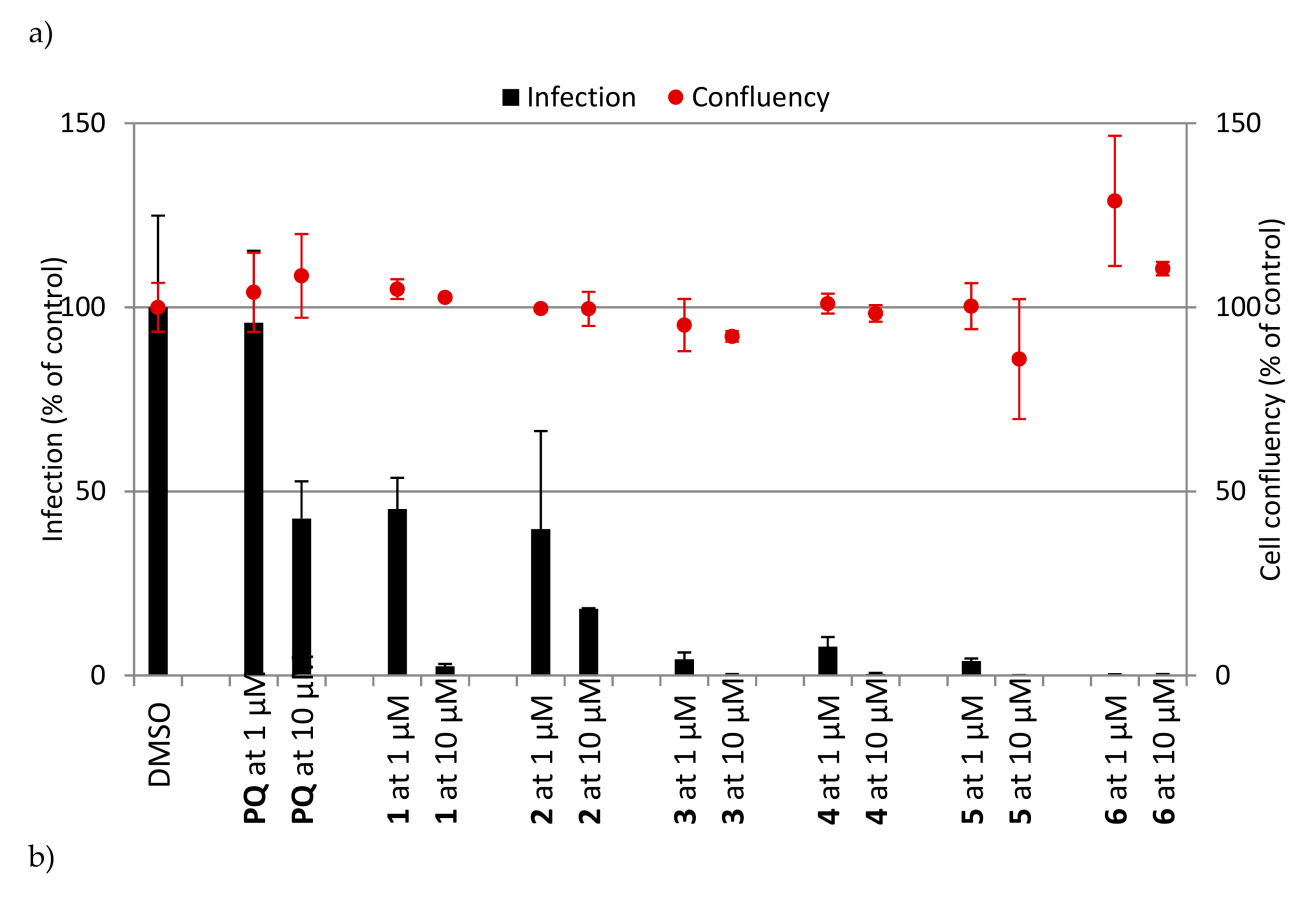

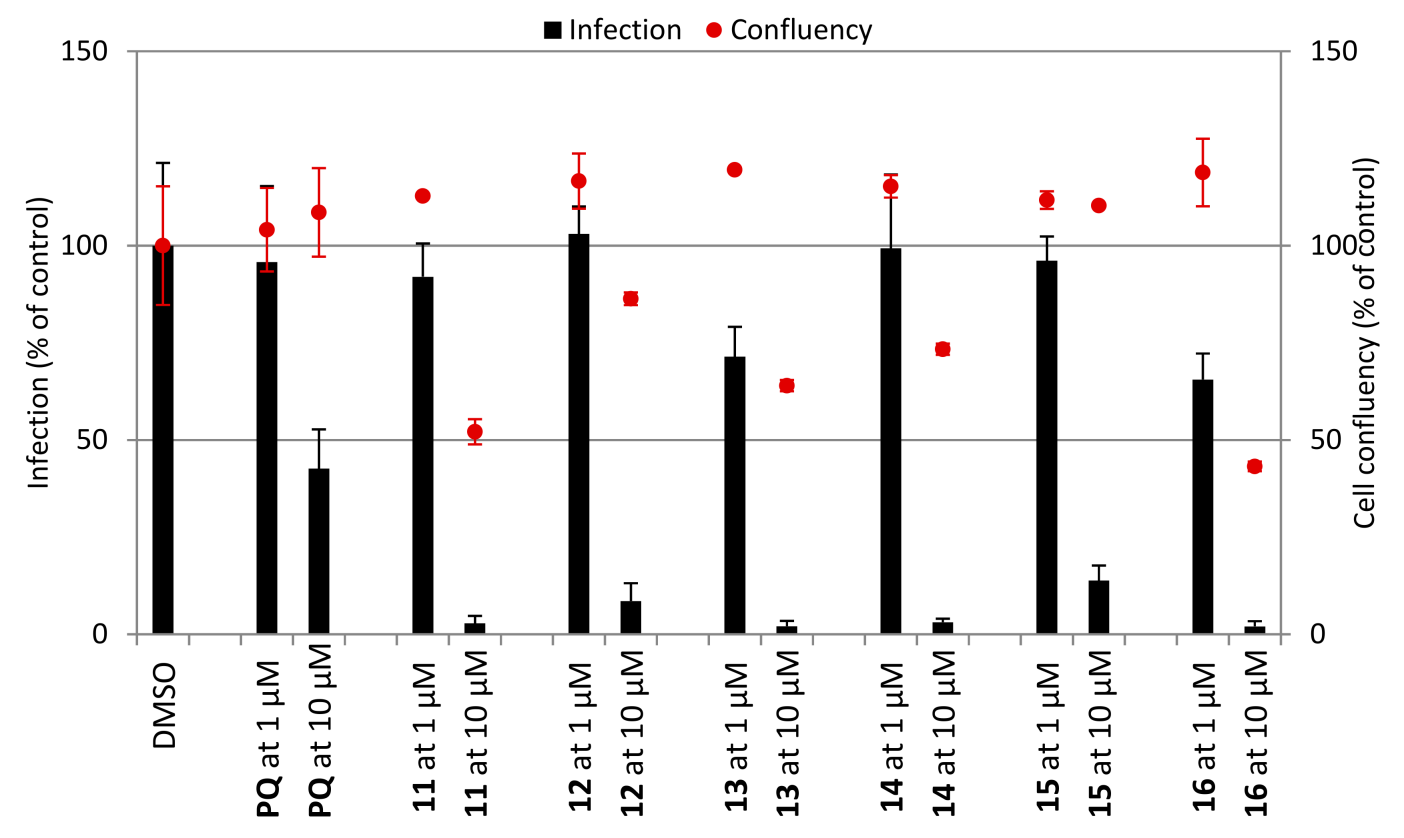
In vitro activity against *P. berghei* liver stages of (**a**) PQ derivatives **1**–**6** and (**b**) CQ derivatives **11**–**16** at 1 and 10 μM concentrations. Total parasite load (infection scale, bars) and cell viability (cell confluency scale, dots) are shown. Results were normalized to the negative control, dimethyl sulphoxide (DMSO), and are represented as mean ± SD, *n* = 1.

**Figure 3:**
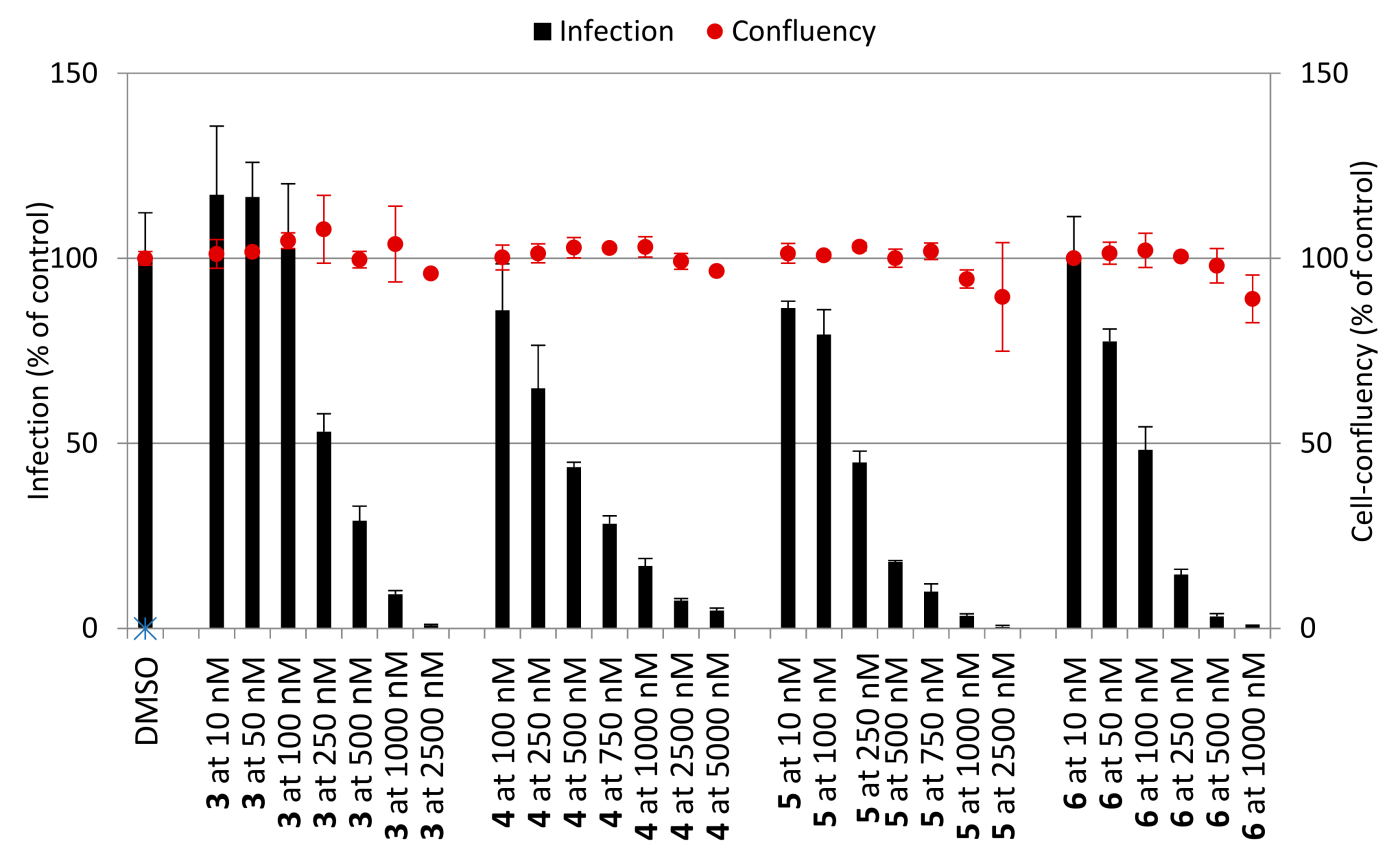
Dose-dependent response of the selected PQ derivatives against *P. berghei* hepatic stages. IC_50_ values are provided in Table 1.

Next, we examined the antiplasmodial activity of PQ (**1**–**6**) and CQ (**11**–**16**) derivatives against the hepatic stages of *P. berghei*, as well as toxicity to human hepatoma cells (Huh7) (Figure 2). PQ was included as a positive control, whereas DMSO served as a negative control in these assays. The results showed that PQ fumardiamides were markedly more active than the parent drug and their CQ counterparts. In addition, cell confluency measurements indicated that all PQ fumardiamides were non-toxic to human hepatoma cells. Such findings are in accordance with previous cytotoxicity studies of PQ fumardiamides on a panel of human cancer cell lines [20]. Conversely, CQ analogues displayed a higher level of toxicity than the PQ counterparts **1**–**6**. Additionally, IC_50_ values were determined for the most potent and promising PQ derivatives **3**–**6**. All four compounds exerted marked activities against hepatic parasites, with IC_50_ values ranging between 0.11 and 0.39 μM (Figure 3 and Table 1), which are at least an order of magnitude lower than the parent drug (IC_50_ = 8.4 ± 3.4 μM).

#### 2.2.3. Cytotoxicity Assay in Human Cell Lines

**Figure 4:**
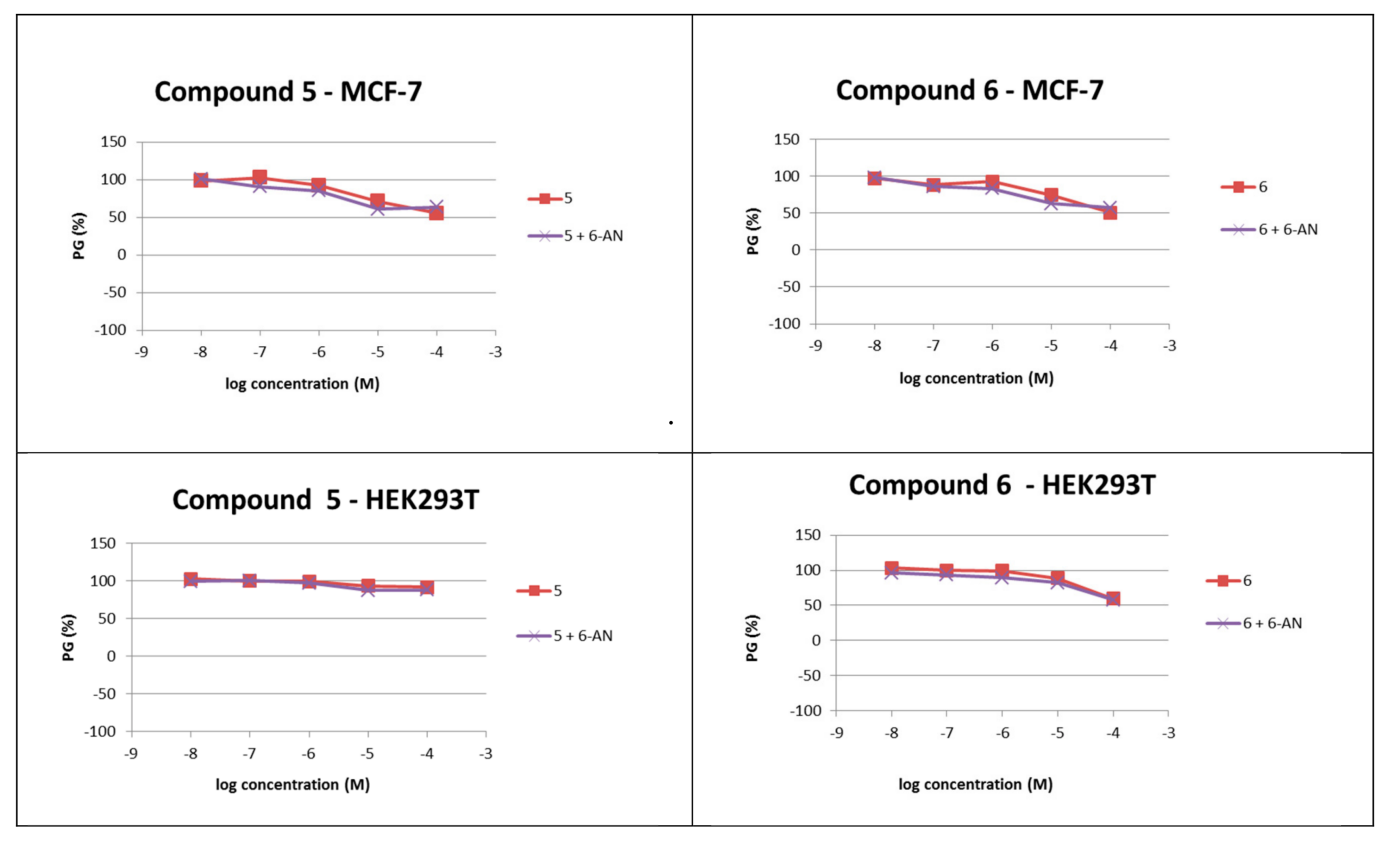
Dose-response profiles for compounds **5** and **6**, alone or in the combination with 6-aminonicotinamide, evaluated in vitro on MCF-7 and HEK293T cell lines.

Additional evaluation of the cytotoxicity of the most active PQ derivatives **5** and **6** was performed on two human cell lines from different tissues, MCF-7 (breast adenocarcinoma) and HEK293T (non-tumour embryonic kidney). Neither of the compounds displayed toxicity towards the tested cell lines (Table 2). Further, we tested whether the favourable cytotoxicity profile of PQ derivatives **5** and **6** was maintained in the glucose-6-phosphate dehydrogenase (G6PD)-deficient conditions, which were induced by the addition of the G6PD inhibitor 6-aminonicotinamide (6-AN) at a non-toxic concentration (*c* = 1 μM) to the cells [18]. No differences between 6-AN-treated and non-treated cell lines were observed in terms of growth inhibition (Table 2, Figure 4). Thus, these data suggest that derivatives **5** and **6** are promising lead compounds for the development of hepatic stage antiplasmodial compounds with an improved safety profile in G6PD-deficient patients as well.

#### 2.2.4. Evaluation of Drug-Like Properties

The evaluation of the PQ fumardiamides’ drug-likeness was reported earlier [20]. Thus, here we evaluated the drug-likeness of CQ derivatives **11**–**16**, by employing the Chemicalize.org software to calculate an array of physicochemical parameters, including the topological polar surface area (TPSA), the number of atoms, molecular weight (MW), the partition coefficient (log *P*), the H-bond donor (HBD), the H-bond acceptor (HBA), and molecular refractivity (MR) (Table 3) [22]. Our results show that all hybrids **11**–**16** fall within the Lipinski’s and Gelovani’s rules for prospective small molecular drugs (MW ≤ 500, log *P* ≤ 5, number of H-bond donors ≤ 5, number of H-bond acceptors ≤ 10, TPSA < 140 Å^2^, MR within the range of 40 and 130 cm^3^/mol, the number of atoms 20–70).

**Figure 5:**
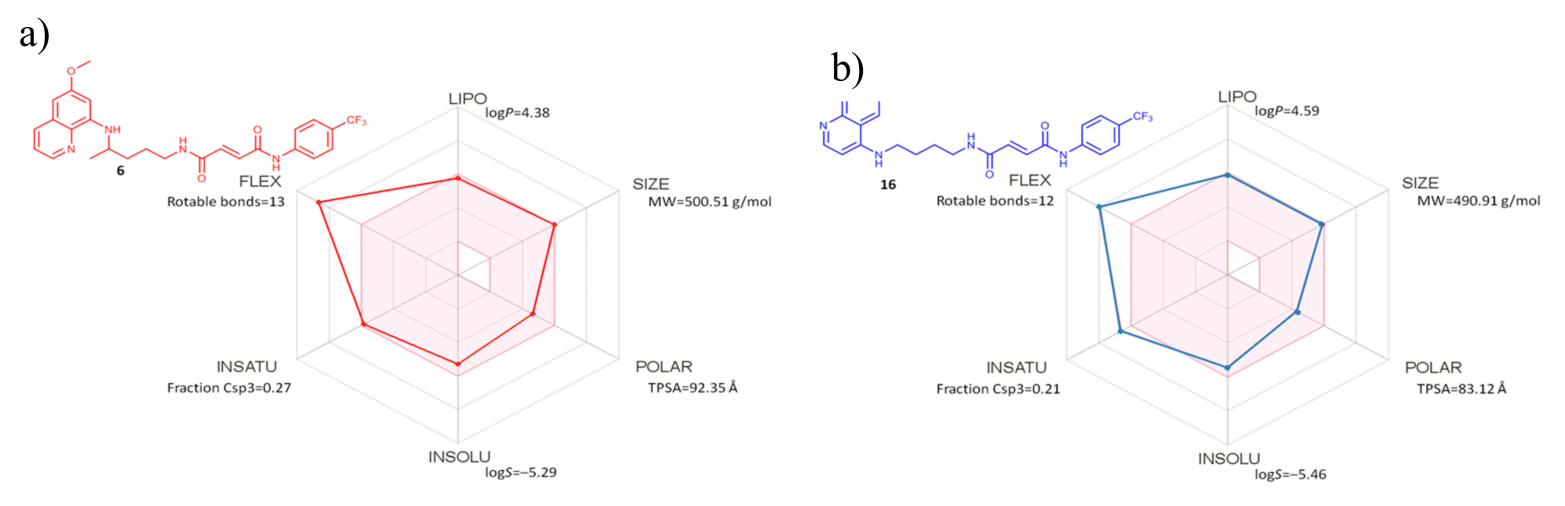
SwissADME bioavailability radars for the most active compounds: (**a**) PQ derivative **6** and (**b**) CQ derivative **16**. The pink area represents the optimal range for each property: lipophilicity (LIPO) (XLOGP3 between −0.7 and +5.0), molecular mass (SIZE) (between 150 and 500 g∙mol^–1^), polarity (POLAR) (TPSA between 20 and 130 Å^2^), solubility (INSOLU) (log *S* not higher than 6), saturation (INSATU) (fraction of carbons in the sp^3^ hybridization not less than 0.25), and flexibility (FLEX) (no more than nine rotatable bonds).

Both PQ and CQ hybrids were further analyzed through available filters for pan assay interference compounds (PAINS), which identify a substructure with an ability to interfere in any biological assay, based on various mechanisms [23]. Three computer programs, ZINC (http://zinc15.docking.org/PAINS/), PAINS Remover (http://www.cbligand.org/PAINS/), and SwissADME (http://www.swissadme.ch/), were employed for compounds **1**–**6** and **11**–**16**. In addition, we carried out the in silico identification of potential aggregators (Aggregator Advisor, http://advisor.docking.org). All tested compounds returned no PAINS and no aggregator alerts, thus showing that the selected hybrid compounds passed all filters. SwissADME bioavailability radars [24] of the most active compounds of both series, namely compounds **6** and **16**, showed that all analyzed parameters are in the optimal range, with the exception of flexibility, which falls out of the set borders (Figure 5).

## 3. Materials and Methods

### 3.1. Chemistry

#### 3.1.1. Materials and Methods

Melting points were determined on the SMP3 apparatus (Barloworld Scientific, Stone, UK) in open capillaries and are presented uncorrected. A CEM Discover microwave reactor was used for microwave reactions (CEM GmbH, Kamp-Lintfort, Germany). IR spectra were recorded on Spectrum One (Perkin-Elmer, Waltham, MS, USA) and UV-Vis spectra on Lambda 20 double beam spectrophotometers (Perkin-Elmer, UK). NMR ^1^H and ^13^C spectra were recorded at 25 °C on the NMR Avance 600 spectrometer (Bruker, Leipzig, Germany) at 300.13 or 600.13 and 75.47 or 150.9 MHz for ^1^H and ^13^C nuclei, respectively. Chemical shifts (*δ*) are reported in parts per million (ppm) relative to tetramethylsilane in the ^1^H and the dimethyl sulfoxide (DMSO) residual peak as a reference in the ^13^C spectra (39.51 ppm). Coupling constants (*J*) are reported in hertz (Hz). Mass spectra were collected on an HPLC-MS/MS instrument (HPLC, Agilent Technologies 1200 Series; MS, Agilent Technologies 6410 Triple Quad, Santa Clara, CA, USA) using electrospray ionization in the positive mode. Elemental analyses were performed on a CHNS LECO analyzer (LECO Corporation, St. Joseph, MI, USA). All compounds were routinely checked by TLC with Merck silica gel 60F-254 glass plates using dichloromethane/methanol 9.5:0.5, 9:1, 8.5:1.5, and cyclohexane/ethyl acetate/methanol 1:1:0.5 as the solvent systems. Spots were visualized by short-wave UV light and iodine vapour. Column chromatography was performed on silica gel at 0.063 to 0.200 mm (Sigma-Aldrich, St. Louis, Missouri, United States, USA) with the same eluents used in TLC.

All chemicals and solvents were of analytical grade and purchased from commercial sources. 4,7-Dichloroquinoline, 4-chloro-2,8-bis(trifluoromethyl)quinoline, butane-1,4-diamine, (*E*)-4-ethoxy-4-oxobut-2-enoic acid (mono-ethyl fumarate), 3-fluoroaniline, 4-fluoroaniline, 3-chloroaniline, 4-chloroaniline, 3-trifluoromethylaniline, 4-trifluoromethylaniline, triethylamine (TEA), DIEA, and HATU were purchased from Sigma-Aldrich. Anhydrous solvents were dried and redistilled prior to use.

#### 3.1.2. Procedures for the Synthesis of Compounds **7**–**10**.

*N^1^-(7-chloroquinolin-4-yl)butane-1,4-diaminediamine (***7***).* A mixture of 4,7-dichloroquinoline (0.599 g, 0.002 mol) and 1,4-diaminobutane (1.763 g, 0.02 mol) was stirred under microwave irradiation (300 W) at 95 °C. After 60 min, the reaction mixture was diluted with dichloromethane and extracted with 5% NaOH (4 × 40 mL) and washed with water (2 × 40 mL). The organic layer was dried over anhydrous sodium sulfate, filtrated, and evaporated under reduced pressure. The crude product **7** (0.499 g, 100%) was used in the further reaction without purification.

*(E)-ethyl 4-chloro-4-oxobut-2-enoate (Mono-ethyl fumarate chloride) (***8***).* A solution of 0.288 g mono-ethyl fumarate (2 mmol) in 7 mL thionyl chloride was kept overnight and evaporated under reduced pressure. The residue was trice triturated with dichloromethane and the solvent was evaporated again. Freshly prepared crude product **8** (0.315 g, 97%) was used in the further reaction without purification.

*Ethyl (E)-4-((4-((7-chloroquinolin-4-yl)amino)butyl)amino)-4-oxobut-2-enoate (***9***).* A solution of compound **7** (0.375 g, 1.5 mmol) and TEA (0.152 g, 1.5 mmol) in 10 mL of dichloromethane was added dropwise to a solution of mono-ethyl fumarate chloride **8** (0.288 g, 1.5 mmol) in 10 mL dichloromethane. The reaction mixture was stirred for 1 h at room temperature and extracted 3 times with brine. The organic layer was dried over sodium sulfate, filtered, and evaporated under reduced pressure. After purification by column chromatography (mobile phase dichloromethane/methanol 8.5:1.5) and crystallization from ether, 0.412 g (73%) of pale yellow solid **9** was obtained; mp 207–209 °C; IR (KBr): *ν*_max_ 2566, 3368, 3282, 3082, 2946, 2872, 2796, 1722, 1672, 1643, 1584, 1454, 1368, 1334, 1302, 1238, 1176, 1080, 1022, 974, 896, 850, 806, 766, 692, 666, 642, 570, 540 cm^–1^; ^1^H-NMR (DMSO-*d*_6_) *δ* 8.56 (t, 1H, *J* = 5.5 Hz), 8.39 (d, 1H, *J* = 5.5 Hz), 8.29 (d, 1H, *J* = 9.1 Hz), 7.79 (d, 1H, *J* = 2.2 Hz), 7.49–7.39 (m, 2H,), 7.00 (d, 1H, *J* = 15.5 Hz), 6.56 (d, 1H, *J* = 15.5 Hz), 6.50 (d, 1H, *J* = 5.6 Hz), 4.18 (q, 2H, *J* = 7.1 Hz), 3.35-3.16 (m, 4H), 1.75–1.50 (m, 4H), 1.24 (t, 3H, *J* = 7.1 Hz); ^13^C-NMR (DMSO-*d*_6_) *δ* 165.08, 162.73, 151.41, 150.35, 148.51, 137.61, 133.61, 128.18, 127.02, 124.19, 124.14, 117.34, 98.67, 60.67, 42.04, 38.55, 26.48, 25.18, 14.01; ESI-MS: *m*/*z* calculated for C_19_H_22_ClN_3_O_3_: 375.13, found: 376.1 (M + 1)^+^; Anal. Calcd. for C_19_H_22_ClN_3_O_3_: C, 60.72; H, 5.90; N, 11.18. Found: C, 60.58; H, 5.51; N, 11.38.

*(E)-4-((4-((7-chloroquinolin-4-yl)amino)butyl)amino)-4-oxobut-2-enoic acid (***10***)*. A solution of 0.126 g (3 mmol) lithium hydroxide monohydrate in 10 mL water was added to a solution of 0.225 g (0.6 mmol) ester **9** in 10 mL methanol. The reaction mixture was stirred for 3 h at room temperature. Methanol was evaporated under reduced pressure and the aqueous residue was acidified with 10%-HCl to pH 1. The precipitated product was filtered and washed with water until neutral. 0.190 g (97%) of pale yellow solid **10** was obtained; mp 227–228 °C; IR (KBr): *ν*_max_ 3280, 3070, 3940, 2872, 2364, 2060, 1956, 1620, 1558, 1456, 1366, 1206, 1136, 1090, 984, 900, 816, 762, 658, 584 cm^–1^; ^1^H-NMR (DMSO-*d*_6_) *δ* 8.47 (t, 1H, *J* = 5.5 Hz), 8.38 (d, 1H, *J* = 5.5 Hz), 8.27 (d, 1H, *J* = 9.1 Hz), 7.77 (d, 1H, *J* = 2.2 Hz), 7.52–7.33 (m, 2H), 6.88 (d, 1H, *J* = 15.5 Hz), 6.55–6.45 (m, 2H), 3.35–3.09 (m, 4H), 1.77–1.48 (m, 4H); ^13^C-NMR (DMSO-*d*_6_) *δ* 166.71, 163.21, 151.50, 150.32, 148.59, 136.60, 133.57, 130.12, 127.09, 124.18, 124.13, 117.36, 98.67, 42.05, 38.50, 26.55, 25.21; ESI-MS: *m*/*z* calculated for C_17_H_18_ClN_3_O_3_: 347.10, found: 348.0 (M + 1)^+^; Anal. Calcd. C_17_H_18_ClN_3_O_3_: C, 58.71; H, 5.22; N, 12.08. Found: C, 59.12; H, 5.12; N, 11.97.

#### 3.1.3. General Procedure for the Synthesis of Fumardiamides **11**–**16**

A solution of 0.094 g (0.27 mmol) compound **10**, 0.068 g (0.54 mmol) DIEA, and 0.103 g (0.27 mmol) HATU in 1 mL of *N*,*N*-dimethylformamide was stirred at room temperature. After 10 min, 0.297 mmol of the corresponding halogenaniline was added. The reaction mixture was stirred for 2 to 24 h at room temperature or for 2 h under microwave irradiation (300 W) at 65 °C. The solvent was evaporated under reduced pressure.

*(2E)-N’-{4-[(7-chloroquinolin-4-yl)amino]butyl}-N-(3-fluorophenyl)but-2-enediamide (***11***).* Reaction conditions: 2 h, room temperature. From the reaction of 0.094 g acid **10** and 0.033 g (0.297 mmol) 3-fluoroaniline and after purification by column chromatography (mobile phase dichloromethane/methanol 8.5:1.5) and crystallization from ether, 0.030 g (25%) of white solid **11** was obtained; mp 229–232 °C; IR (KBr): *ν*_max_ 3634, 3282, 3092, 2938, 3868, 1650, 1606, 1558, 1490, 1428, 1384, 1338, 1262, 1212, 1186, 1136, 1080, 1042, 1020, 970, 938, 902, 854, 810, 772, 716, 678, 594, 520 cm^–1^; ^1^H-NMR (DMSO-*d*_6_) *δ* 10.77 (s, 1H), 8.68–8.40 (m, 4H), 7.92 (d, 1H, *J* = 1.4 Hz), 7.70 (d, 1H, *J* = 11.6 Hz), 7.62 (dd, 1H, *J* = 9.0, 1.7 Hz), 7.38 (m, 2H′), 7.03 (q, 2H, *J* = 15.1 Hz), 6.93 (t, 1H, *J* = 8.49 Hz), 6.72 (d, 1H, *J* = 6.4 Hz), 3.48–3.40 (m, 2H), 3.29–3.21 (m, 2H), 1.76–1.66 (m, 2H), 1.64–1.55 (m, 2H); ^13^C-NMR (DMSO-*d*_6_) *δ* 163.69–160.49 (d, *J* = 242.80 Hz), 163.39, 162.62, 154.77, 143.78, 140.61-140.46 (d, *J* = 11.38 Hz), 139.69, 137.42, 134.32, 132.39, 130.54–130.42 (d, *J* = 9.34 Hz), 126.47, 125.60, 119.96, 115.65, 115.18, 110.42–110.14 (d, *J* = 20.55), 106.33–105.98 (d, *J* = 20.55 Hz), 98.61, 42.73, 38.37, 26.34, 25.04; ESI-MS: *m*/*z* calculated for C_23_H_22_ClFN_4_O_2_: 440.14, found: 441.0 (M + 1)^+^; Anal. Calcd. C_23_H_22_ClFN_4_O_2_: C, 62.66; H, 5.03; N, 12.71. Found: C, 62.70; H, 5.15; N, 12.79.

*(2E)-N’-{4-[(7-chloroquinolin-4-yl)amino]butyl}-N-(4-fluorophenyl)but-2-enediamide (***12***).* Reaction conditions: 2 h, room temperature. From the reaction of 0.094 g acid **10** and 0.033 g (0.297 mmol) 4-fluoroaniline and after extraction with ethyl acetate and crystallization from ether, 0.108 g (91%) of white solid **12** was obtained; mp 239 °C (decomp.); IR (KBr): *ν*_max_ 3284, 3072, 2956, 2932, 2864, 1636, 1582, 1542, 1510, 1452, 1406, 1368, 1334, 1278, 1212, 1172, 1140, 1080, 996, 934, 902, 834, 764, 690, 552, 516 cm^–1^; ^1^H-NMR (DMSO-*d*_6_) *δ* 10.58 (s, 1H), 8.55 (t, 1H, *J* = 37 Hz), 8.41 (d, 1H, *J* = 5.6 Hz), 8.34 (d, 1H, *J* = 9.1 Hz), 7.80 (d, 1H, *J* = 2.1 Hz), 7.72 (dd, 2H, *J* = 9.0, 5.0 Hz), 7.59 (t, 1H, *J* = 5.47 Hz), 7.47 (dd, 1H, *J* = 9.0, 2.2 Hz), 7.17 (t, 2H, *J* = 9.13 Hz), 7.02 (q, 2H, *J* = 15.1 Hz), 6.53 (d, 1H, *J* = 5.7 Hz), 3.40–3.19 (m, 4H), 1.75–1.52 (m, 4H); ^13^C-NMR (DMSO-*d*_6_) *δ* 163.44, 162.23, 159.85-156.66 (d, *J* = 241.00 Hz), 150.96, 150.62, 148.03, 135.30, 133.97, 133.79, 132.60, 126.59, 124.35, 124.23, 121.14–121.03 (d, *J* = 7.71 Hz), 117.26, 115.56–115.27 (d, *J* = 22.77 Hz), 98.64, 42.09, 38.49, 26.56, 25.20; ESI-MS: *m*/*z* calculated for C_23_H_22_ClFN_4_O_2_: 440.14, found: 441.0 (M + 1)^+^; Anal. Calcd. C_23_H_22_ClFN_4_O_2_: C, 62.66; H, 5.03; N, 12.71. Found: C, 62.45; H, 5.25; N, 12.53.

*(2E)-N-(3-chlorophenyl)-N’-{4-[(7-chloroquinolin-4-yl)amino]butyl}but-2-enediamide (***13***).* Reaction conditions: 16 h, room temperature. From the reaction of 0.094 g acid **10** and 0.038 g (0.297 mmol) 3-chloroaniline and after purification by column chromatography (mobile phase dichloromethane/methanol 8.5:1.5) and crystallization from ether, followed by crystallization from methanol, 0.032 g (26%) of white solid **13** was obtained; mp 232 °C (decomp.); IR (KBr): *ν*_max_ 3340, 3298, 3182, 3092, 3032, 2944, 2866, 2830, 2360, 1650, 1592 1542, 1482, 1426, 1334, 1234, 1214, 1170, 1142, 1078, 1034, 970, 900, 848, 808, 782, 678, 626, 588, 558 cm^–1^; ^1^H-NMR (DMSO-*d*_6_) *δ* 10.67 (s, 1H), 8.56 (t, 1H, *J* = 5.5 Hz), 8.43 (d, 1H, *J* = 5.9 Hz), 8.38 (d, 1H, *J* = 9.1 Hz), 8.01–7.87 (m, 2H), 7.83 (d, 1H, *J* = 2.1 Hz), 7.66–7.46 (m, 2H), 7.37 (t, 1H, *J* = 8.1 Hz), 7.15 (dd, 1H, *J* = 7.9, 1.1 Hz), 7.02 (q, 2H, *J* = 14.5 Hz), 6.60 (d, 1H, *J* = 6.0 Hz), 3.42–3.32 (m, 2H), 3.29–3.19 (m, 2H), 1.76–1.53 (m, 4H); ^13^C-NMR (DMSO-*d*_6_) *δ* 163.34, 162.64, 151.57, 149.35, 146.10, 140.25, 134.66, 134.42, 133.14, 132.33, 130.56, 125.10, 124.79, 124.56, 123.53, 118.79, 117.78, 116.88, 98.66, 42.26, 38.50, 26.52, 25.18; ESI-MS: *m*/*z* calculated for C_23_H_22_Cl_2_N_4_O_2_: 456.11, found: 457.0 (M + 1)^+^; Anal. Calcd. C_23_H_22_Cl_2_N_4_O_2_: C, 60.40; H, 4.85; N, 12.25. Found: C, 60.26; H, 4.62; N, 12.27.

*(2E)-N-(4-chlorophenyl)-N’-{4-[(7-chloroquinolin-4-yl)amino]butyl}but-2-enediamide (***14***).* Reaction conditions: 1 h, 65 °C, MW. From the reaction of 0.094 g acid **10** and 0.038 g (0.297 mmol) 4-chloroaniline and after crystallization from methanol, 0.088 g (71%) of white solid **14** was obtained; mp 237 °C (decomp.); IR (KBr): *ν*_max_ 3412, 3286, 3186, 3098, 3072, 2946, 2878, 2706, 2364, 1363, 1544, 1492, 1452, 1398, 1342, 1240, 1212, 1172, 1128, 1094, 1014, 986, 842, 76, 680, 584, 558, 506 cm^–1^; ^1^H-NMR (DMSO-*d*_6_) *δ* 10.71 (s, 1H), 9.51 (t, 1H, *J* = 5.2 Hz), 8.66 (d, 1H, *J* = 9.2 Hz), 8.60 (t, 1H, *J* = 5.6 Hz), 8.55 (d, 1H, *J* = 7.1 Hz), 8.04 (d, 1H, *J* = 2.0 Hz), 7.75 (dd, 3H, *J* = 12.8, 5.5 Hz), 7.39 (d, 2H, *J* = 8.9 Hz), 7.02 (q, 2H, *J* = 15.1 Hz), 6.89 (d, 1H, *J* = 7.2 Hz), 3.6–3.5 (m, 2H), 3.27–3.14 (m, 2H), 1.79–1.66 (m, 2H), 1.65–1.53 (m, 2H); ^13^C-NMR (DMSO-*d*_6_) *δ* 163.43, 162.42, 155.17, 143.06, 138.87, 137.81, 134.13, 132.50, 128.73, 127.35, 126.68, 125.74, 120.87, 119.30, 115.50, 98.59, 42.79, 38.34, 26.32, 25.01; ESI-MS: *m*/*z* calculated for C_23_H_22_Cl_2_N_4_O_2_: 456.11, found: 457.0 (M + 1)^+^; Anal. Calcd. C_23_H_22_Cl_2_N_4_O_2_: C, 60.40; H, 4.85; N, 12.25. Found: C, 60.34; H, 4.97; N, 12.13.

*(2E)-N’-{4-[(7-chloroquinolin-4-yl)amino]butyl}-N-[3-(trifluoromethyl)phenyl]but-2-enediamide (***15***).* Reaction conditions: 24 h, room temperature. From the reaction of 0.094 g acid **10** and 0.048 g (0.297 mmol) 3-trifluoroaniline and after purification by column chromatography (mobile phase dichloromethane/methanol 8.5:1.5) after crystallization from ether, 0.034 g (26%) of white solid **15** was obtained; mp 240 °C (decomp.); IR (KBr): *ν*_max_ 3418, 3210, 3072, 3002, 2958, 1620, 1550, 1490, 1414, 1312, 1182, 1136, 1068, 1022, 840, 656, 558 cm^–1^; ^1^H-NMR (DMSO-*d*_6_) *δ* 10.81 (s, 1H), 9.26 (t, 1H, *J* = 5.2 Hz), 8.62–8.53 (m, 4H), 7.94 (d, 1H, *J* = 2.1 Hz), 7.86 (d, 1H, *J* = 8.4 Hz, 1H), 7.81 (dd, 1H, *J* = 9.1, 2.1 Hz), 7.62 (t, 1H, *J* = 8.0 Hz), 7.49 (d, 1H, *J* = 7.8 Hz), 7.04 (q, 2H, *J* = 14.88 Hz), 6.93 (d, 1H, *J* = 7.1 Hz), 3.61–3.54 (m, 2H), 3.32–3.25 (m, 2H), 1.81–1.70 (m, 2H), 1.68–1.58 (m, 2H); ^13^C-NMR (DMSO-*d*_6_) *δ* 163.31, 162.78, 155.08, 143.56, 139.54, 139.14, 137.77, 134.47, 132.27, 130.17, 129.64-129.38 (q, *J* = 26.85 Hz), 126.75, 125.45, 122.93, 122.71-120.00 (q, *J* = 274.54 Hz), 120.22, 119.67, 115.53, 115.36, 98.68, 42.85, 38.41, 26.34, 25.06; ESI-MS: *m*/*z* calculated for C_24_H_22_ClF_3_N_4_O_2_: 490.14, found: 491.1 (M + 1)^+^; Anal. Calcd. C_24_H_22_ClF_3_N_4_O_2_: C, 58.72; H, 4.52; N, 11.41. Found: C, 58.90; H, 4.55; N, 11.58.

*(2E)-N’-{4-[(7-chloroquinolin-4-yl)amino]butyl}-N-[4-(trifluoromethyl)phenyl]but-2-enediamide (***16***).* Reaction conditions: 24 h, room temperature. From the reaction of 0.094 g acid **10** and 0.048 g (0.297 mmol) 4-trifluoroaniline and after crystallization from water, followed by crystallization from ether, 0.049 g (37%) of white solid **16** was obtained; mp 270 °C (decomp.); IR (KBr): *ν*_max_ 3254, 2972, 2914, 2836, 2760, 2722, 2546, 2488, 2426, 2362, 2098, 1976, 1712, 1610, 1590, 1544, 1472, 1442, 1398, 1324, 1210, 1154, 1104, 1066, 1018, 978, 956, 882, 842, 802, 768, 686, 650, 592, 508 cm^–1^; ^1^H-NMR (DMSO-*d*_6_) *δ* 10.92 (s, 1H), 9.30 (t, 1H, *J* = 2.54 Hz), 8.71–8.57 (m, 2H), 8.54 (d, 1H, *J* = 6.9 Hz), 8.01 (d, 1H, *J* = 2.0 Hz), 7.91 (d, 2H, *J* = 8.5 Hz), 7.73 (dd, 3H, *J* = 14.2, 5.2 Hz), 7.06 (q, 2H, *J* = 15.1 Hz), 6.86 (d, 1H, *J* = 7.0 Hz), 3.57–3.49 (m, 2H), 3.29–3.21 (m, 2H), 1.77–1.67 (m, 2H), 1.64–1.54 (m, 2H); ^13^C-NMR (DMSO-*d*_6_) *δ* 163.35, 162.88, 155.08, 143.26, 142.39, 139.08, 137.70, 134.62, 132.29, 126.65, 126.17, 125.70, 123.95–123.52 (q, *J* = 25.48 Hz), 122.53–118.93 (q, *J* = 270.75 Hz), 119.48–119.33 (q, *J* = 10.26), 115.54, 98.61, 42.79, 38.38, 26.33, 25.03; ESI-MS: *m*/*z* calculated for C_24_H_22_ClF_3_N_4_O_2_: 490.14, found: 491.1 (M + 1)^+^; Anal. Calcd. C_24_H_22_ClF_3_N_4_O_2_: C, 58.72; H, 4.52; N, 11.41. Found: C, 58.96; H, 4.73; N, 11.67.

### 3.2. In Vitro Drug Sensitivity Assay Against Erythrocytic Stages of P. falciparum

Antiplasmodial activity of fumardiamides **1**–**6** and **11**–**16** was tested in a drug sensitivity assay against two laboratory *P. falciparum* strains (3D7—CQ-sensitive, and Dd2—CQ-resistant), as previously described, using the histidine-rich protein 2 (HRP2) assay [25,26]. In brief, 96-well plates were pre-coated with the tested compounds in a three-fold dilution before ring stage parasites were added in complete culture medium at a hematocrit of 1.5% and a parasitaemia of 0.05%. After three days of incubation at 37 °C, 5% CO_2_, and 5% oxygen, plates were frozen until analyzed by HRP2-ELISA. All compounds were evaluated in duplicate in at least two independent experiments. The IC_50_ was determined by analysing the nonlinear regression of log concentration-response curves using the drc-package v0.9.0 of R v2.6.1 [27].

### 3.3. In Vitro Activity Against P. berghei Hepatic Stages

In vitro activity of the tested compounds against the liver stage of *P. berghei* infection was assessed as previously described [28,29]. Briefly, Huh7 cells, a human hepatoma cell line, were routinely cultured in 1640 Roswell Park Memorial Institute (RPMI) medium supplemented with 10% (v/v) fetal bovine serum, 1% (v/v) glutamine, 1% (v/v) penicillin/streptomycin, 1% non-essential amino acids, and 10 mM 4-(2-hydroxyethyl)-1-piperazineethanesulfonic acid (HEPES). For drug screening experiments, Huh7 cells were seeded at 1 × 10^4^ cell/well of a 96-well plate and incubated overnight at 37 °C with 5% CO_2_. Next, 10 mM stock solutions of test compounds were prepared in DMSO and were serially diluted in infection medium, i.e., culture medium supplemented with gentamicin (50 μg/mL) and amphotericin B (0.8 μg/mL), in order to obtain the test concentrations. On the day of the infection, the culture medium was replaced with serial dilutions of the test compounds and incubated for 1 h at 37 °C with 5% CO_2_. Next, 1 × 10^4^ firefly luciferase-expressing *P. berghei* sporozoites, freshly isolated from the salivary glands of female infected *Anopheles stephensi* mosquitoes, were added to the cultures, plates were centrifuged at 1800× *g* for 5 min at room temperature, and incubated at 37 °C with 5% CO_2_.

To assess the effect of each compound concentration in cell viability, at 46 hours post-infection (hpi), cultures were incubated with Alamar Blue (Thermo Fisher Scientific, Waltham, MA, USA), according to the manufacturer’s recommendations. Parasite load was then assessed by the bioluminescence assay (Biotium, Fremont, CA, USA), using the multi-plate reader Infinite M200 (Tecan, Männedorf, Switzerland). Nonlinear regression analysis was employed to fit the normalized results of the dose–response curves, and IC_50_ values were determined using GraphPad Prism 6.0 (GraphPad Software, La Jolla California USA).

### 3.4. Cytotoxicity Assay in Human Cell Lines

The experiments were carried out on 2 human cell lines: MCF-7 (breast carcinoma) and HEK293T (embryonic kidney). MCF-7 and HEK293T cells were cultured as monolayers and maintained in Dulbecco’s modified Eagle medium (DMEM), supplemented with 10% fetal bovine serum (FBS), 2 mM L-glutamine, 100 U/mL penicillin, and 100 μg/mL streptomycin in a humidified atmosphere with 5% CO_2_ at 37 °C. The panel cell lines were inoculated in parallel onto a series of standard 96-well microtiter plates on day 0, at 1 × 10^4^ to 3 × 10^4^ cells/mL, depending on the doubling time of a specific cell line. Test compounds were then added in five 10-fold dilutions (10^−8^ to 10^−4^ M) alone or in combination with 6-AN (*c* = 1 µM) and incubated for a further 72 hours. Working dilutions were freshly prepared on the day of the testing. 

After 72 hours of incubation, the cell growth rate was evaluated by performing the MTT assay [18]. The absorbance was directly proportional to cell viability. Each test point was performed in quadruplicate in three individual experiments. The IC_50_ values were calculated from the dose–response curves using linear regression analysis. Each result is a mean value from at least two separate experiments.

## 4. Conclusions

The antiplasmodial potential of previously prepared PQ fumardiamides **1**–**6** and novel CQ analogues **11**–**16** was evaluated against the hepatic and erythrocytic stages of *Plasmodium* parasites. The evaluation of the compounds’ activity against *P. berghei* hepatic stages in vitro revealed that: i) PQ hybrids show markedly higher activities than PQ itself, ii) PQ derivatives display higher activity than CQ fumardiamides, and iii) PQ fumardiamides are non-toxic to human hepatoma cells, whereas their CQ analogues exert higher levels of toxicity. The most active PQ derivatives **5** and **6** showed low cytotoxicity towards human cells (MCF-7 and HEK293T), which was also founnd when G6PD was inhibited. CQ fumardiamides displayed higher activity than PQ derivatives against *P. falciparum* erythrocytic stages in vitro. It is encouraging that the activity of the most active CQ derivatives **14** and **16** against the CQ-resistant strain, *Pf*Dd2, was comparable to that of the parent drug. Taken together, our results indicate that the quinoline core influences the profile of activity/toxicity for PQ and CQ fumardiamides. Due to their biological profile and favourable drug-like properties, quinoline fumardiamides could provide a strong basis for further optimisation towards the development of novel and safer antiplasmodial drugs.

## Figures and Tables

**Table 1:** IC_50_ values for PQ (**1**–**6**) and CQ (**11**–**16**) fumardiamides against erythrocytic stages of two *P. falciparum* strains (*Pf*3D7 and *Pf*Dd2) and hepatic stages of *P. bergei.*

Compd.	Structure	IC_50_^1^ (µM) *Pf*3D7	IC_50_ (µM) *Pf*Dd2	IC_50_ (µM) *P. bergei*
**1**	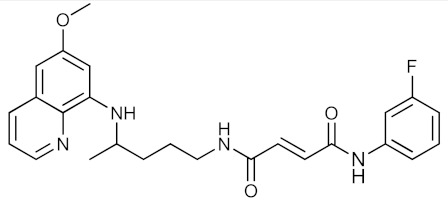	13.77	20	n.d. ^2^
**2**	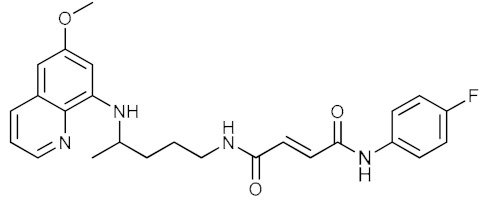	7.74	>13	n.d.
**3**	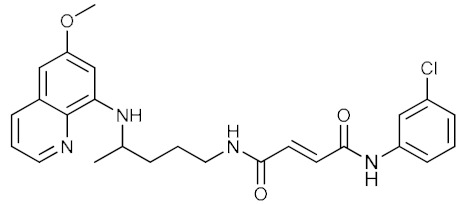	>13	13.58	0.27 ± 0.06 ^3^
**4**	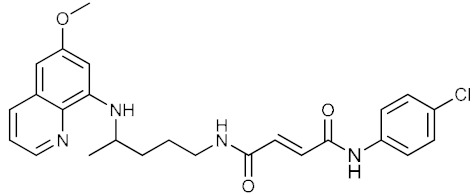	>13	>13	0.39 ± 0.01
**5**	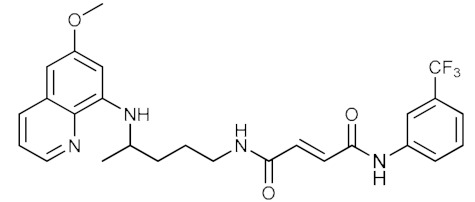	13.91	10.67	0.27 ± 0.08
**6**	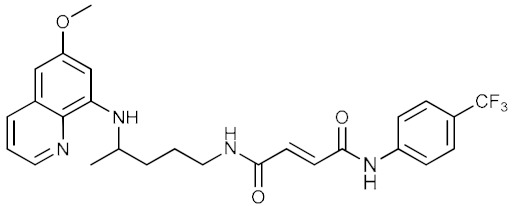	12.96	18.43	0.11 ± 0.02
**11**	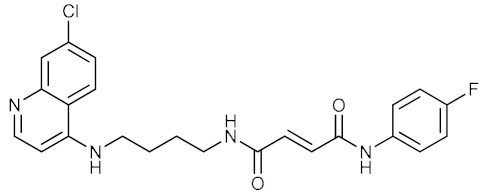	0.144	7.024	n.d.
**12**	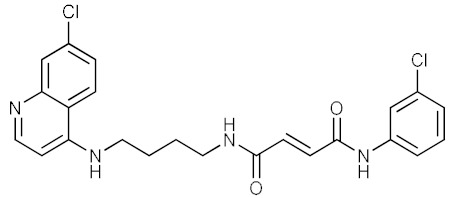	0.067	1.879	n.d.
**13**	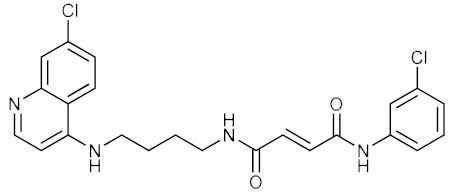	0.083	6.961	n.d.
**14**	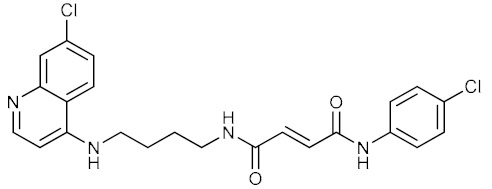	0.035	0.89	n.d.
**15**	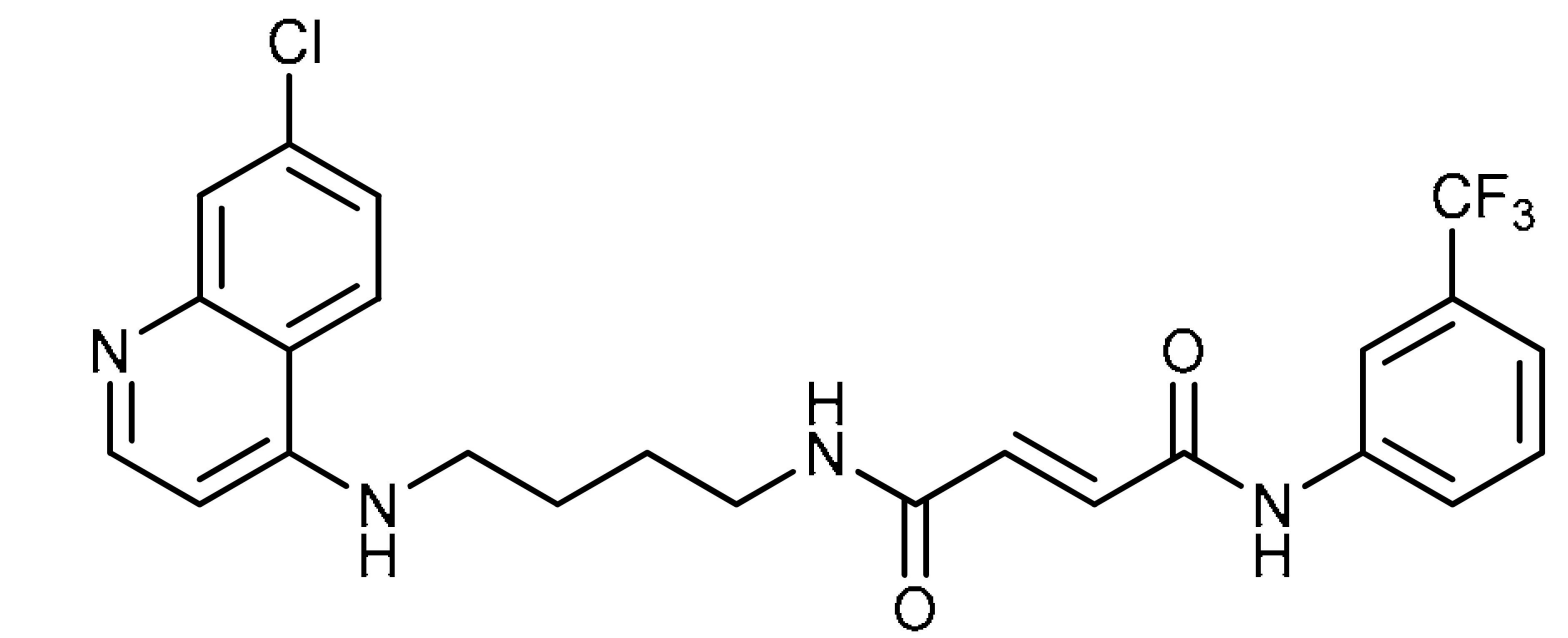	0.194	3.217	n.d.
**16**	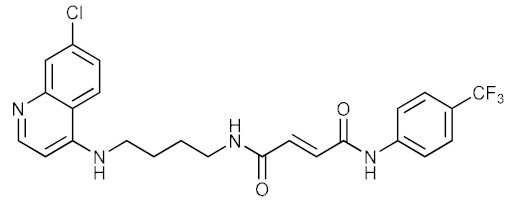	0.035	0.379	n.d.
PQ ^4^		1.994	1.816	8.4 ± 3.4
CQ ^5^		0.0037	0.241	n.d.

^1^ IC_50_, the concentration of the tested compound necessary for 50% growth inhibition. ^2^ n.d., not determined. ^3^ Results represent mean ± SD, *n* = 1. ^4^ PQ, primaquine. ^5^ CQ, chloroquine.

**Table 2:** IC_50_ values for PQ fumardiamides **5** and **6** tested in vitro on MCF-7 and HEK293T cell lines, alone or in combination with 6-aminonicotinamide.

Compd.	IC_50_^1^(µM) MCF-7	IC_50_^2^ (µM) HEK293T
**5**	≥100	≥100
**6**	≥100	≥100
6-AN + **5**	≥100	≥100
6-AN + **6**	≥100	≥100
6-AN	2 ± 0.3	10 ± 7

^1^ IC_50_, the concentration of the tested compound necessary for 50% growth inhibition. ^2^ 6-AN, 6-aminonicotinamide, *c* = 1 µM.

**Table 3:** Properties of novel compounds calculated with Chemicalize.org program [22]. The Lipinski’s and Gelovani’s parameters.

Compd.	Molecular Formula	Number of Atoms	MW	log *P*	HBD	HBA	Lipinski Score ^1^	MR (cm^3^/mol)	TPSA (Å^2^)
**11**	C_23_H_22_ClFN_4_O_2_	53	440.90	3.57	3	4	4	122.34	83.12
**12**	C_23_H_22_ClFN_4_O_2_	53	440.90	3.57	3	4	4	122.34	83.12
**13**	C_23_H_22_Cl_2_N_4_O_2_	53	457.36	4.03	3	4	4	126.93	83.12
**14**	C_23_H_22_Cl_2_N_4_O_2_	53	457.36	4.03	3	4	4	126.93	83.12
**15**	C_24_H_22_ClF_3_N_4_O_2_	56	490.91	4.30	3	4	4	128.10	83.12
**16**	C_24_H_22_ClF_3_N_4_O_2_	56	490.91	4.30	3	4	4	128.10	83.12

MW—molecular weight; log *P*—partition coefficient; HBD—number of H-bond donors; HBA—number of H-bond acceptors; MR—molecular refractivity; TPSA—topological polar surface area; ^1^ out of four.

## Supplementary Materials

Spectra of all compounds are available online. Figure S1: Structural formula, MS, IR, ^1^H and ^13^C spectra of compounds **9**–**16**. Table S1: Analytical and spectral data of compounds **9**–**16**, Table S2: ^1^H and ^13^C NMR spectra of compounds **9**–**16**.

## Abbreviations

6-AN, 6-aminonicotinamide; CQ, chloroquine; DCM, dichloromethane; DIEA, *N,N*-diisopropylethylamine; DMEM, Dulbecco’s modified Eagle’s medium; DMSO, dimethyl sulfoxide; FBS, foetal bovine serum; G6PD, glucose-6-phosphate dehydrogenase, HATU, 1-[bis(dimethylamino)methylene]-1*H*-1,2,3-triazolo[4,5-*b*]pyridinium 3-oxide hexafluorophosphate; HEK293T, non-tumour embryonic kidney cell line; Huh7, human hepatoma cell line; HBA, number of H-bond acceptors; HBD, number of H-bond donors; HRP2, histidine-rich protein 2; IC_50_, the concentration of the tested compound necessary for 50% growth inhibition; log *P*, partition coefficient; MCF-7, breast adenocarcinoma cell line; MR, molar refractivity; MTT, (3-(4,5-dimethylthiazol-2-yl)-2,5-diphenyltetrazolium bromide; MW, microwave irradiation; PAINS, pan assay interference compounds; PQ, primaquine; RPMI, Roswell Park Memorial Institute; SOCl_2_, thionyl chloride, TEA, triethylamine, TQ, tafenoquine; TPSA, topological polar surface area.
