# Supplementary material for: Primaquine and Chloroquine Fumardiamides as Promising Antiplasmodial Agents
Source: Molecules. 2019 Aug 1;24(15):2812. doi: 10.3390/molecules24152812 (PMC6695747; doi:10.3390/molecules24152812)
Supplement: Supplementary file 1 [file molecules-24-02812-s001.pdf]

Supporting Information

SI Table 1. Analytical and spectral data of compounds **9-16**

Compd.	Yield (%)	m.p. (°C)	IR (KBr): ν_{max} (cm^{-1})	MS (m/z)	Molecular formula (M_r)	CHN analysis calcd./found (%)		
**9**	73	207–209	2566, 3368, 3282, 3082, 2946, 2872, 2796, 1722, 1672, 1643, 1584, 1454, 1368, 1334, 1302, 1238, 1176, 1080, 1022, 974, 896, 850, 806, 766, 692, 666, 642, 570, 540.	376.1 (M+1)	$C_{19}H_{22}ClN_3O_3$ (375.15)	60.72<br>60.58	5.90<br>5.51	11.18<br>11.38
**10**	91	227–228	3280, 3070, 3940, 2872, 2364, 2060, 1956, 1620, 1558, 1456, 1366, 1206, 1136, 1090, 984, 900, 816, 762, 658, 584.	348.0 (M+1)	$C_{17}H_{18}ClN_3O_3$ (347.10)	58.71<br>59.12	5.22<br>5.12	12.08<br>11.97
**11**	25	229–232	3634, 3282, 3092, 2938, 3868, 1650, 1606, 1558, 1490, 1428, 1384, 1338, 1262, 1212, 1186, 1136, 1080, 1042, 1020, 970, 938, 902, 854, 810, 772, 716, 678, 594, 520.	441.0 (M+1)	$C_{23}H_{22}ClFN_4O_2$ (440.90)	62.66<br>62.70	5.03<br>5.15	12.71<br>12.79
**12**	81	239 (decomp)	3284, 3072, 2956, 2932, 2864, 1636, 1582, 1542, 1510, 1452, 1406, 1368, 1334, 1278, 1212, 1172, 1140, 1080, 996, 934, 902, 834, 764, 690, 552, 516.	441.0 (M+1)	$C_{23}H_{22}ClFN_4O_2$ (440.90)	62.66<br>62.45	5.03<br>5.25	12.71<br>12.53
**13**	26	232 (decomp)	3340, 3298, 3182, 3092, 3032, 2944, 2866, 2830, 2360, 1650, 1592 1542, 1482, 1426, 1334, 1234, 1214, 1170, 1142, 1078, 1034, 970, 900, 848, 808, 782, 678, 626, 588, 558.	457.0 (M+1)	$C_{23}H_{22}Cl_2N_4O_2$ (457.35)	60.40<br>60.26	4.85<br>4.62	12.25<br>12.27
**14**	71	237 (decomp)	3412, 3286, 3186, 3098, 3072, 2946, 2878, 2706, 2364, 1363, 1544, 1492, 1452, 1398, 1342, 1240, 1212, 1172, 1128, 1094, 1014, 986, 842, 76, 680, 584, 558, 506.	457.0 (M+1)	$C_{23}H_{22}Cl_2N_4O_2$ (457.35)	60.40<br>60.34	4.85<br>4.97	12.25<br>12.13
**15**	26	240 (decomp)	3418, 3210, 3072, 3002, 2958, 1620, 1550, 1490, 1414, 1312, 1182, 1136, 1068, 1022, 840, 656, 558.	491.1 (M+1)	$C_{24}H_{22}ClF_3N_4O_2$ (490.91)	58.72<br>58.90	4.52<br>4.55	11.41<br>11.58
**16**	37	270 (decomp)	3254, 2972, 2914, 2836, 2760, 2722, 2546, 2488, 2426, 2362, 2098, 1976, 1712, 1610, 1590, 1544, 1472, 1442, 1398, 1324, 1210, 1154, 1104, 1066, 1018, 978, 956, 882, 842, 802, 768, 686, 650, 592, 508.	491.1 (M+1)	$C_{24}H_{22}ClF_3N_4O_2$ (490.91)	58.72<br>58.96	4.52<br>4.73	11.41<br>11.67

SI Table 2. ^{1}H and ^{13}C NMR spectra of compounds **9-16**

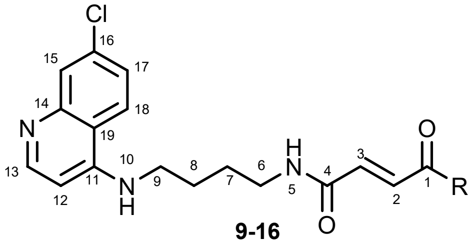

9-16

Compd.	R	^{1}H NMR (DMSO-d_6, δ ppm, J/Hz)	^{13}C NMR (DMSO-d_6, δ ppm, J/Hz)
**9**	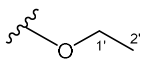	8.56 (t, 1H, 5, *J* = 5.5 Hz), 8.39 (d, 1H, 13, *J* = 5.5 Hz), 8.29 (d, 1H, 18, *J* = 9.1 Hz), 7.79 (d, 1H, 15, *J* = 2.2 Hz), 7.49-7.39 (m, 2H, 17, 10), 7.00 (d, 1H, 3, *J* = 15.5 Hz), 6.56 (d, 1H, 2, *J* = 15.5 Hz), 6.50 (d, 1H, 12, *J* = 5.6 Hz), 4.18 (q, 2H, 1', *J* = 7.1 Hz), 3.35-3.16 (m, 4H, 6, 9), 1.75-1.50 (m, 4H, 7, 8), 1.24 (t, 3H, 2', *J* = 7.1 Hz).	165.08 (4), 162.73 (1), 151.41 (13), 150.35 (14), 148.51 (11), 137.61 (3), 133.61 (16), 128.18 (2), 127.02 (17), 124.19 (18), 124.14 (15), 117.34 (19), 98.67 (12), 60.67 (1’), 42.04 (9), 38.55 (6), 26.48 (7), 25.18 (8), 14.01 (2’).
**10**	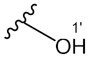	8.47 (t, 1H, 5, *J* = 5.5 Hz), 8.38 (d, 1H, 13, *J* = 5.5 Hz), 8.27 (d, 1H, 18, *J* = 9.1 Hz), 7.77 (d, 1H, 15, *J* = 2.2 Hz), 7.52-7.33 (m, 2H, 17, 10), 6.88 (d, 1H, 3, *J* = 15.5 Hz), 6.55-6.45 (m, 2H, 2, 12), 3.35-3.09 (m, 4H, 6, 9), 1.77-1.48 (m, 4H, 7, 8).	166.71 (4), 163.21 (1), 151.50 (13), 150.32 (14), 148.59 (11), 136.60 (3), 133.57 (16), 130.12 (2), 127.09 (17), 124.18 (18), 124.13 (15), 117.36 (19), 98.67 (12), 42.05 (9), 38.50 (6), 26.55 (7), 25.21 (8).
**11**	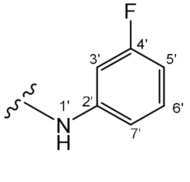	10.77 (s, 1H, 1'), 8.68-8.40 (m, 4H, 5, 13, 18, 3'), 7.92 (d, 1H, 15, *J* = 1.4 Hz), 7.70 (d, 1H, 17, *J* = 11.6 Hz), 7.62 (dd, 1H, 10, *J* = 9.0, 1.7 Hz), 7.38 (m, 2H, 6', 7'), 7.03 (q, 2H, 2, 3, *J* = 15.1 Hz), 6.93 (t, 1H, 5', *J* = 8.49 Hz), 6.72 (d, 1H, 12, *J* = 6.4 Hz), 3.48-3.40 (m, 2H, 9), 3.29-3.21 (m, 2H, 6), 1.76-1.66 (m, 2H, 7), 1.64-1.55 (m, 2H, 8).	163.69-160.49 (d, 4’, *J* = 242.80 Hz), 163.39 (4), 162.62 (1), 154.77 (14), 143.78 (13), 140.61-140.46 (d, 2’, *J* = 11.38 Hz), 139.69 (11), 137.42 (16), 134.32 (3), 132.39 (2), 130.54-130.42 (d, 6’, *J* = 9.34 Hz), 126.47 (17), 125.60 (18), 119.96 (15), 115.65 (19), 115.18 (7’), 110.42-110.14 (d, 3’, *J* = 20.55), 106.33-105.98 (d, 5’, *J* = 20.55 Hz), 98.61 (12), 42.73 (9), 38.37 (6), 26.34 (7), 25.04 (8).
**12**	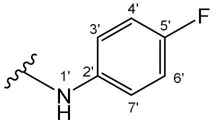	10.58 (s, 1H, 1'), 8.55 (t, 1H, 5, *J* = 37 Hz), 8.41 (d, 1H, 13, *J* = 5.6 Hz), 8.34 (d, 1H, 18, *J* = 9.1 Hz), 7.80 (d, 1H, 15, *J* = 2.1 Hz), 7.72 (dd, 2H, 3', 7', *J* = 9.0, 5.0 Hz), 7.59 (t, 1H, 17, *J* = 5.47 Hz), 7.47 (dd, 1H, 10, *J* = 9.0, 2.2 Hz), 7.17 (t, 2H, 4', 6', *J* = 9.13 Hz), 7.02 (q, 2H, 2, 3, *J* = 15.1 Hz), 6.53 (d, 1H, 12, *J* = 5.7 Hz), 3.40-3.19 (m, 4H, 6, 9), 1.75-1.52 (m, 4H, 7, 8).	163.44 (4), 162.23 (1), 159.85-156.66 (d, 5’, *J* = 241.00 Hz), 150.96 (13), 150.62 (14), 148.03 (11), 135.30 (2’), 133.97 (3), 133.79 (16), 132.60 (2), 126.59 (17), 124.35 (18), 124.23 (15), 121.14-121.03 (d, 3’, 7’, *J* = 7.71 Hz), 117.26 (19), 115.56-115.27 (d, 4’, 6’, *J* = 22.77 Hz), 98.64 (12), 42.09 (9), 38.49 (6), 26.56 (7), 25.20 (8).
**13**	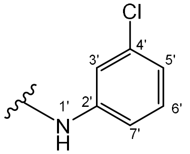	10.67 (s, 1H, 1'), 8.56 (t, 1H, 5, *J* = 5.5 Hz), 8.43 (d, 1H, 13, *J* = 5.9 Hz), 8.38 (d, 1H, 18, *J* = 9.1 Hz), 8.01-7.87 (m, 2H, 3', 7'), 7.83 (d, 1H, 15, *J* = 2.1 Hz), 7.66-7.46 (m, 2H, 17, 6'), 7.37 (t, 1H, 10, *J* = 8.1 Hz), 7.15 (dd, 1H, 5', *J* = 7.9, 1.1 Hz), 7.02 (q, 2H, 2, 3, *J* = 14.5 Hz), 6.60 (d, 1H, 12, *J* = 6.0 Hz), 3.42-3.32 (m, 2H, 9), 3.29-3.19 (m, 2H, 6), 1.76-1.53 (m, 4H, 7, 8).	163.34 (4), 162.64 (1), 151.57 (14), 149.35 (13), 146.10 (11), 140.25 (4’), 134.66 (2’), 134.42 (3), 133.14 (16), 132.33 (2), 130.56 (17), 125.10 (6’), 124.79 (18), 124.56 (15), 123.53 (5’), 118.79 (3’), 117.78 (7’), 116.88 (19), 98.66 (12), 42.26 (9), 38.50 (6), 26.52 (7), 25.18 (8).
**14**	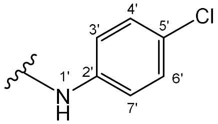	10.71 (s, 1H, 1'), 9.51 (t, 1H, 5, *J* = 5.2 Hz), 8.66 (d, 1H, 13, *J* = 9.2 Hz), 8.60 (t, 1H, 17, *J* = 5.6 Hz), 8.55 (d, 1H, 18, *J* = 7.1 Hz), 8.04 (d, 1H, 15, *J* = 2.0 Hz), 7.75 (dd, 3H, 10, 3', 7', *J* = 12.8, 5.5 Hz), 7.39 (d, 2H, 4', 6', *J* = 8.9 Hz), 7.02 (q, 2H, 2, 3, *J* = 15.1 Hz), 6.89 (d, 1H, 12, *J* = 7.2 Hz), 3.6-3.5 (m, 2H, 9), 3.27-3.14 (m, 2H, 6), 1.79-1.66 (m, 2H, 7), 1.65-1.53 (m, 2H, 8).	163.43 (4), 162.42 (1), 155.17 (14), 143.06 (13), 138.87 (11), 137.81 (2’, 5’), 134.13 (3), 132.50 (2), 128.73 (15), 127.35 (16), 126.68 (17), 125.74 (18), 120.87 (4’, 6’), 119.30 (3’, 7’), 115.50 (19), 98.59 (12), 42.79 (9), 38.34 (6), 26.32 (7), 25.01 (8).
**15**	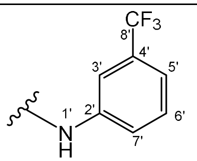	10.81 (s, 1H, 1'), 9.26 (t, 1H, 5, *J* = 5.2 Hz), 8.62-8.53 (m, 4H, 13, 18, 3', 7'), 7.94 (d, 1H, 15, *J* = 2.1 Hz), 7.86 (d, 1H, 5', *J* = 8.4 Hz, 1H), 7.81 (dd, 1H, 6', *J* = 9.1, 2.1 Hz), 7.62 (t, 1H, 17, *J* = 8.0 Hz), 7.49 (d, 1H, 10, *J* = 7.8 Hz), 7.04 (q, 2H, 2, 3, *J* = 14.88 Hz), 6.93 (d, 1H, 12, *J* = 7.1 Hz), 3.61-3.54 (m, 2H, 9), 3.32-3.25 (m, 2H, 6), 1.81-1.70 (m, 2H, 7), 1.68-1.58 (m, 2H, 8).	163.31 (4), 162.78 (1), 155.08 (14), 143.56 (13), 139.54 (11), 139.14 (2'), 137.77 (16), 134.47 (3), 132.27 (2), 130.17 (6'), 129.64-129.38 (q, 4', *J* = 26.85 Hz), 126.75 (15), 125.45 (18), 122.93 (17), 122.71-120.00 (q, 8', *J* = 274.54 Hz), 120.22 (7'), 119.67 (3'), 115.53 (19), 115.36 (5'), 98.68 (12), 42.85 (9), 38.41 (6), 26.34 (7), 25.06 (8).

**16**	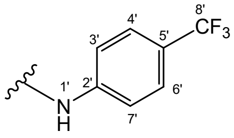	10.92 (s, 1H, 1'), 9.30 (t, 1H, 5, *J* = 2.54 Hz), 8.71-8.57 (m, 2H, 13, 18), 8.54 (d, 1H, 17, *J* = 6.9 Hz), 8.01 (d, 1H, 15, *J* = 2.0 Hz), 7.91 (d, 2H, 4', 6', *J* = 8.5 Hz), 7.73 (dd, 3H, 10, 3', 7', *J* = 14.2, 5.2 Hz), 7.06 (q, 2H, 2, 3, *J* = 15.1 Hz), 6.86 (d, 1H, 12, *J* = 7.0 Hz), 3.57-3.49 (m, 2H, 9), 3.29-3.21 (m, 2H, 6), 1.77-1.67 (m, 2H, 7), 1.64-1.54 (m, 2H, 8).	163.35 (4), 162.88 (1), 155.08 (14), 143.26 (13), 142.39 (11), 139.08 (2’), 137.70 (16), 134.62 (3), 132.29 (2), 126.65 (15), 126.17 (3’, 7’), 125.70 (17, 18), 123.95-123.52 (q, 5’, *J* = 25.48 Hz), 122.53-118.93 (q, 8’, *J* = 270.75 Hz), 119.48-119.33 (q, 4’, 6’, *J* = 10.26), 115.54 (19), 98.61 (12), 42.79 (9), 38.38 (6), 26.33 (7), 25.03 (8).

Figure S1. Structural formula, MS, IR, ^{1}H and ^{13}C spectra of compounds **9-16**.

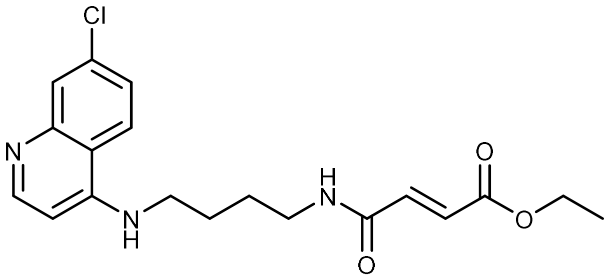

9

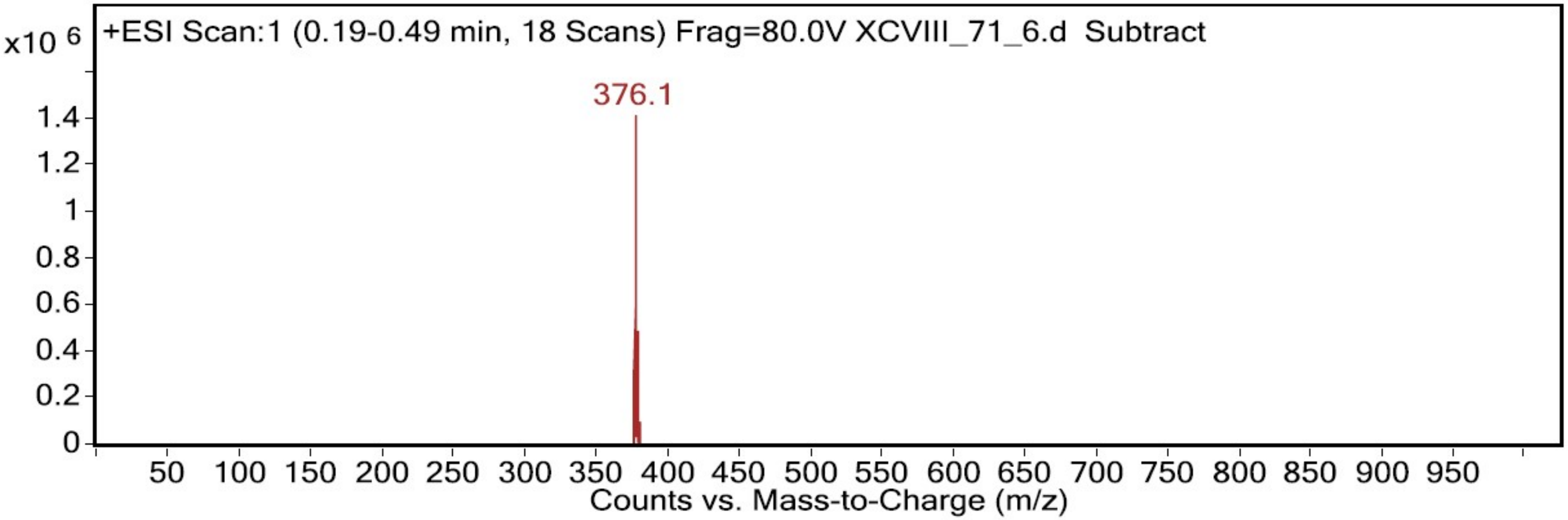

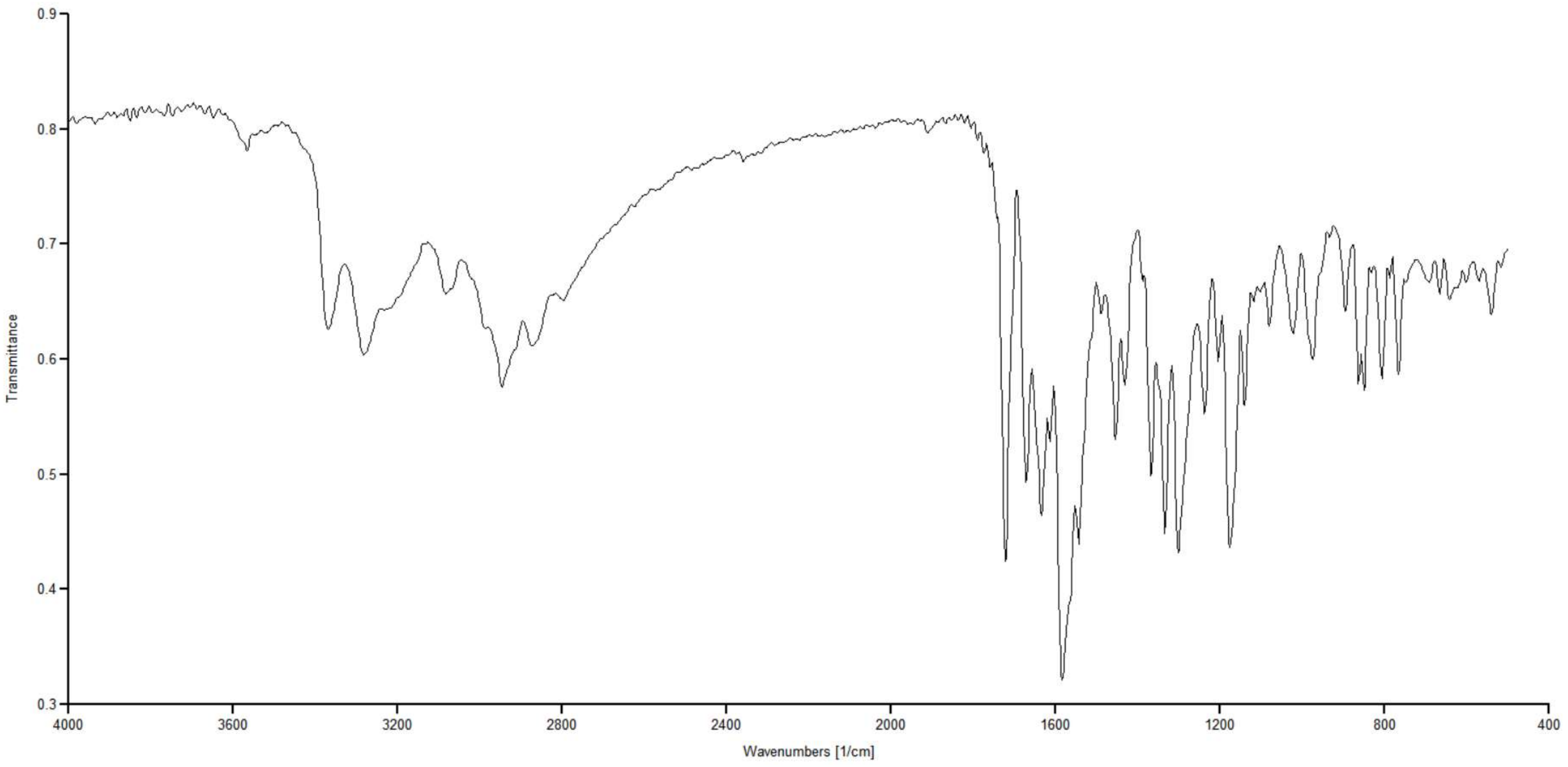
0.9
0.8
0.7
0.6
0.5
0.4
0.3
Transmittance
4000
3600
3200
2800
2400
2000
1600
1200
800
400
Wavenumbers [1/cm]

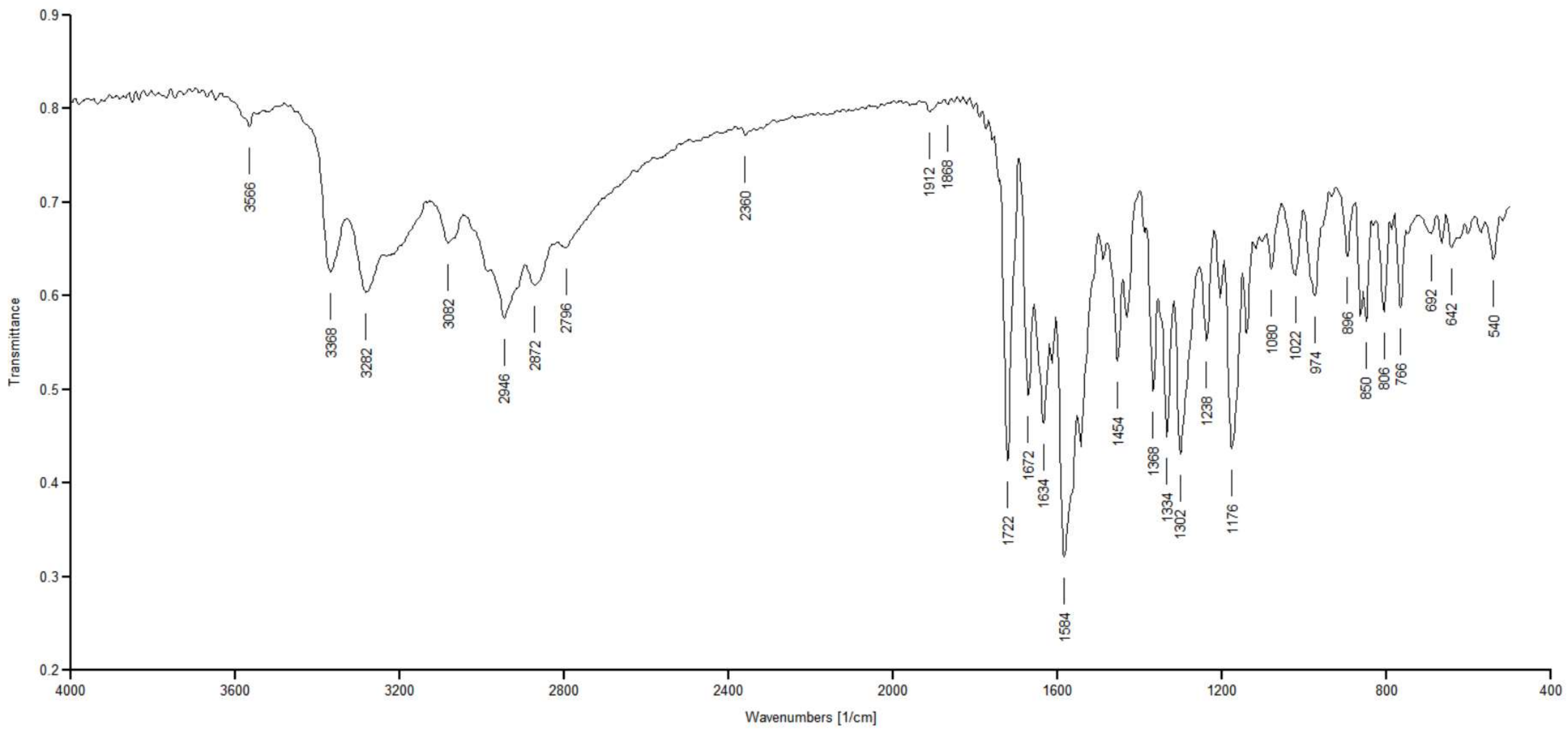
Transmittance
Wavenumbers [1/cm]
0.9
0.8
0.7
0.6
0.5
0.4
0.3
0.2
4000
3600
3200
2800
2400
2000
1600
1200
800
400
3566
3368
3282
3082
2946
2872
2796
2360
1912
1868
1722
1672
1634
1584
1454
1368
1334
1302
1238
1176
1080
1022
974
896
850
806
766
692
642
540

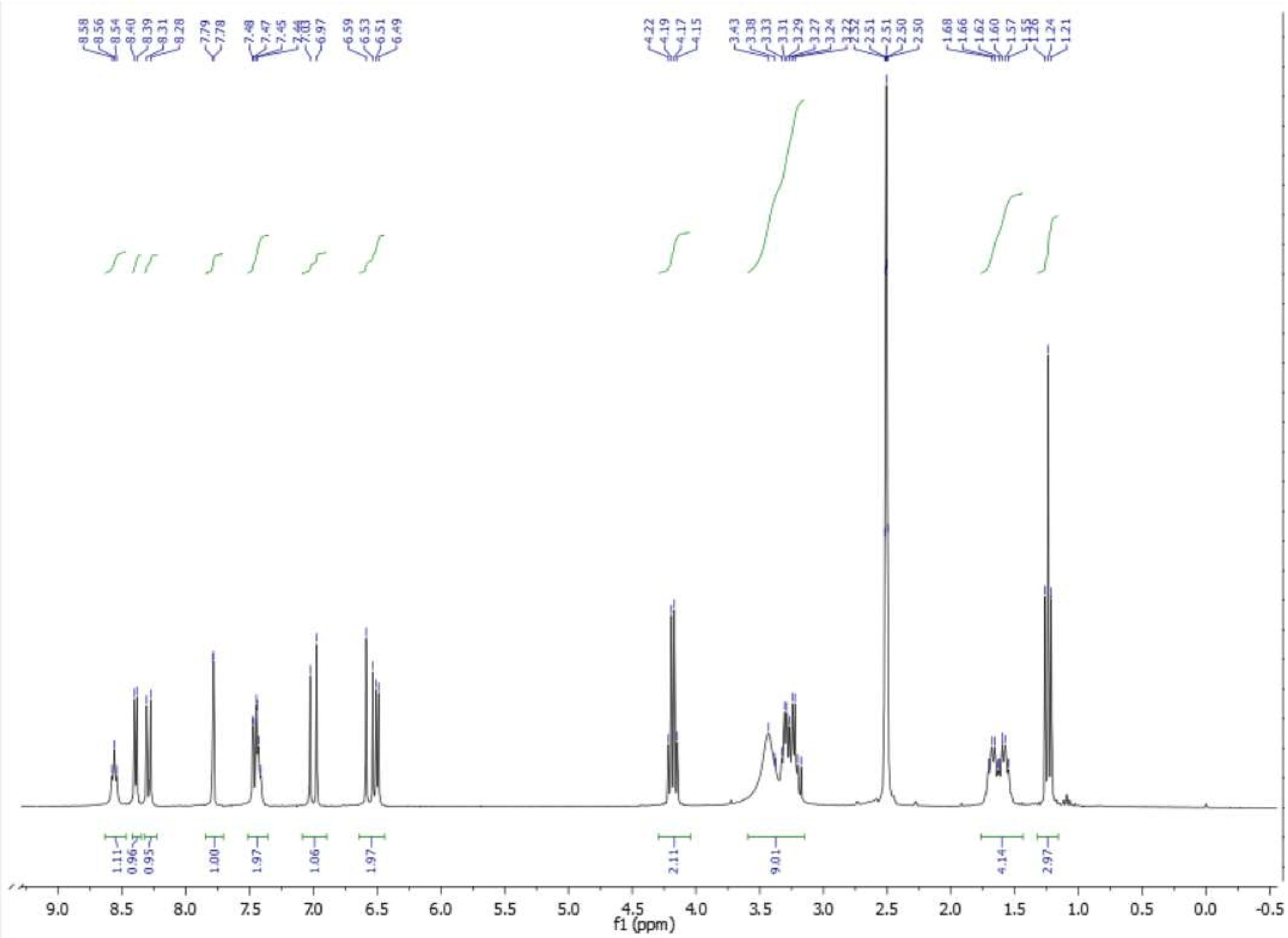
f1 (ppm)

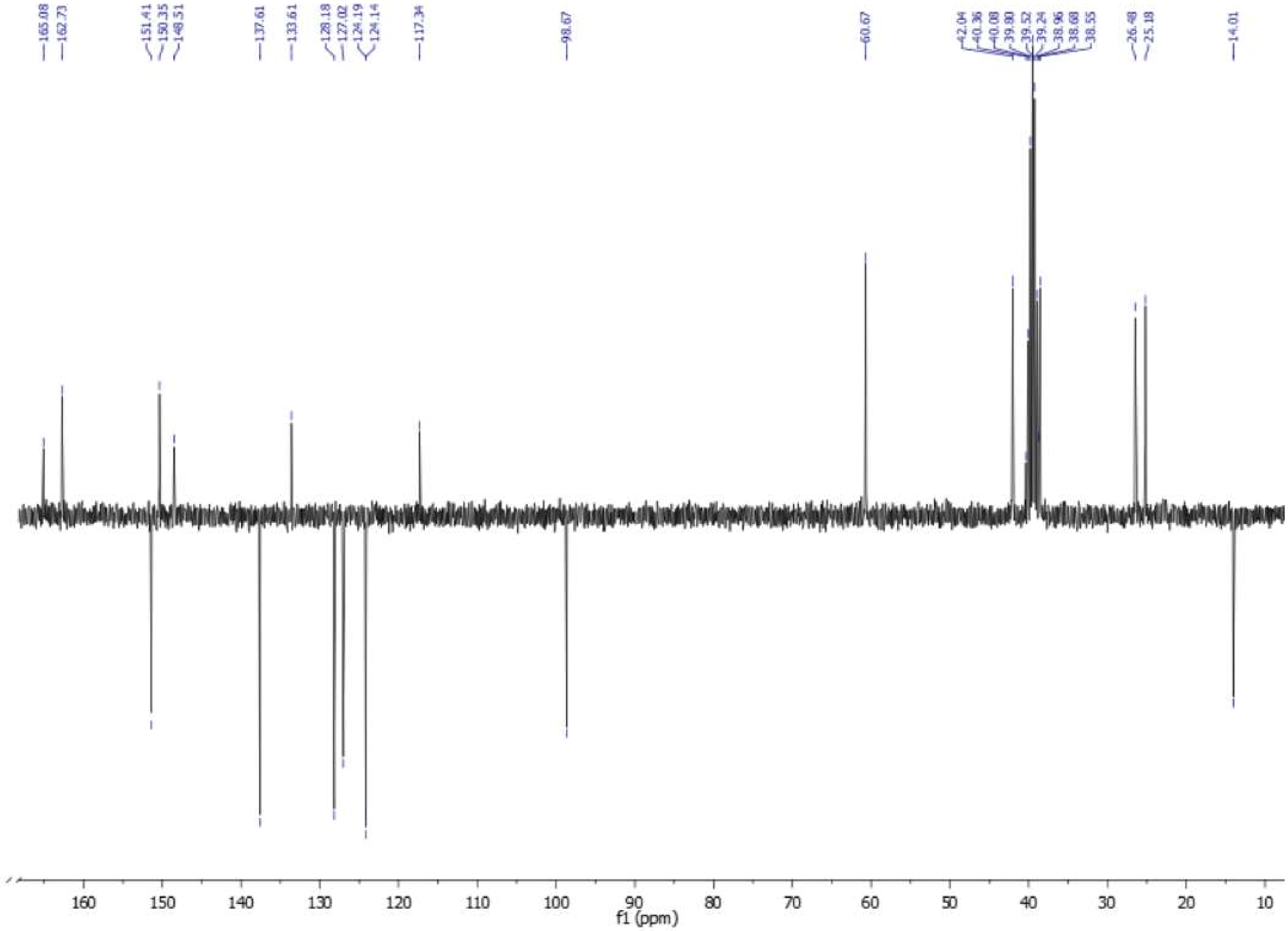

165.08
162.73
151.41
150.35
148.51
137.61
133.61
128.18
127.02
124.19
124.14
117.34
98.67
60.67
42.04
40.36
40.08
39.80
39.52
39.24
38.96
38.68
38.55
26.48
25.18
14.01
160
150
140
130
120
110
100
90
80
70
60
50
40
30
20
10
f1 (ppm)

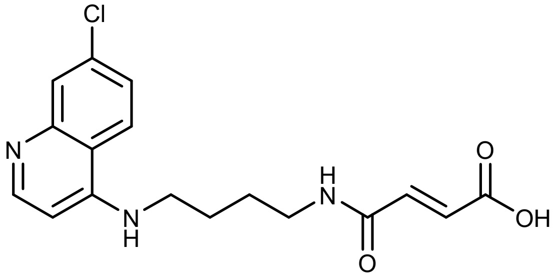
Cl
N
N
H
H
N
O
O
OH

10

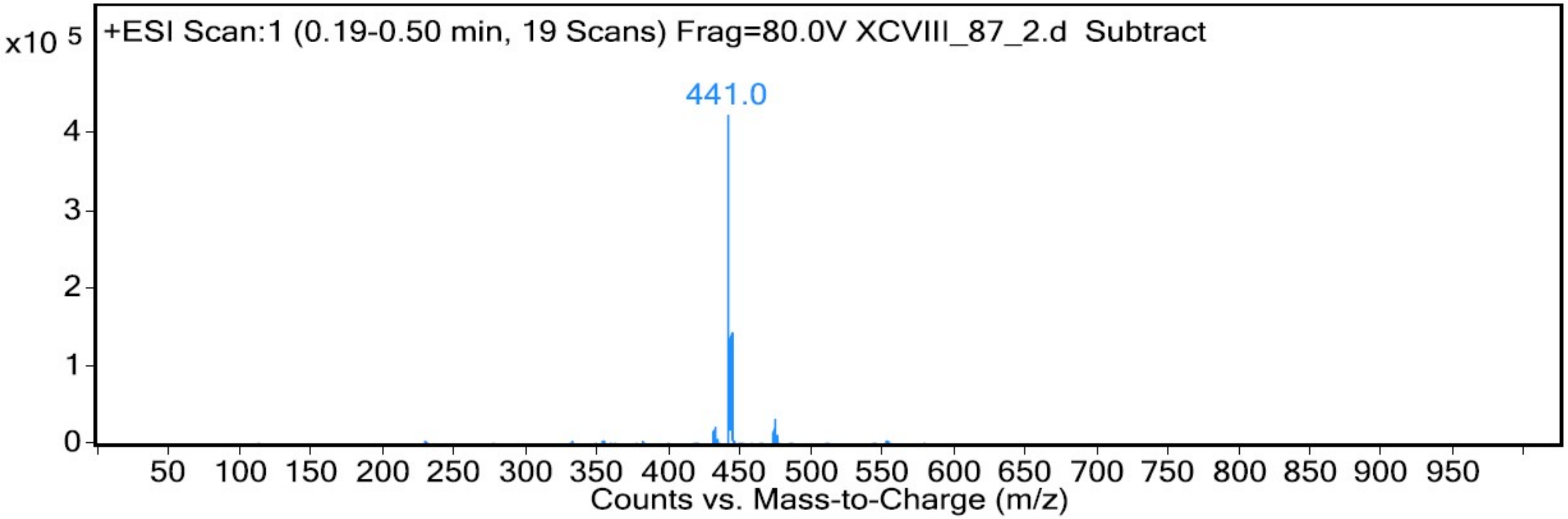
x10 5
+ESI Scan:1 (0.19-0.50 min, 19 Scans) Frag=80.0V XCVIII_87_2.d Subtract
441.0
4
3
2
1
0
50 100 150 200 250 300 350 400 450 500 550 600 650 700 750 800 850 900 950
Counts vs. Mass-to-Charge (m/z)

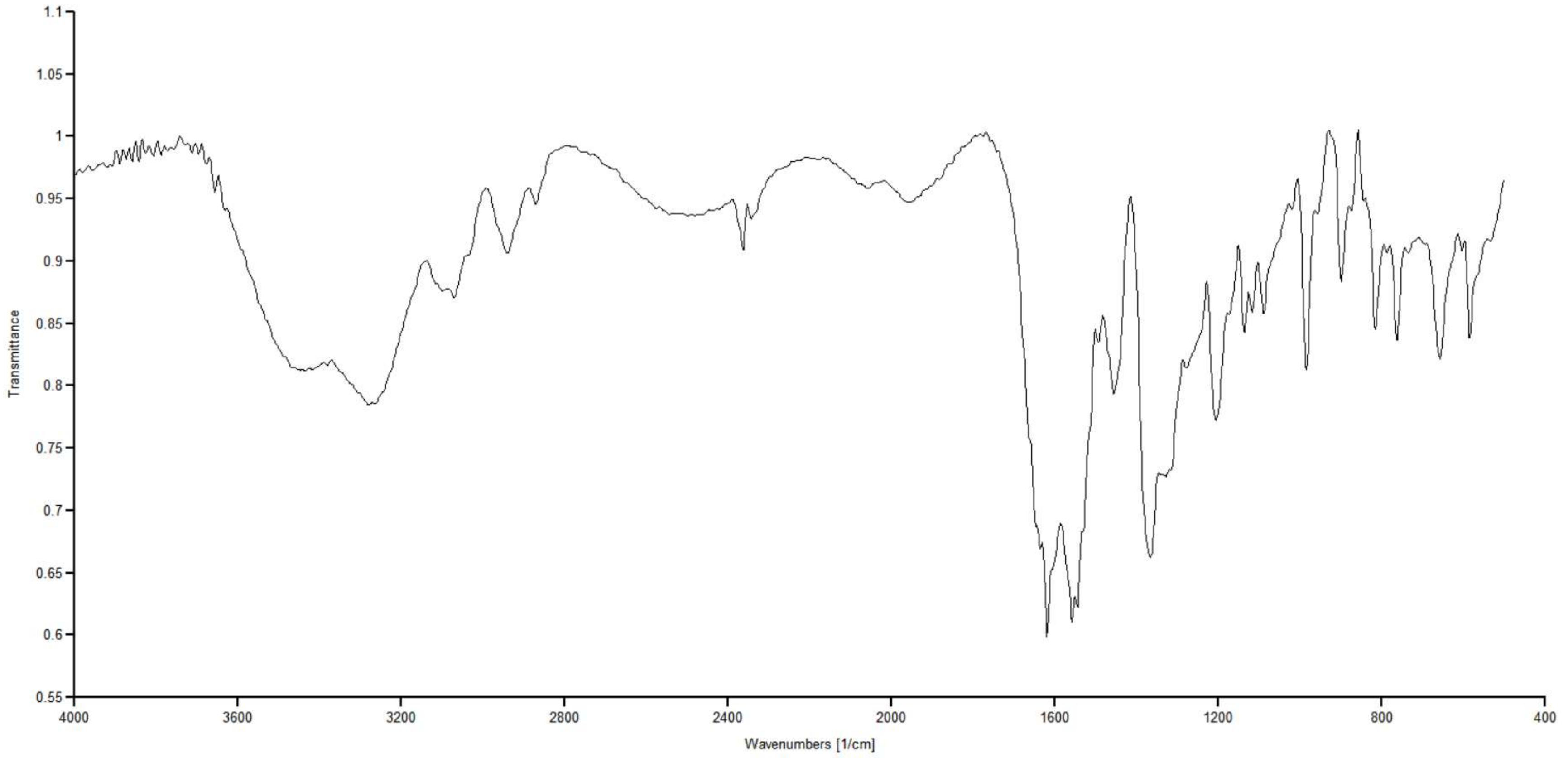
Transmittance
1.1
1.05
1
0.95
0.9
0.85
0.8
0.75
0.7
0.65
0.6
0.55
4000
3600
3200
2800
2400
2000
1600
1200
800
400
Wavenumbers [1/cm]

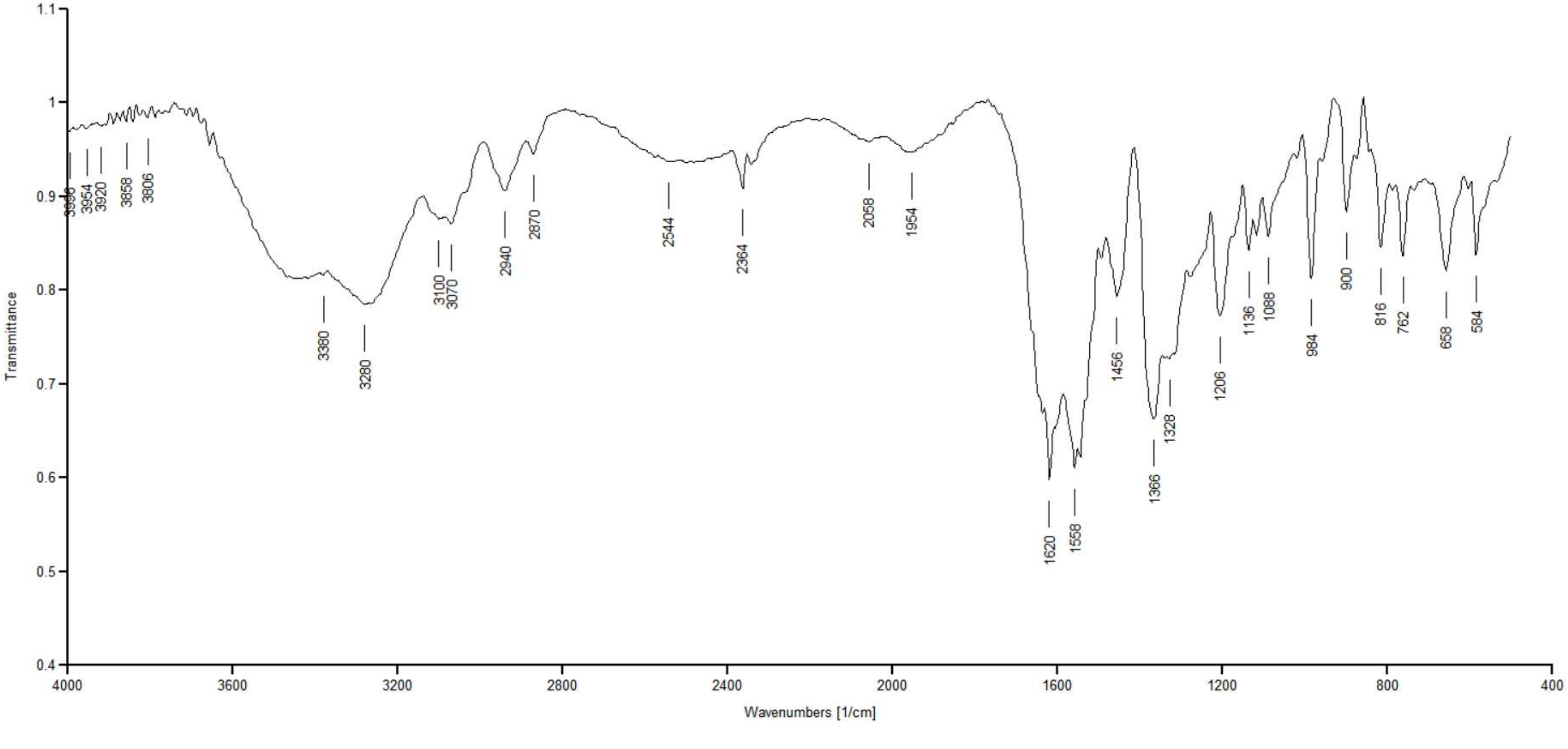
Transmittance
Wavenumbers [1/cm]
3996
3954
3920
3858
3806
3380
3280
3100
3070
2940
2870
2544
2364
2058
1954
1620
1558
1456
1366
1328
1206
1136
1088
984
900
816
762
658
584

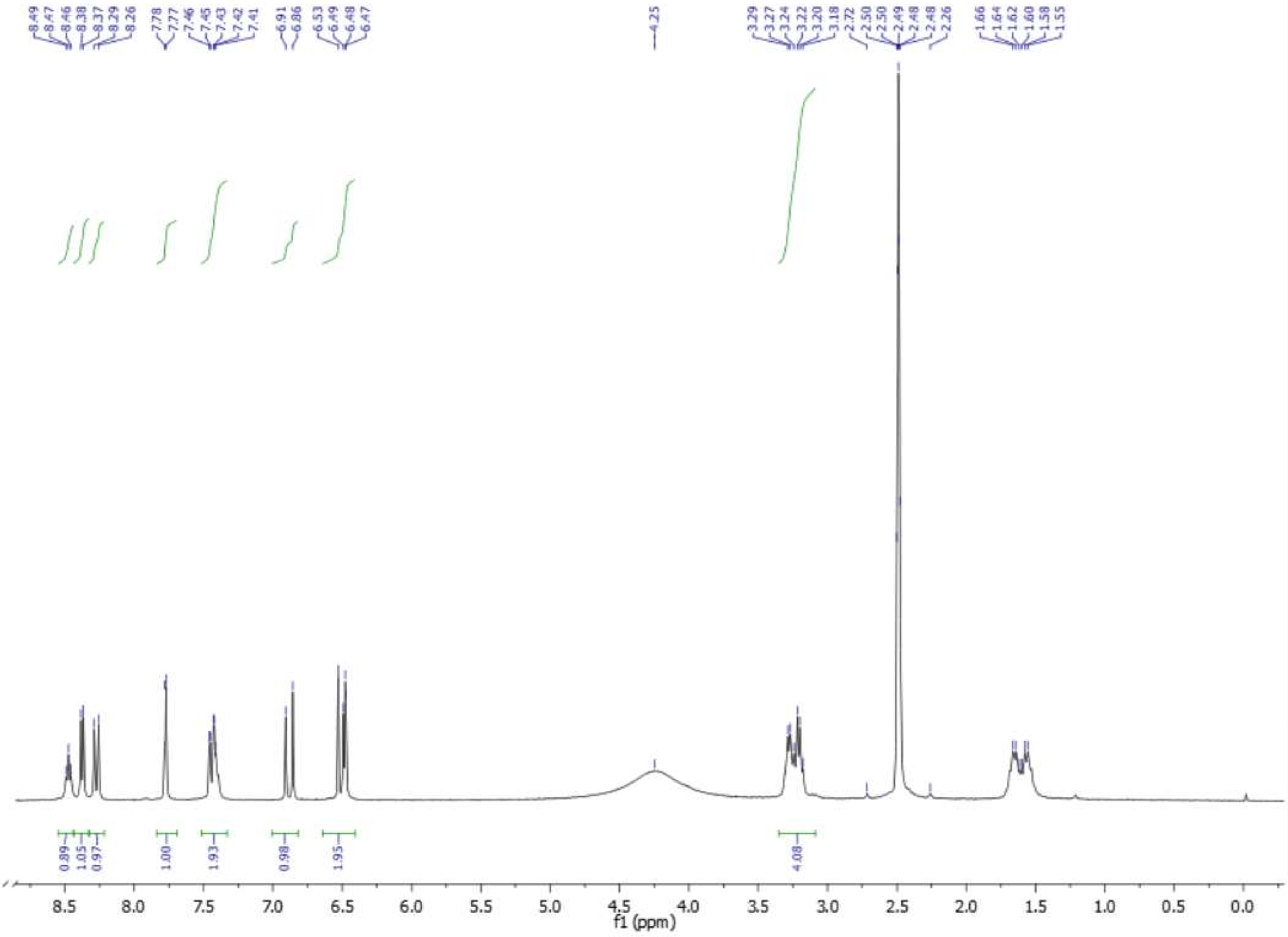

8.49 8.47 8.46 8.38 8.37 8.29 8.26 7.78 7.77 7.46 7.45 7.43 7.42 7.41 6.91 6.86 6.53 6.49 6.48 6.47
4.25
3.29 3.27 3.24 3.22 3.20 3.18 2.72 2.50 2.50 2.49 2.48 2.48 2.26 1.66 1.64 1.62 1.60 1.58 1.55
0.89 1.05 0.97 1.00 1.93 0.98 1.95 4.08
8.5 8.0 7.5 7.0 6.5 6.0 5.5 5.0 4.5 4.0 3.5 3.0 2.5 2.0 1.5 1.0 0.5 0.0
f1 (ppm)

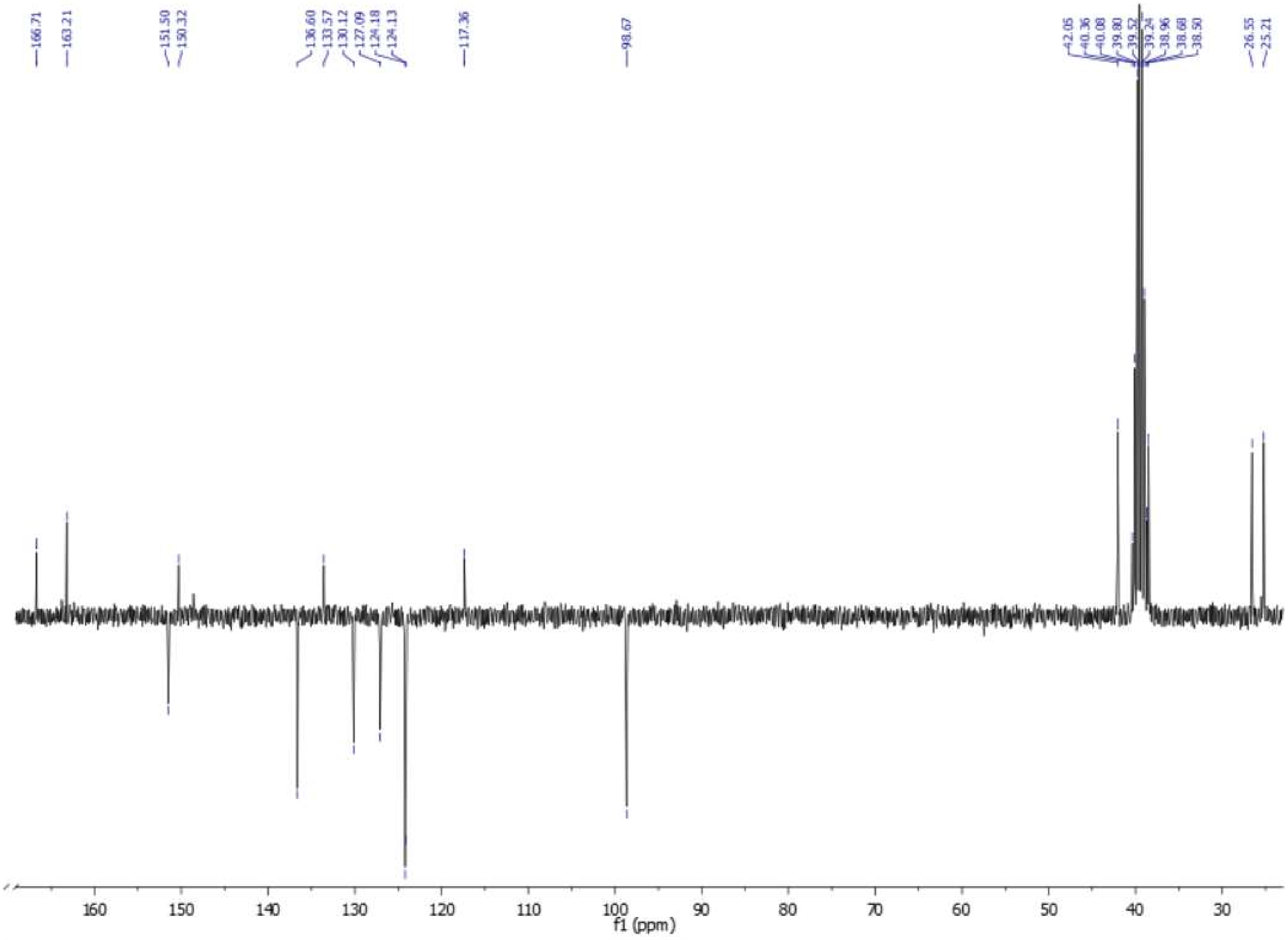

166.71
163.21
151.50
150.32
136.60
133.57
130.12
127.09
124.18
124.13
117.36
98.67
42.05
40.36
40.08
39.80
39.52
39.24
38.96
38.68
38.50
26.55
25.21
160
150
140
130
120
110
100
90
80
70
60
50
40
30
f1 (ppm)

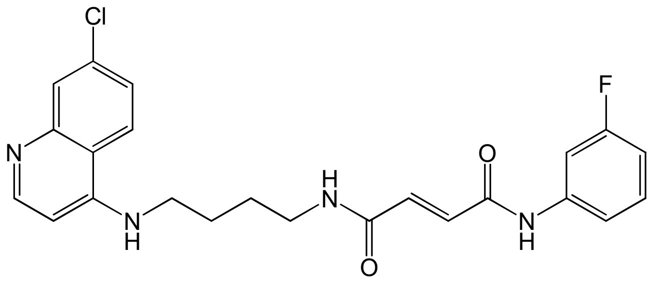
Cl
N
H
N
H
N
O
O
H
N
F

11

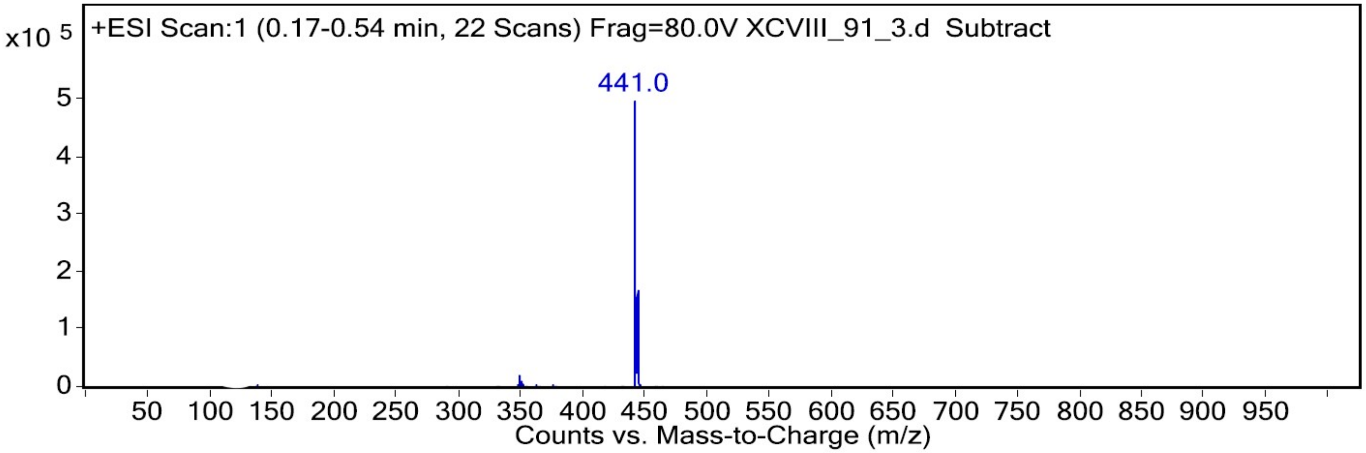
x10 5
+ESI Scan:1 (0.17-0.54 min, 22 Scans) Frag=80.0V XCVIII_91_3.d Subtract
441.0
5
4
3
2
1
0
50 100 150 200 250 300 350 400 450 500 550 600 650 700 750 800 850 900 950
Counts vs. Mass-to-Charge (m/z)

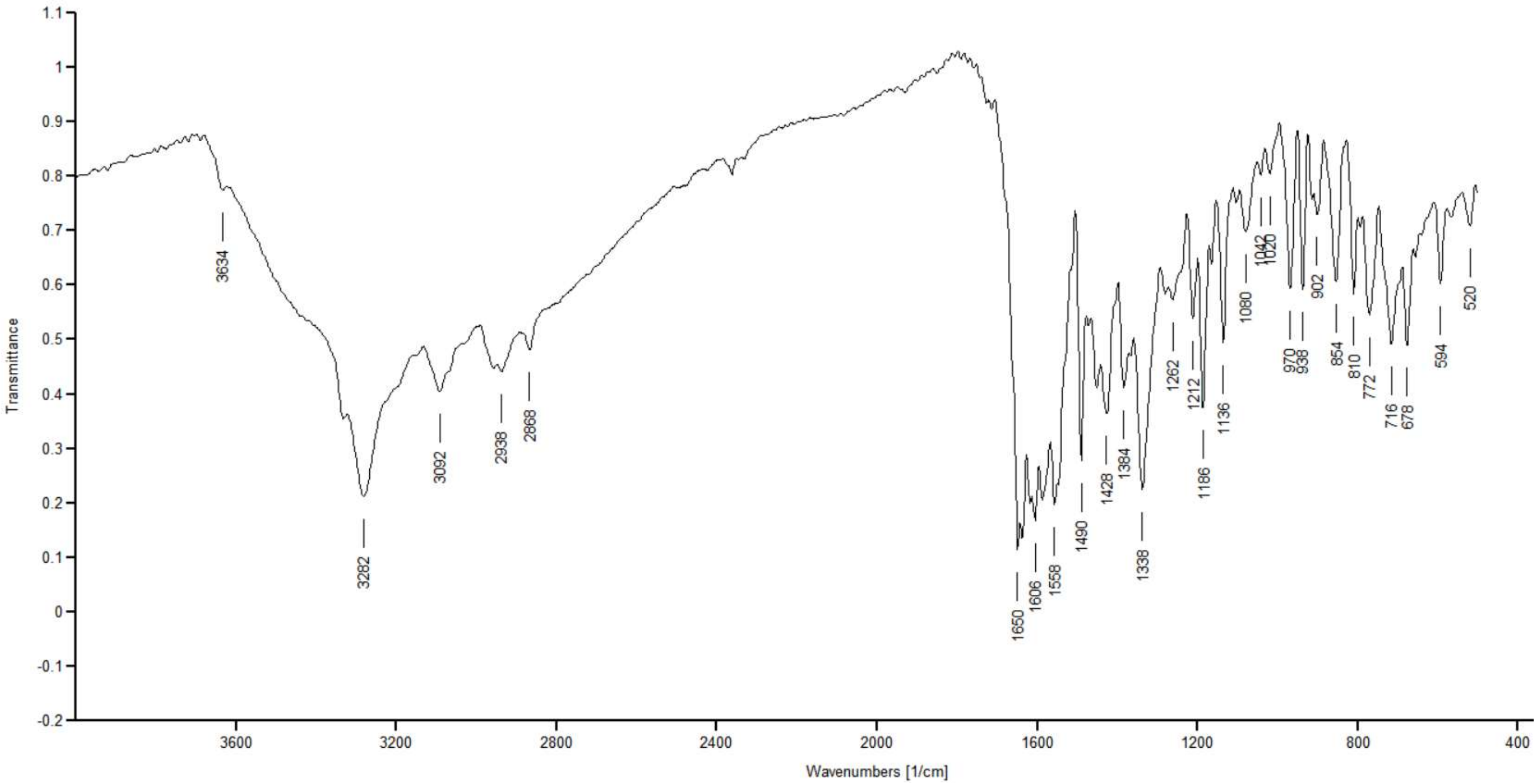

Transmittance
Wavenumbers [1/cm]
3634
3282
3092
2938
2868
1650
1606
1558
1490
1428
1384
1338
1262
1212
1186
1136
1080
1042
1020
970
938
902
854
810
772
716
678
594
520

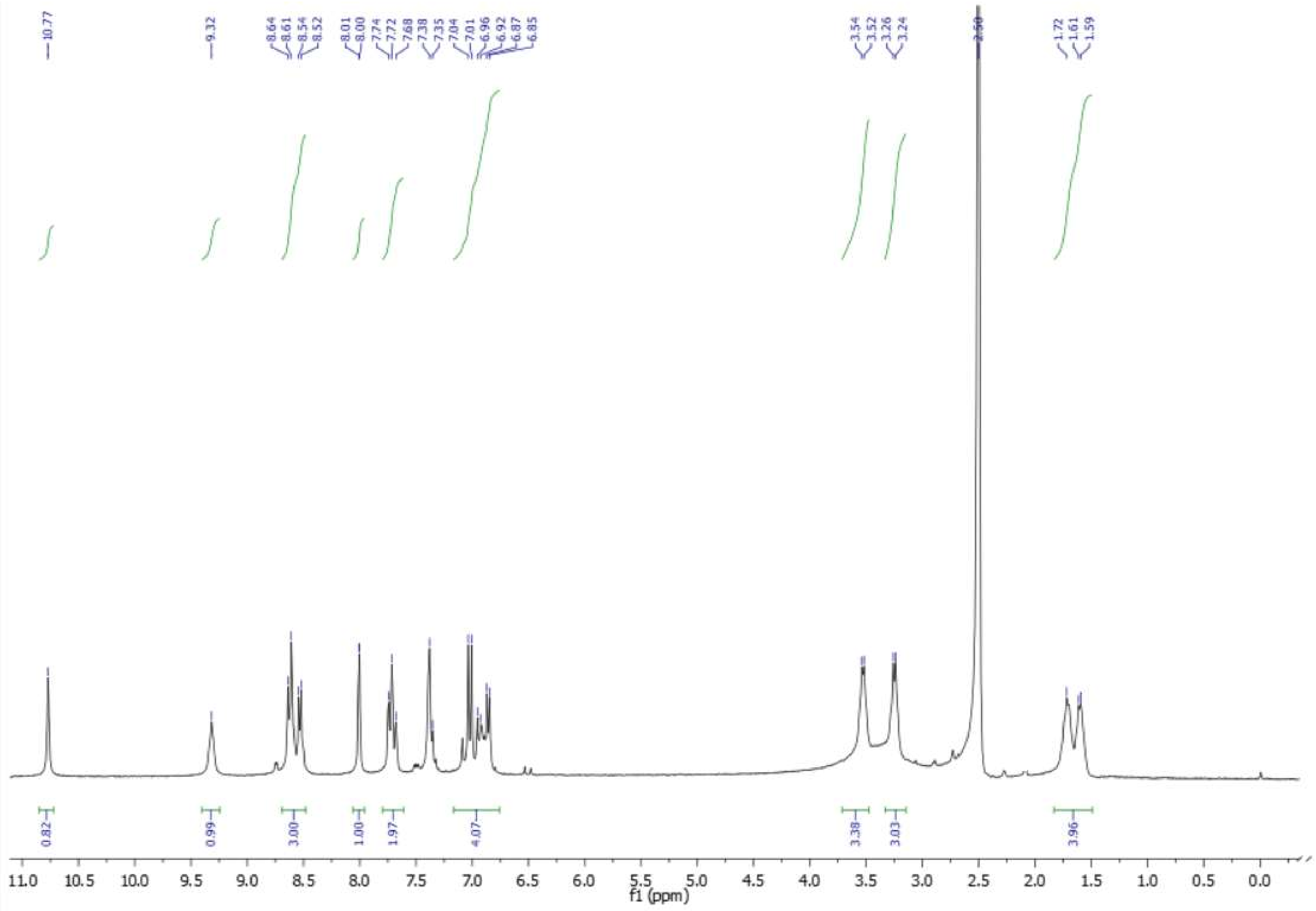

10.77
9.32
8.64
8.61
8.54
8.52
8.01
8.00
7.74
7.72
7.68
7.38
7.35
7.04
7.01
6.96
6.92
6.87
6.85
3.54
3.52
3.26
3.24
2.50
1.72
1.61
1.59
0.82
0.99
3.00
1.00
1.97
4.07
3.38
3.03
3.96
11.0
10.5
10.0
9.5
9.0
8.5
8.0
7.5
7.0
6.5
6.0
5.5
5.0
4.5
4.0
3.5
3.0
2.5
2.0
1.5
1.0
0.5
0.0
f1 (ppm)

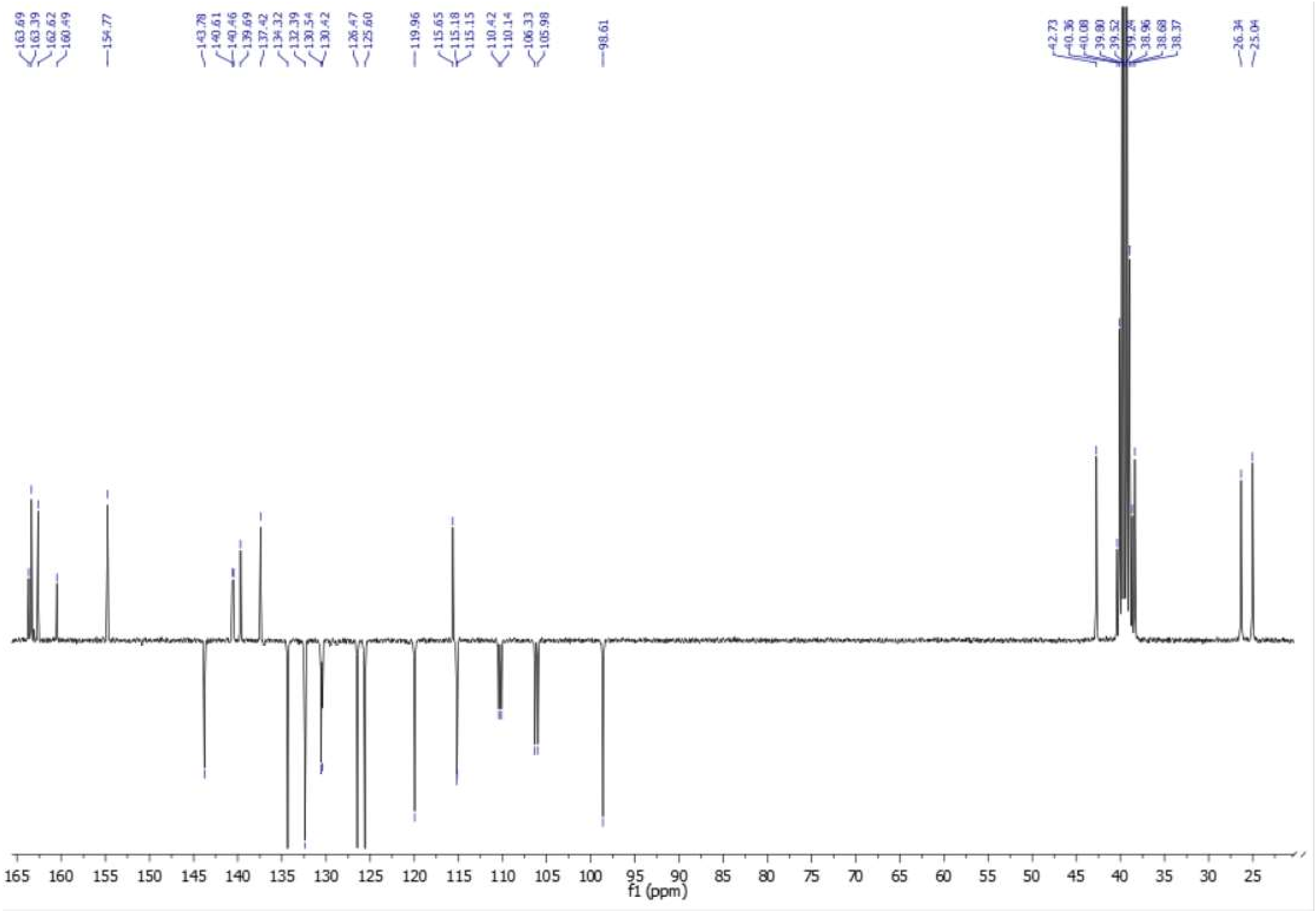
163.69
163.39
162.62
160.49
154.77
143.78
140.61
140.46
139.69
137.42
134.32
132.39
130.54
130.42
126.47
125.60
119.96
115.65
115.18
115.15
110.42
110.14
106.33
105.98
98.61
42.73
40.36
40.08
39.80
39.52
39.24
38.96
38.68
38.37
26.34
25.04
f1 (ppm)

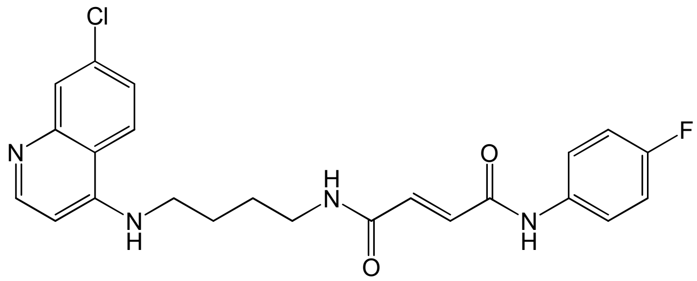
Cl
N
N
H
H
N
O
O
N
H
F

12

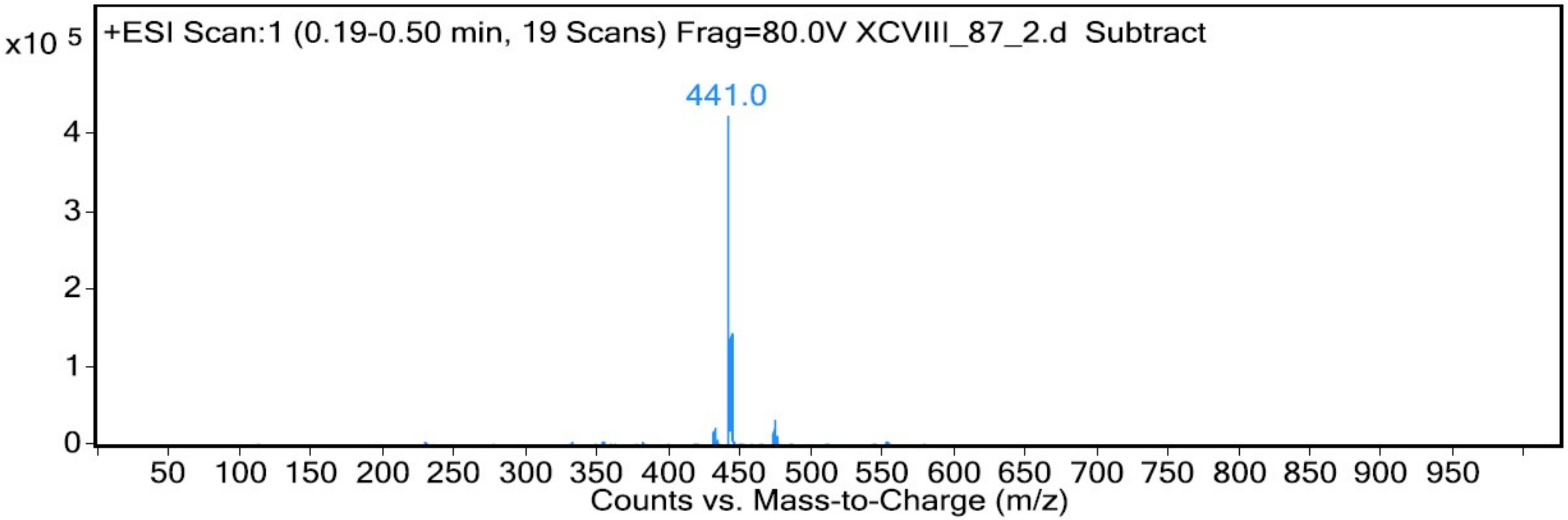
x10 5
+ESI Scan:1 (0.19-0.50 min, 19 Scans) Frag=80.0V XCVIII_87_2.d Subtract
441.0
4
3
2
1
0
50 100 150 200 250 300 350 400 450 500 550 600 650 700 750 800 850 900 950
Counts vs. Mass-to-Charge (m/z)

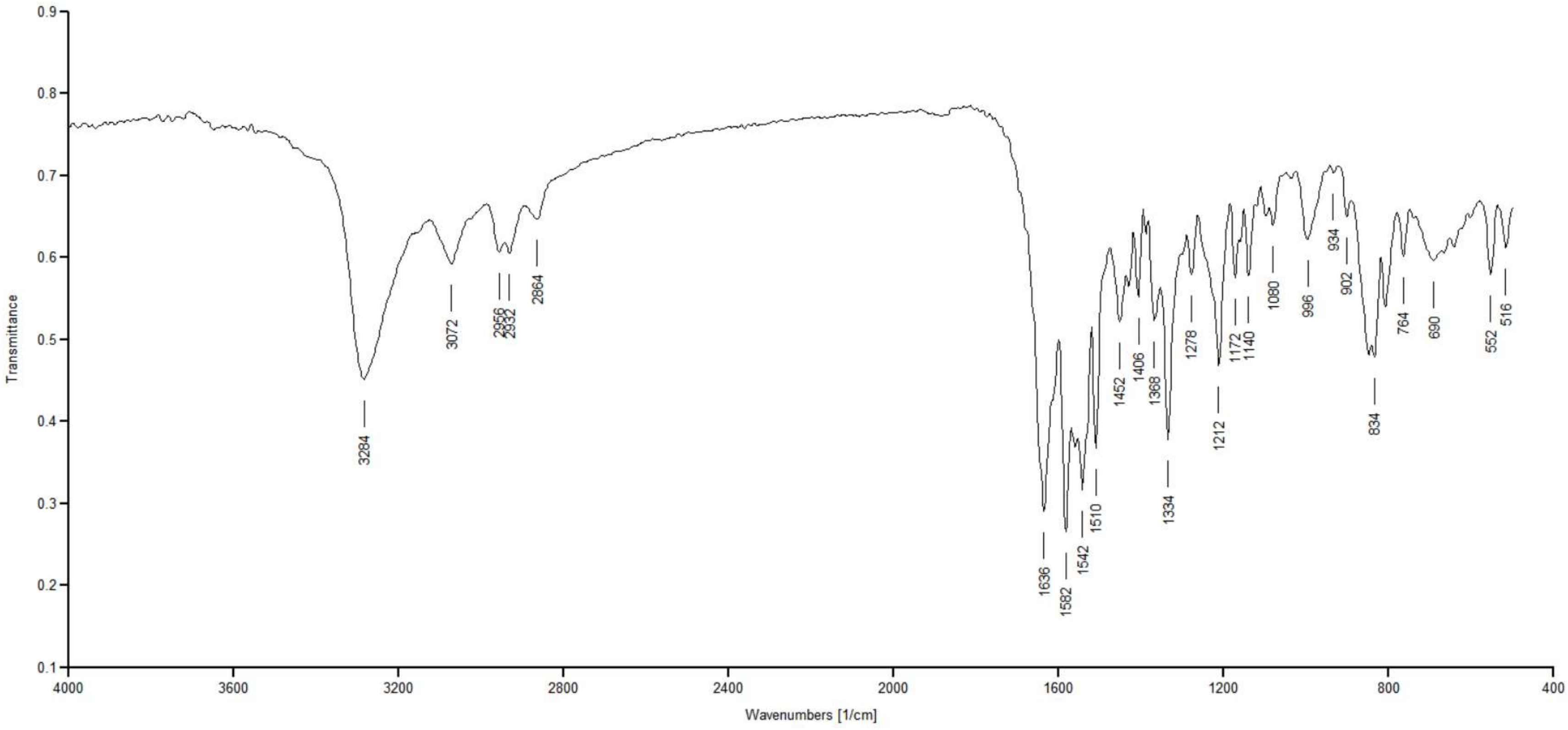
Transmittance
0.9
0.8
0.7
0.6
0.5
0.4
0.3
0.2
0.1
4000
3600
3200
2800
2400
2000
1600
1200
800
400
Wavenumbers [1/cm]
3284
3072
2956
2932
2864
1636
1582
1542
1510
1452
1406
1368
1334
1278
1212
1172
1140
1080
996
934
902
834
764
690
552
516

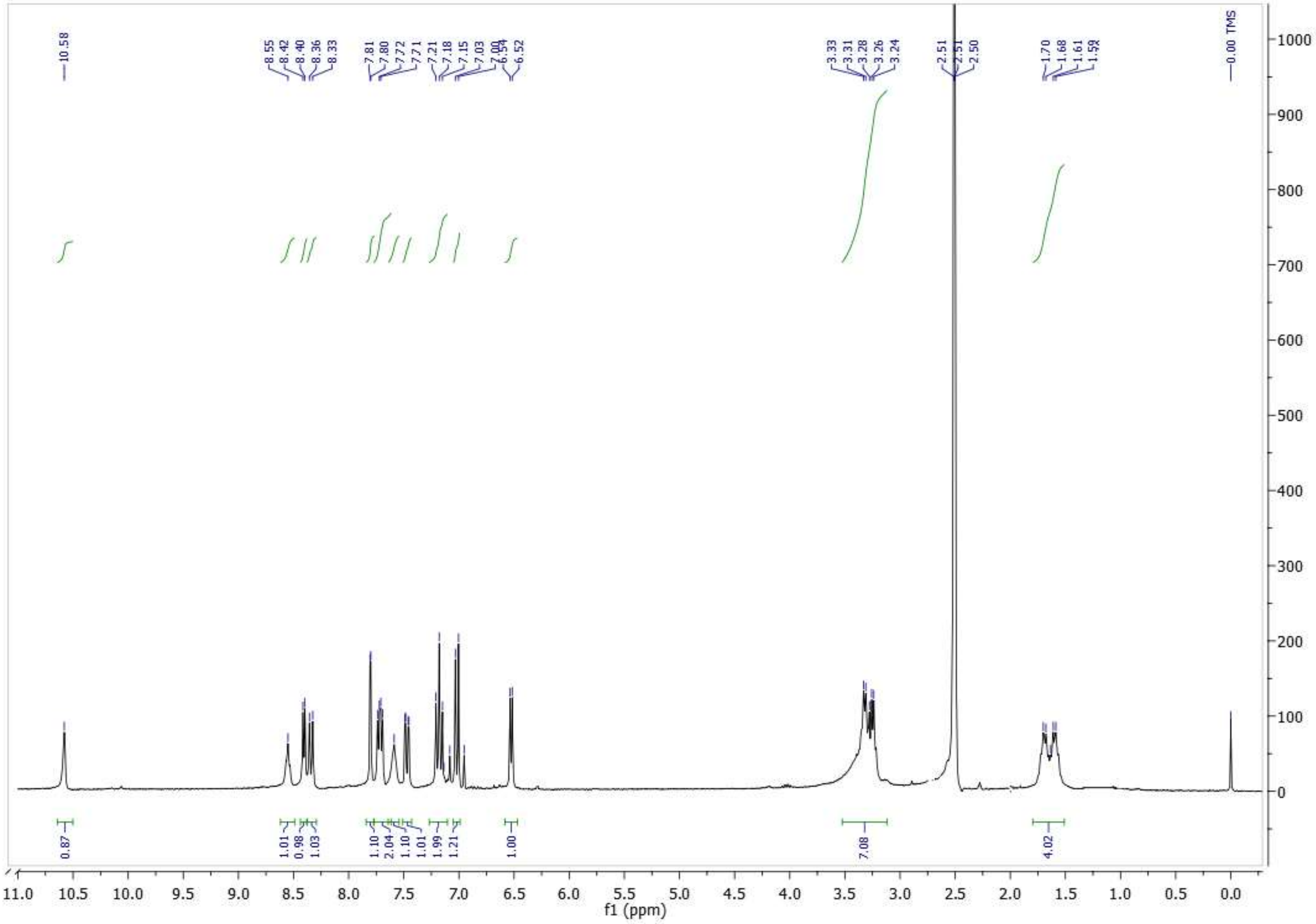

10.58
8.55
8.42
8.40
8.36
8.33
7.81
7.80
7.72
7.71
7.21
7.18
7.15
7.03
7.00
6.54
6.52
3.33
3.31
3.28
3.26
3.24
2.51
2.51
2.50
1.70
1.68
1.61
1.59
0.00 TMS
0.87
1.01
0.98
1.03
1.10
2.04
1.10
1.01
1.99
1.21
1.00
7.08
4.02
f1 (ppm)

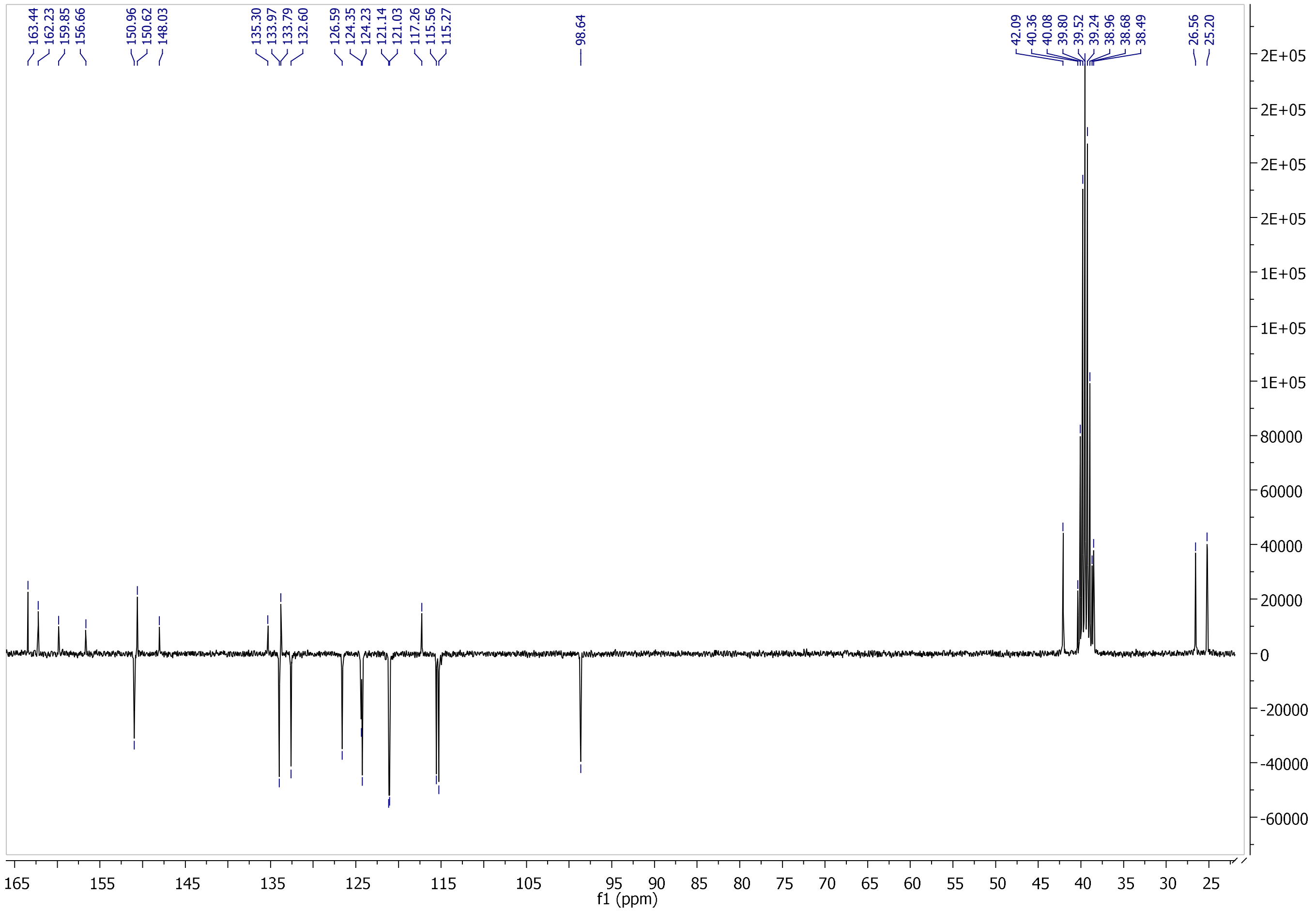
163.44
162.23
159.85
156.66
150.96
150.62
148.03
135.30
133.97
133.79
132.60
126.59
124.35
124.23
121.14
121.03
117.26
115.56
115.27
98.64
42.09
40.36
40.08
39.80
39.52
39.24
38.96
38.68
38.49
26.56
25.20
f1 (ppm)

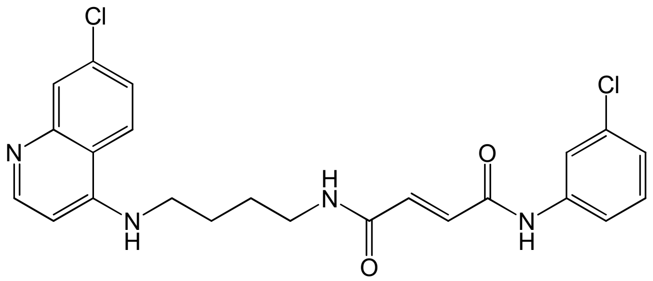
Cl
N
N
H
H
N
O
O
N
H
Cl

13

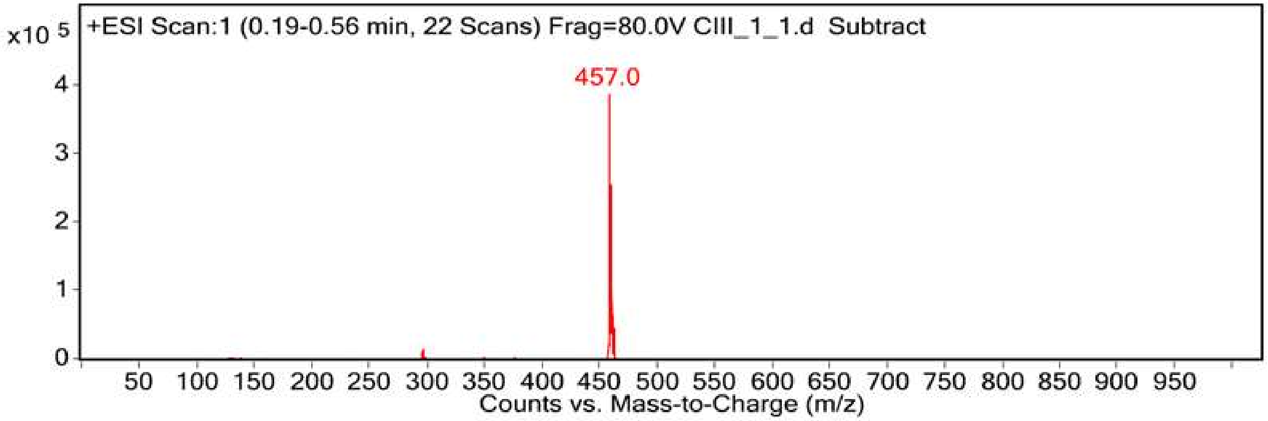
x10 5
+ESI Scan:1 (0.19-0.56 min, 22 Scans) Frag=80.0V CIII_1_1.d Subtract
457.0
4
3
2
1
0
50 100 150 200 250 300 350 400 450 500 550 600 650 700 750 800 850 900 950
Counts vs. Mass-to-Charge (m/z)

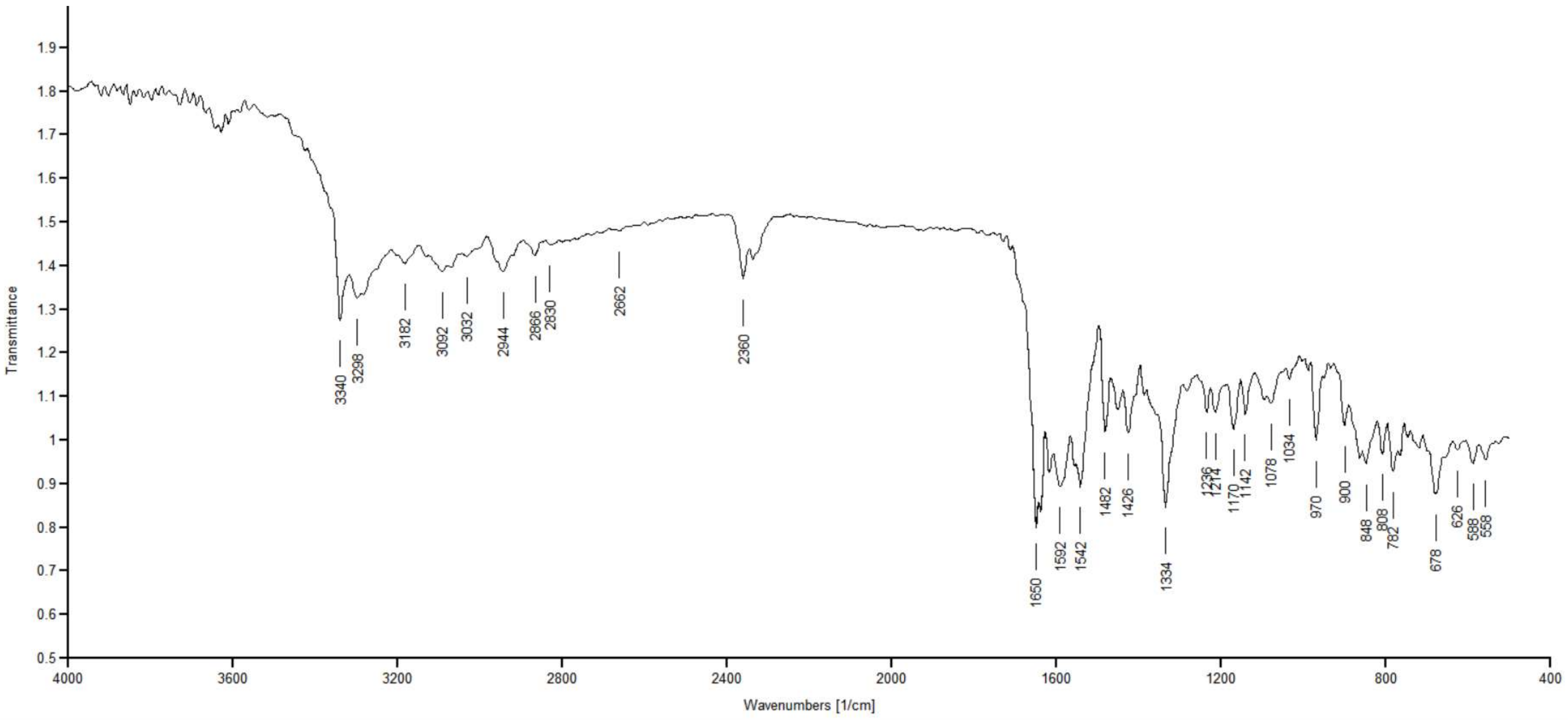

Transmittance
Wavenumbers [1/cm]
1.9
1.8
1.7
1.6
1.5
1.4
1.3
1.2
1.1
1
0.9
0.8
0.7
0.6
0.5
4000
3600
3200
2800
2400
2000
1600
1200
800
400
3340
3298
3182
3092
3032
2944
2866
2830
2662
2360
1650
1592
1542
1482
1426
1334
1236
1214
1170
1142
1078
1034
970
900
848
808
782
678
626
588
558

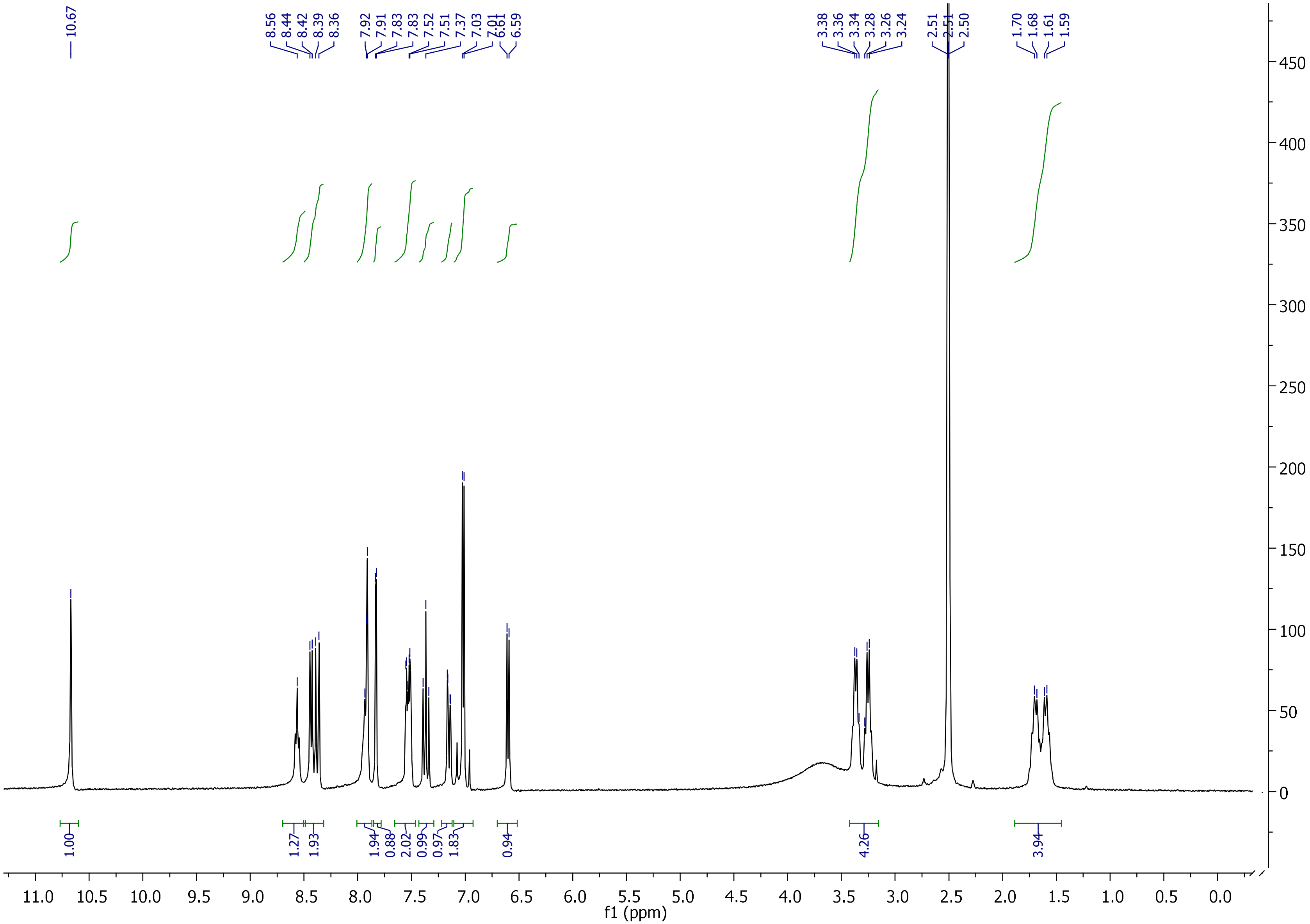
10.67
8.56
8.44
8.42
8.39
8.36
7.92
7.91
7.83
7.83
7.52
7.51
7.37
7.03
7.01
6.61
6.59
3.38
3.36
3.34
3.28
3.26
3.24
2.51
2.51
2.50
1.70
1.68
1.61
1.59
1.00
1.27
1.93
1.94
0.88
2.02
0.99
0.97
1.83
0.94
4.26
3.94
f1 (ppm)

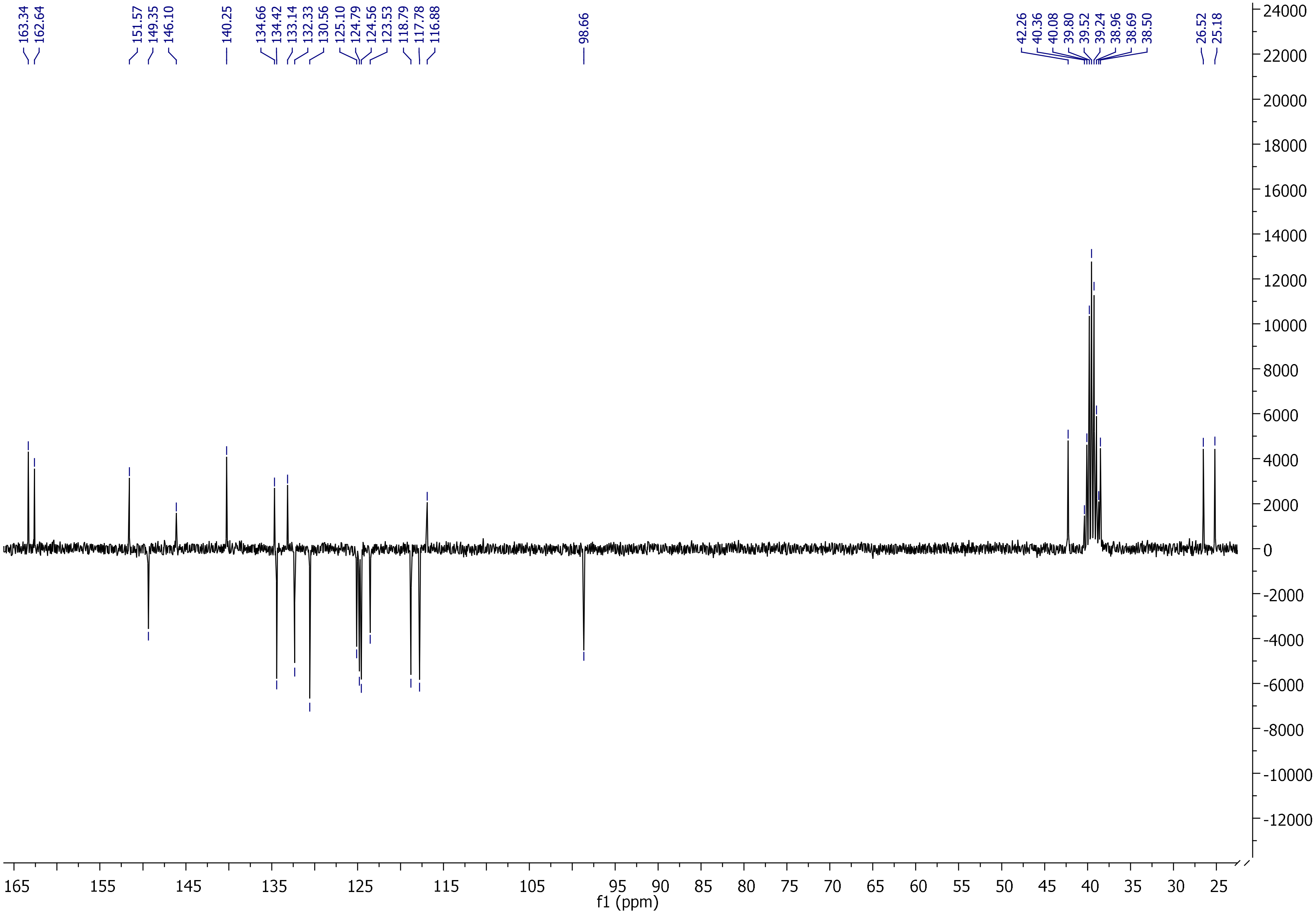
163.34
162.64
151.57
149.35
146.10
140.25
134.66
134.42
133.14
132.33
130.56
125.10
124.79
124.56
123.53
118.79
117.78
116.88
98.66
42.26
40.36
40.08
39.80
39.52
39.24
38.96
38.69
38.50
26.52
25.18
f1 (ppm)

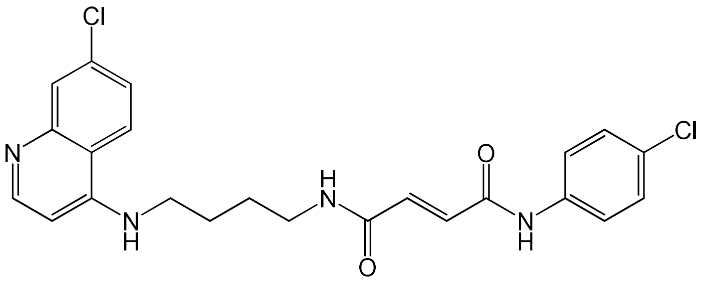
Cl
N
N
H
H
N
O
O
N
H
Cl

14

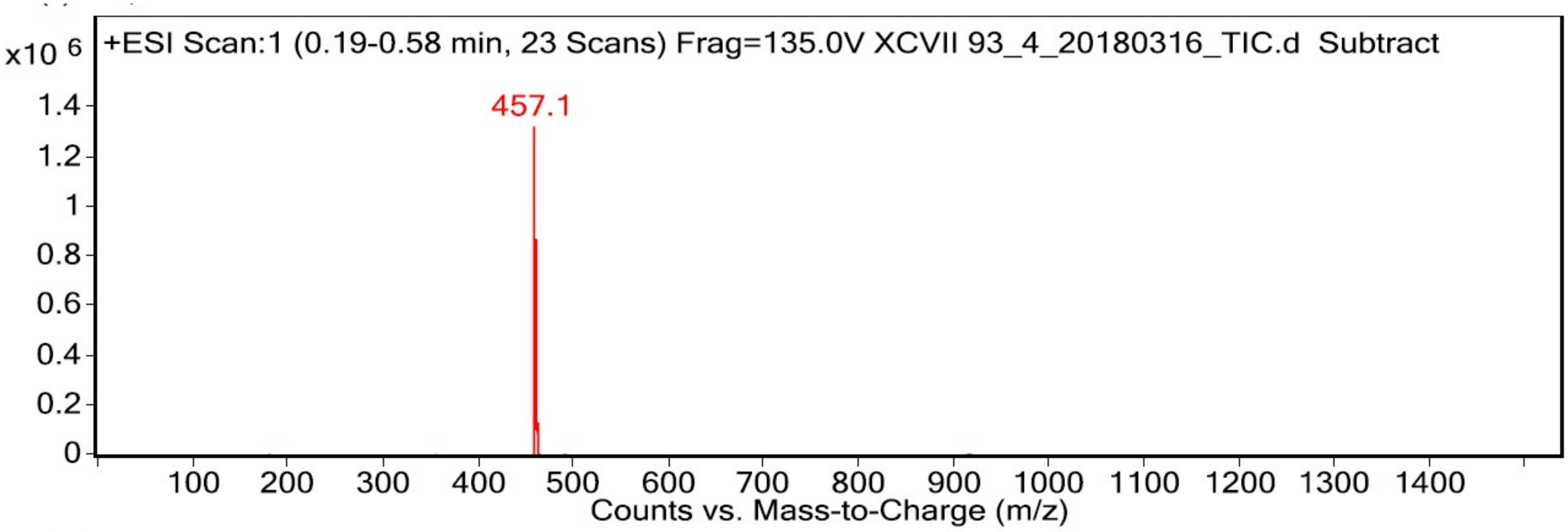
x10 6
+ESI Scan:1 (0.19-0.58 min, 23 Scans) Frag=135.0V XCVII 93_4_20180316_TIC.d Subtract
1.4
1.2
1
0.8
0.6
0.4
0.2
0
457.1
100 200 300 400 500 600 700 800 900 1000 1100 1200 1300 1400
Counts vs. Mass-to-Charge (m/z)

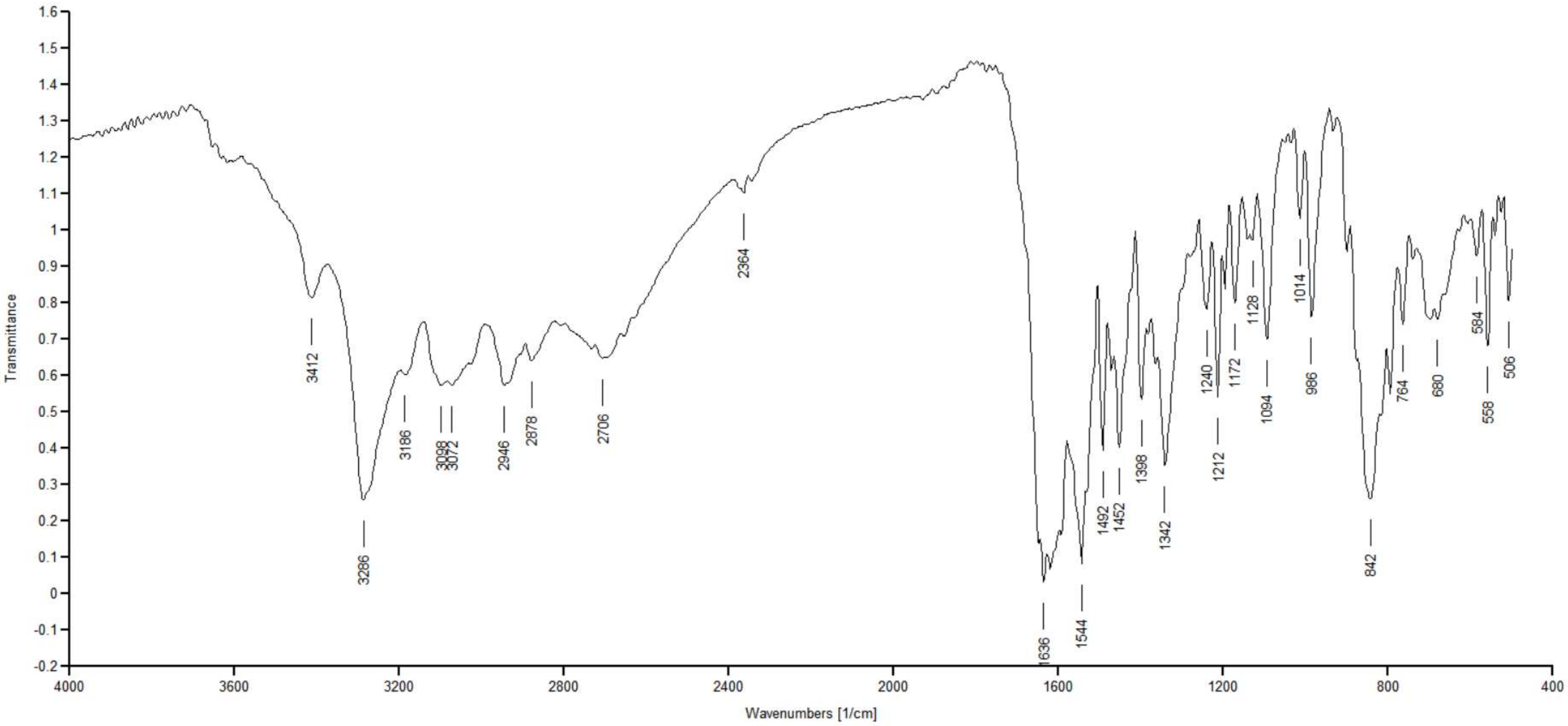
Transmittance
Wavenumbers [1/cm]
3412
3286
3186
3098
3072
2946
2878
2706
2364
1636
1544
1492
1452
1398
1342
1240
1212
1172
1128
1094
1014
986
842
764
680
584
558
506

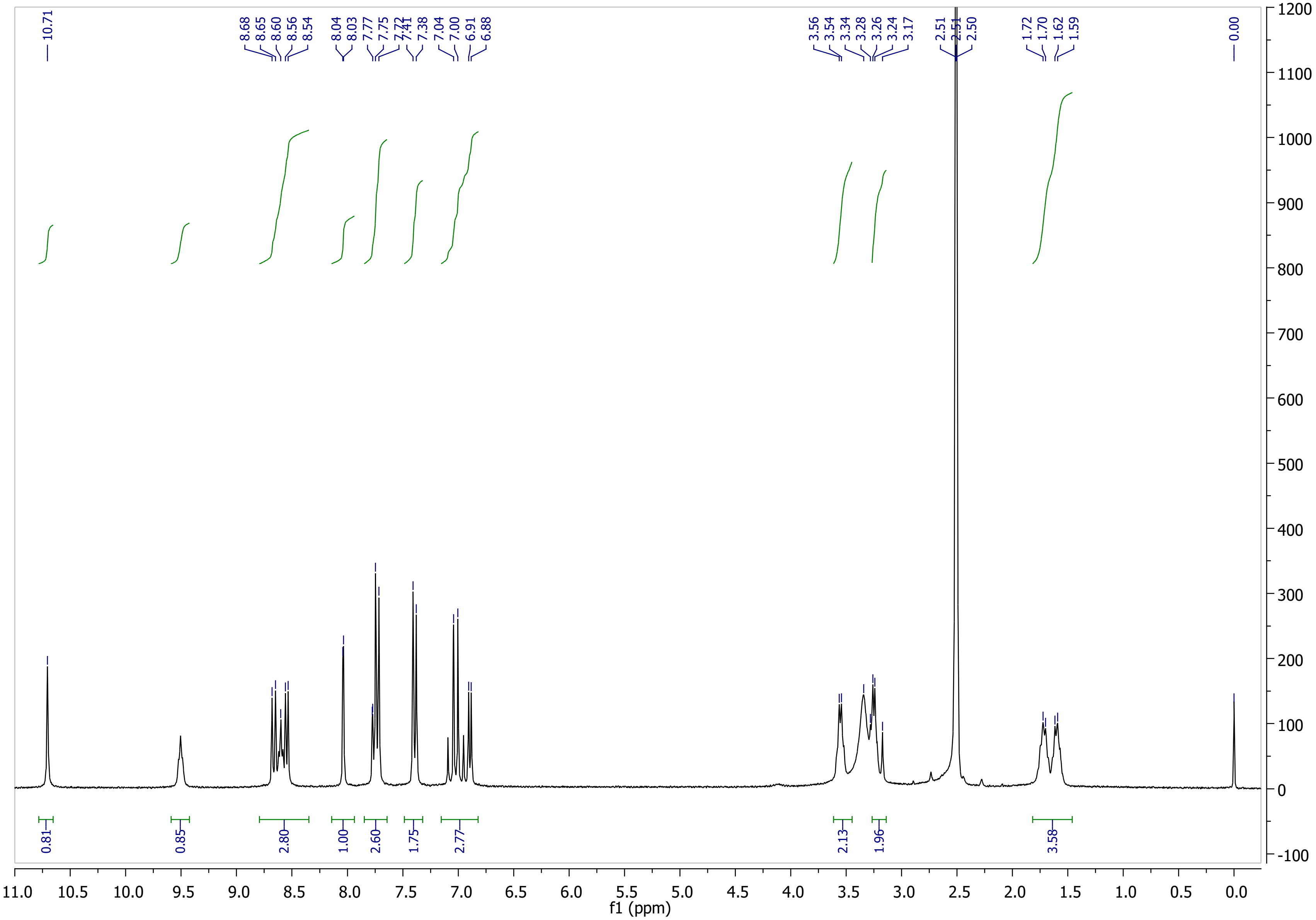
10.71
8.68
8.65
8.60
8.56
8.54
8.04
8.03
7.77
7.75
7.72
7.41
7.38
7.04
7.00
6.91
6.88
3.56
3.54
3.34
3.28
3.26
3.24
3.17
2.51
2.51
2.50
1.72
1.70
1.62
1.59
0.00
0.81
0.85
2.80
1.00
2.60
1.75
2.77
2.13
1.96
3.58
11.0 10.5 10.0 9.5 9.0 8.5 8.0 7.5 7.0 6.5 6.0 5.5 5.0 4.5 4.0 3.5 3.0 2.5 2.0 1.5 1.0 0.5 0.0
f1 (ppm)
1200 1100 1000 900 800 700 600 500 400 300 200 100 0 -100

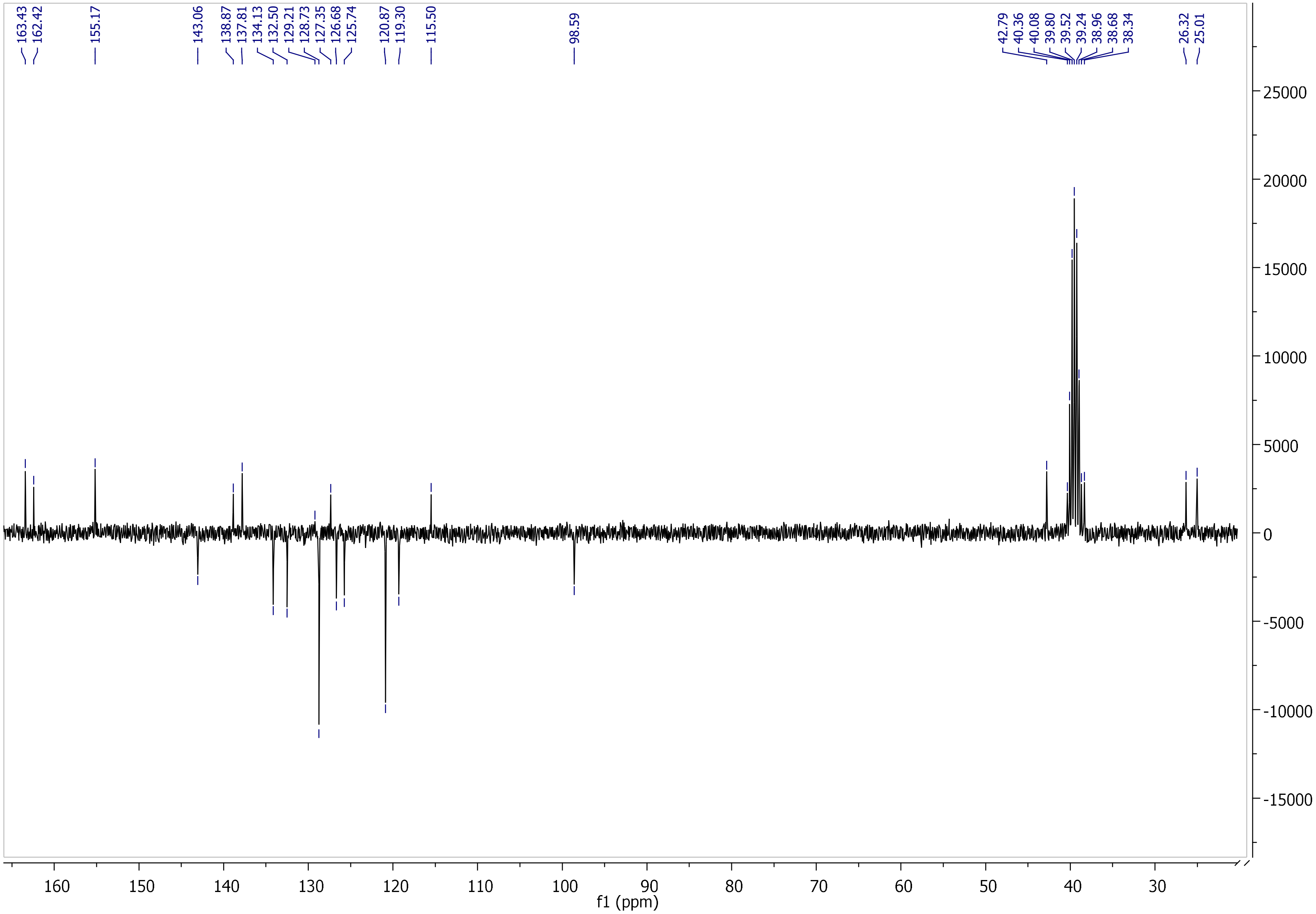
163.43
162.42
155.17
143.06
138.87
137.81
134.13
132.50
129.21
128.73
127.35
126.68
125.74
120.87
119.30
115.50
98.59
42.79
40.36
40.08
39.80
39.52
39.24
38.96
38.68
38.34
26.32
25.01
25000
20000
15000
10000
5000
0
-5000
-10000
-15000
160
150
140
130
120
110
100
90
80
70
60
50
40
30
f1 (ppm)

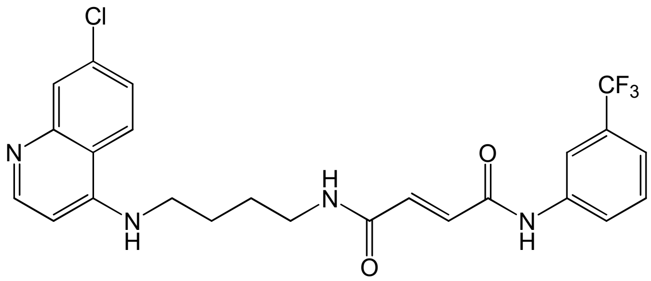
Cl
N
N
H
H
N
O
O
N
H
CF3

15

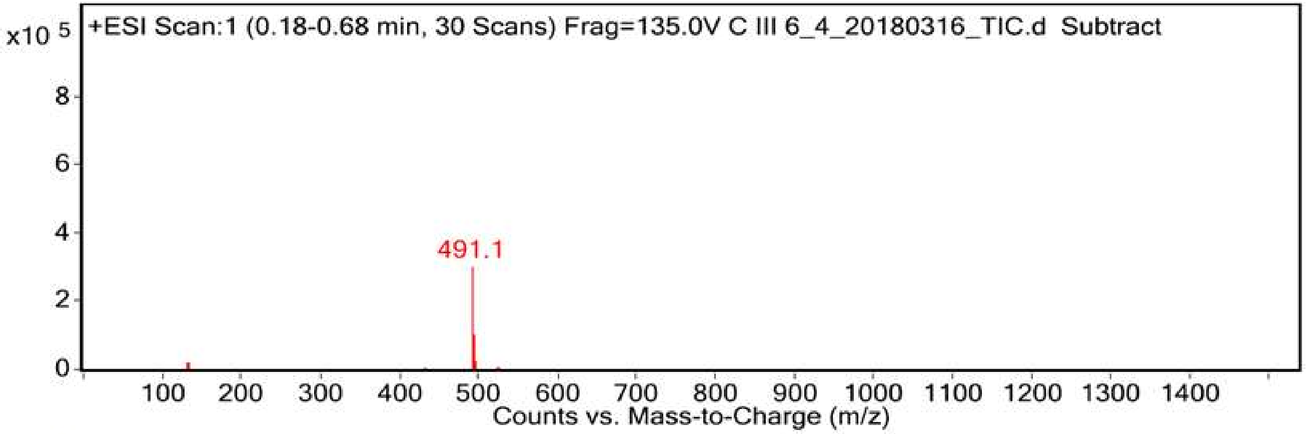
+ESI Scan:1 (0.18-0.68 min, 30 Scans) Frag=135.0V C III 6_4_20180316_TIC.d Subtract
x10 5
8
6
4
2
0
491.1
100 200 300 400 500 600 700 800 900 1000 1100 1200 1300 1400
Counts vs. Mass-to-Charge (m/z)

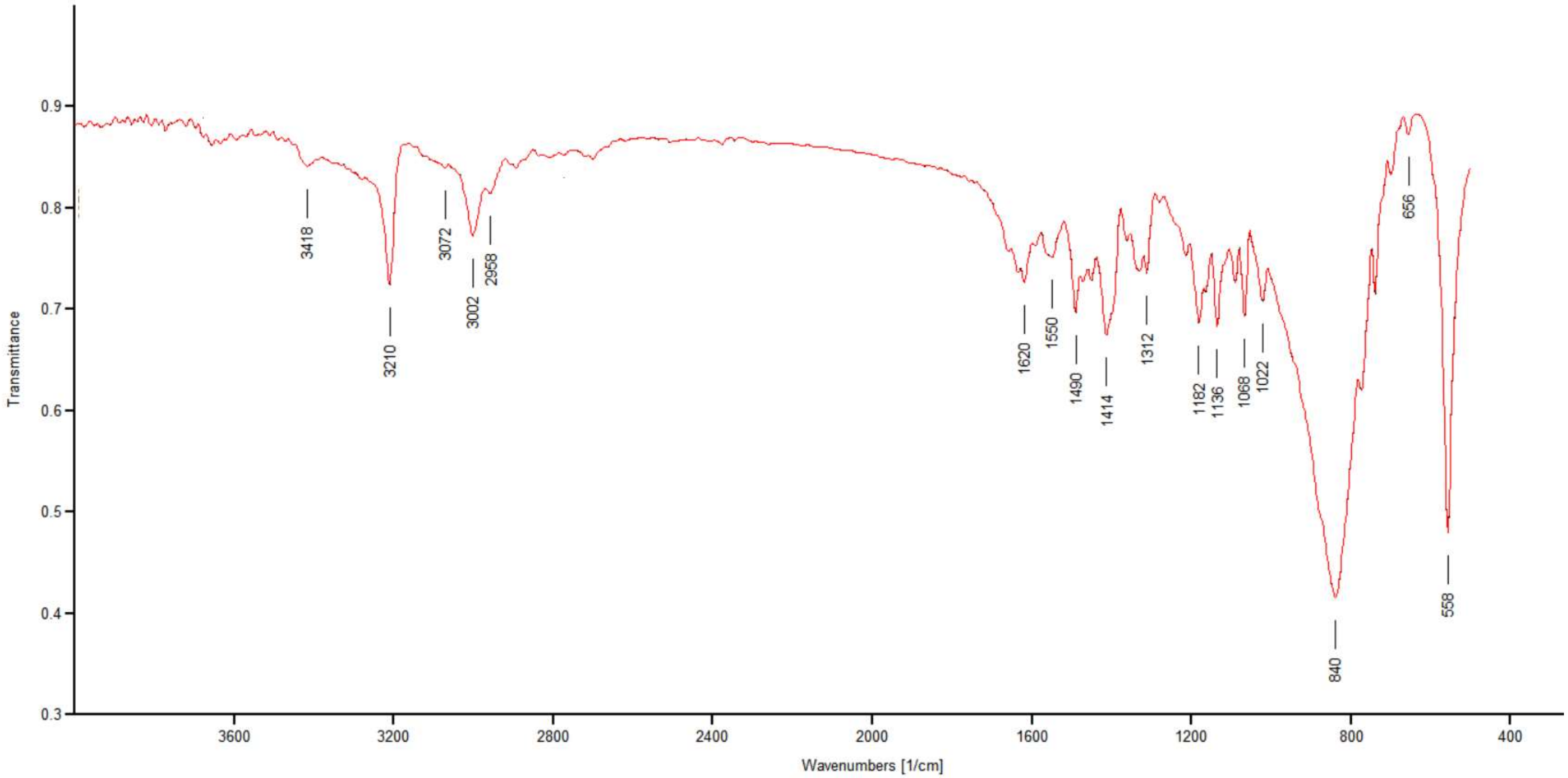

Transmittance
0.9
0.8
0.7
0.6
0.5
0.4
0.3
3600
3200
2800
2400
2000
1600
1200
800
400
Wavenumbers [1/cm]
3418
3210
3072
3002
2958
1620
1550
1490
1414
1312
1182
1136
1068
1022
840
656
558

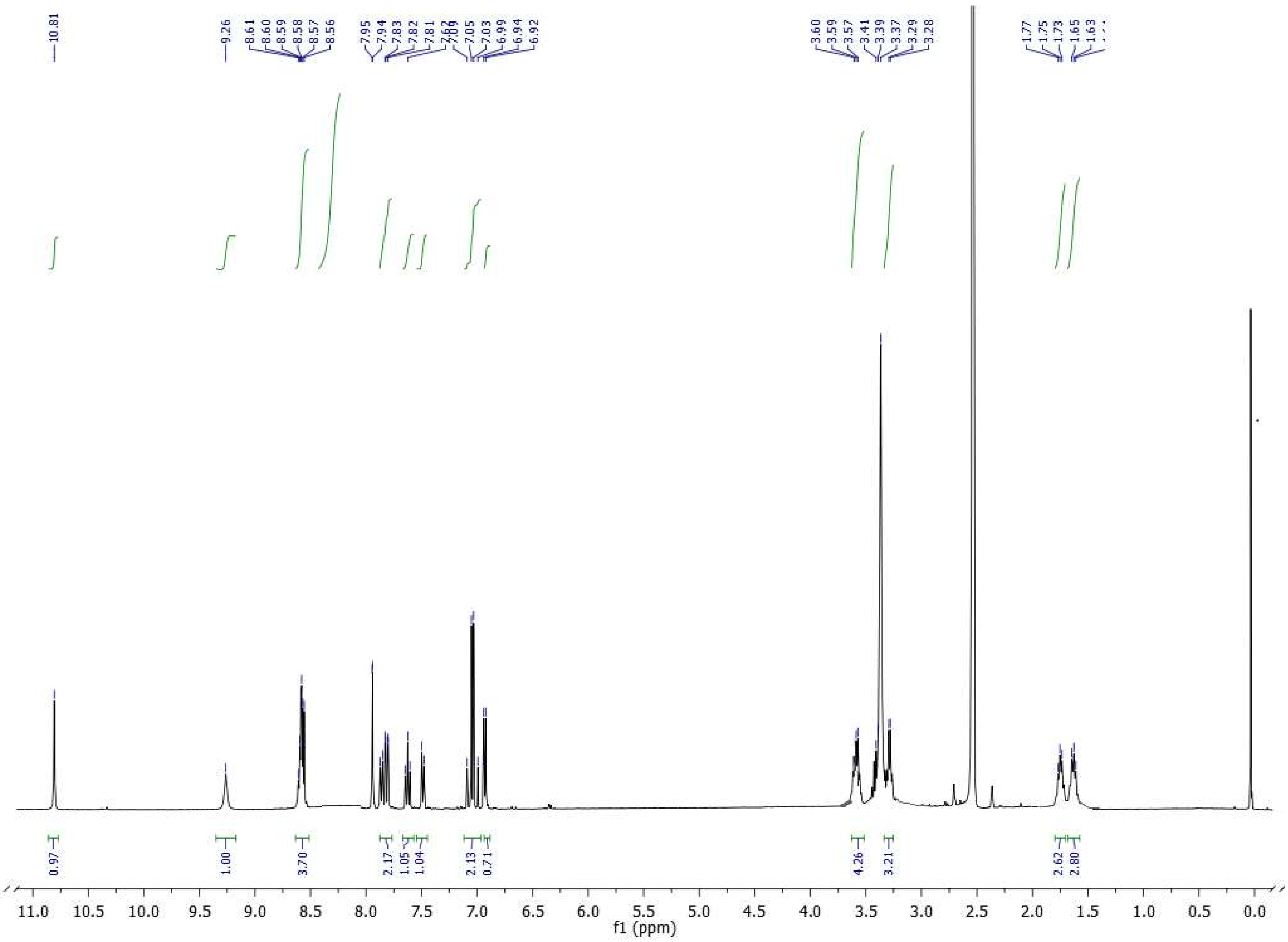
10.81
9.26
8.61 8.60 8.59 8.58 8.57 8.56
7.95 7.94 7.83 7.82 7.81 7.62 7.09 7.05 7.03 6.99 6.94 6.92
3.60 3.59 3.57 3.41 3.39 3.37 3.29 3.28
1.77 1.75 1.73 1.65 1.63
0.97 1.00 3.70 2.17 1.05 1.04 2.13 0.71 4.26 3.21 2.62 2.80
11.0 10.5 10.0 9.5 9.0 8.5 8.0 7.5 7.0 6.5 6.0 5.5 5.0 4.5 4.0 3.5 3.0 2.5 2.0 1.5 1.0 0.5 0.0
f1 (ppm)

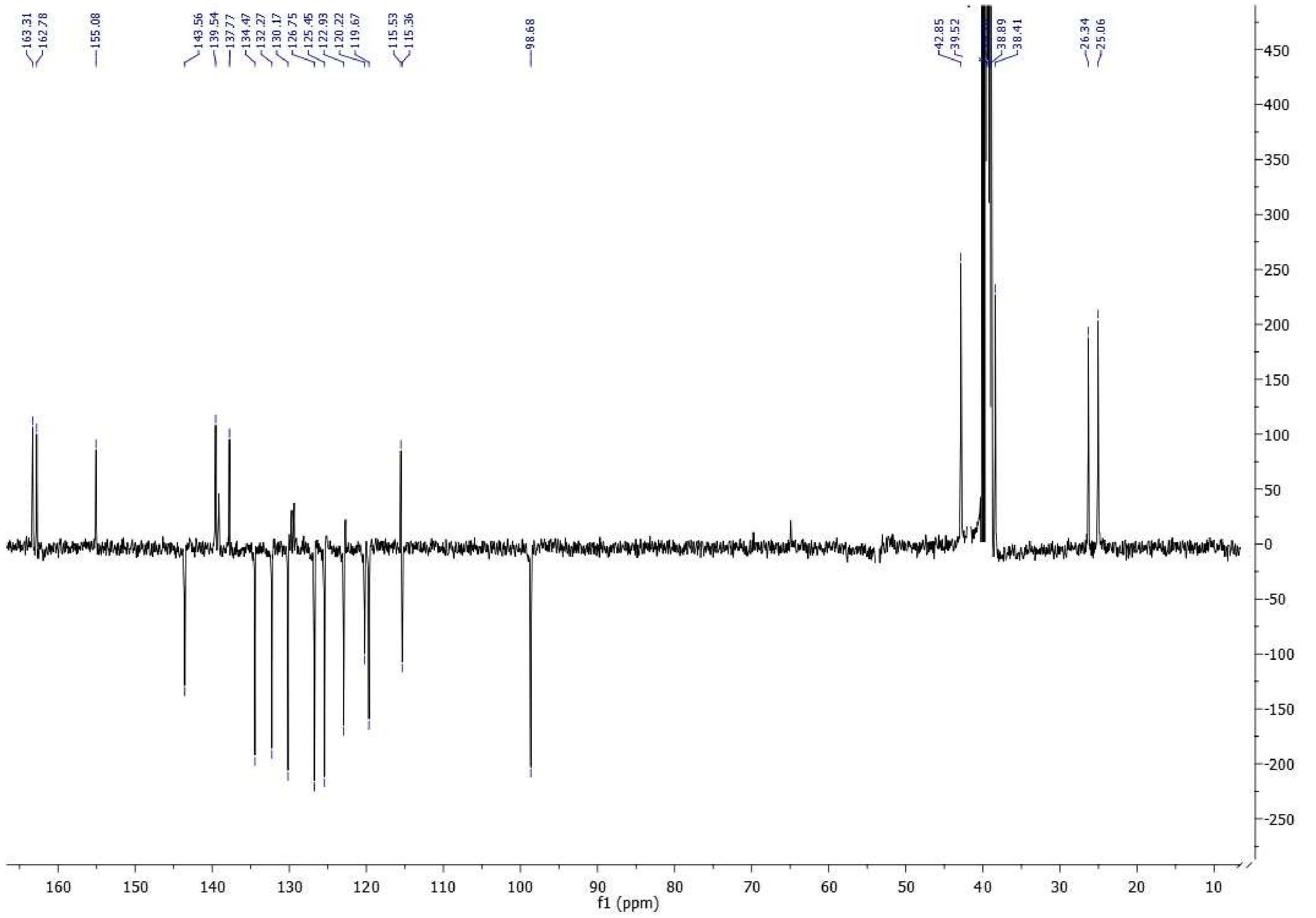

163.31
162.78
155.08
143.56
139.54
137.77
134.47
132.27
130.17
126.75
125.45
122.93
120.22
119.67
115.53
115.36
98.68
42.85
39.52
38.89
38.41
26.34
25.06
f1 (ppm)

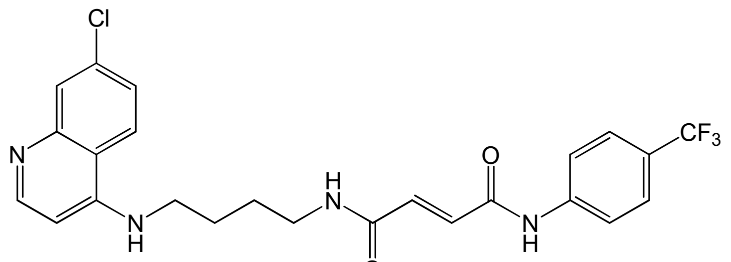
Cl
N
N
H
H
N
O
O
N
H
CF_3

16

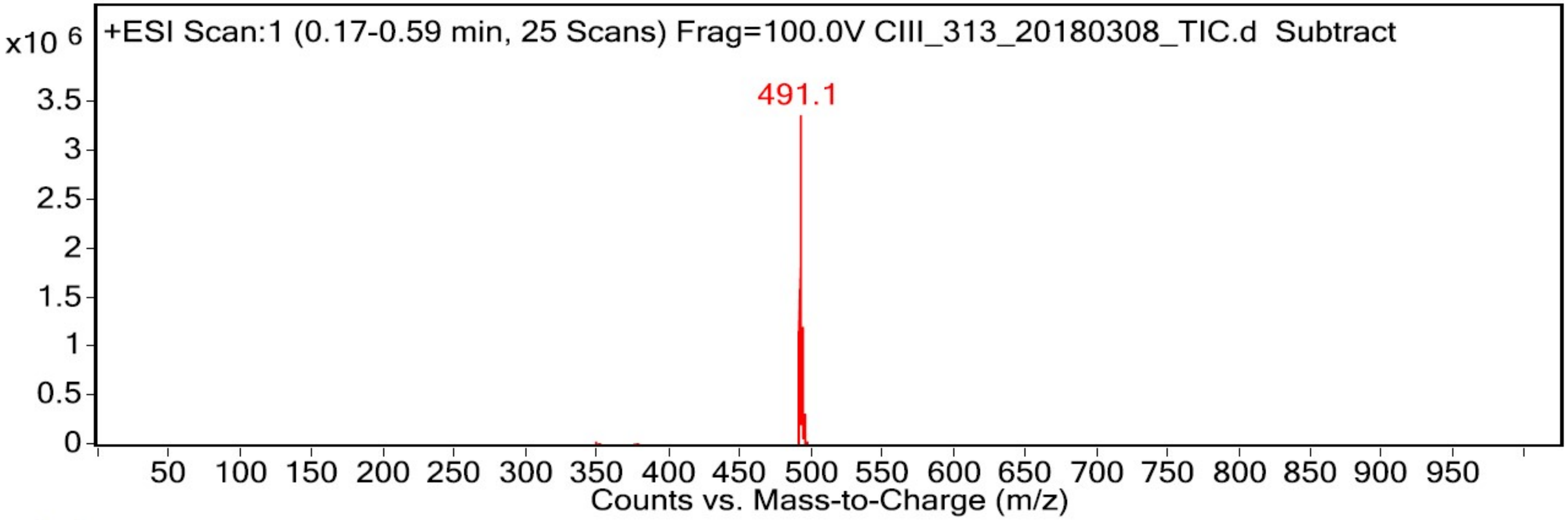
x10 6
+ESI Scan:1 (0.17-0.59 min, 25 Scans) Frag=100.0V CIII_313_20180308_TIC.d Subtract
491.1
3.5
3
2.5
2
1.5
1
0.5
0
50 100 150 200 250 300 350 400 450 500 550 600 650 700 750 800 850 900 950
Counts vs. Mass-to-Charge (m/z)

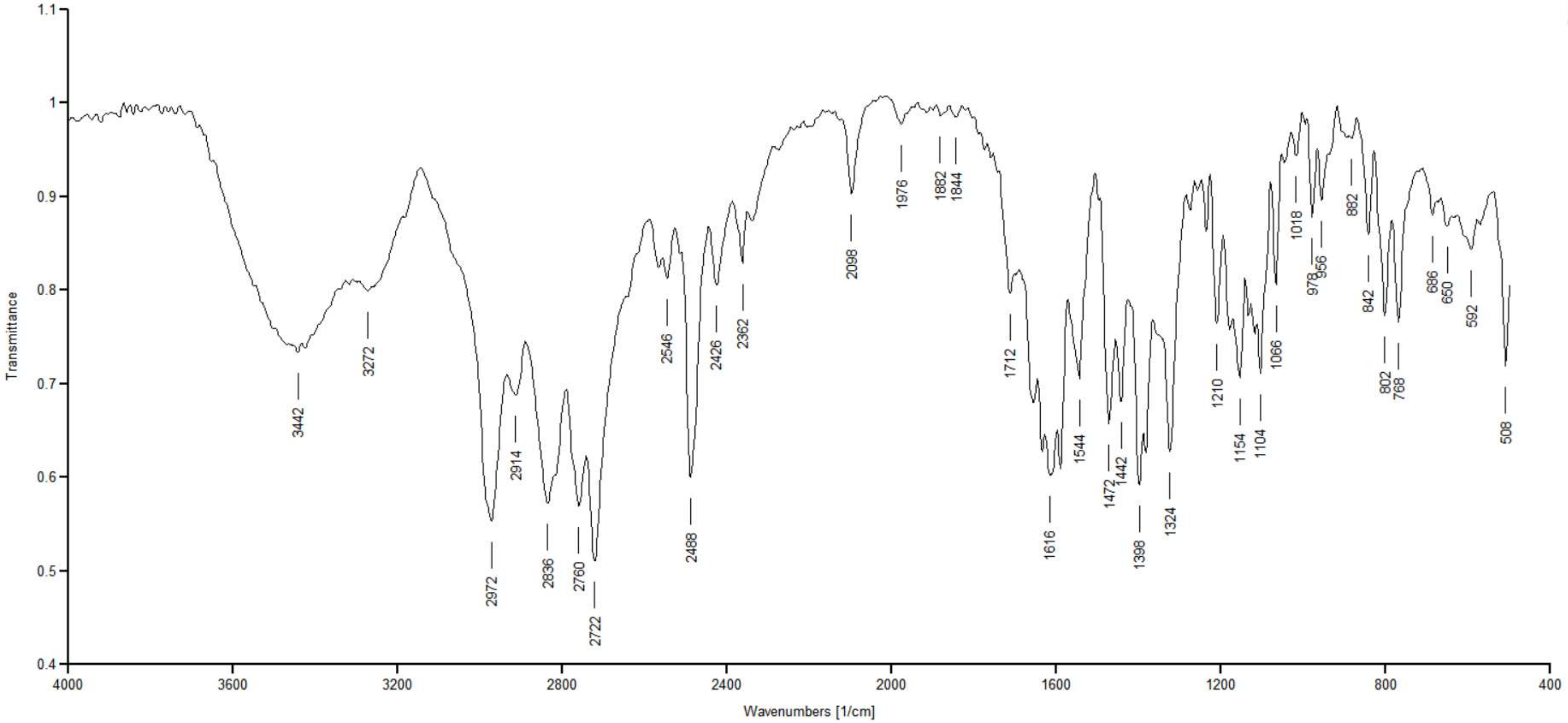
Transmittance
Wavenumbers [1/cm]
1.1
1
0.9
0.8
0.7
0.6
0.5
0.4
4000
3600
3200
2800
2400
2000
1600
1200
800
400
3442
3272
2972
2914
2836
2760
2722
2546
2488
2426
2362
2098
1976
1882
1844
1712
1616
1544
1472
1442
1398
1324
1210
1154
1104
1066
1018
978
956
882
842
802
768
686
650
592
508

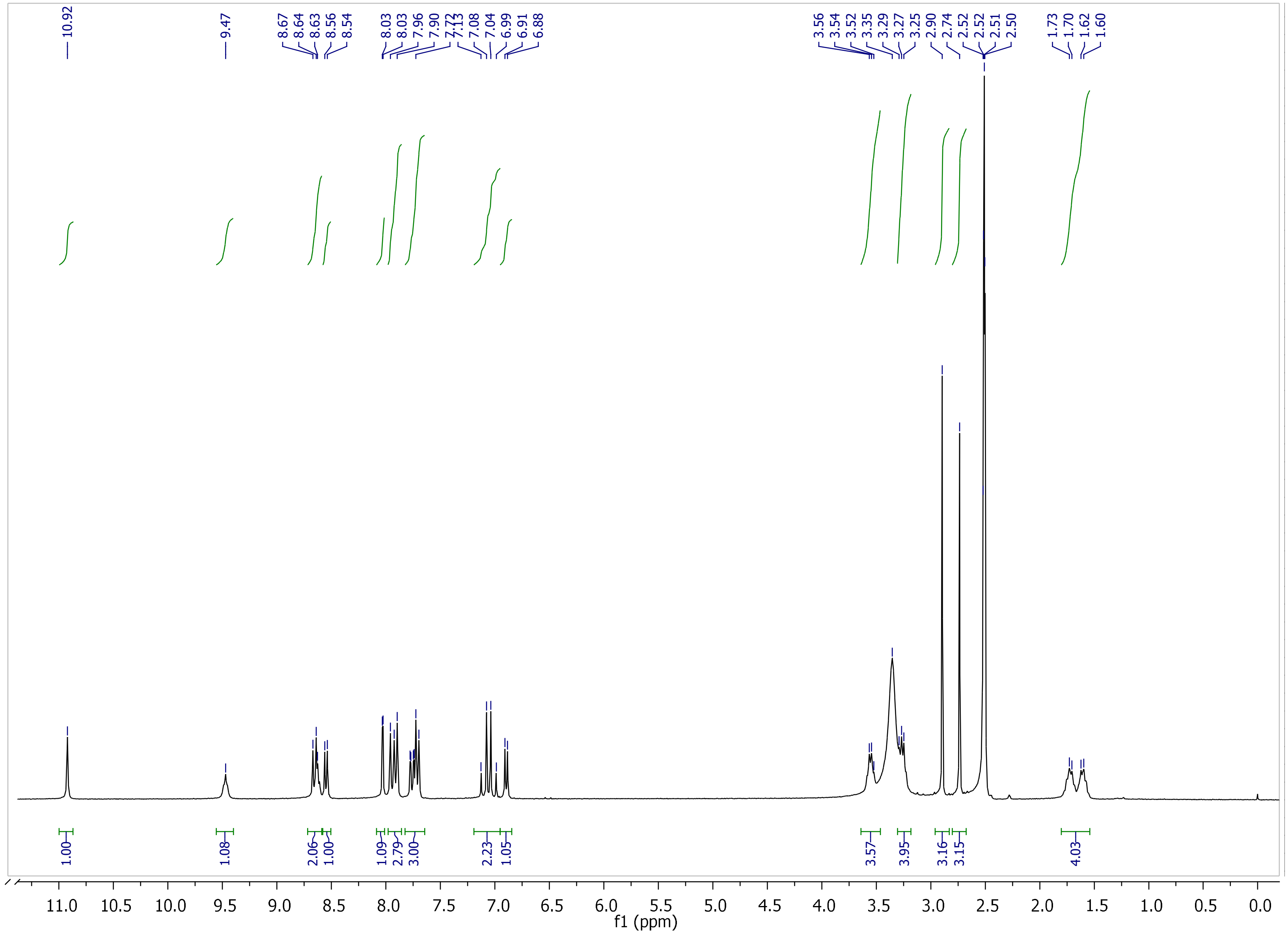
10.92
9.47
8.67 8.64 8.63 8.56 8.54
8.03 8.03 7.96 7.90 7.72 7.13 7.08 7.04 6.99 6.91 6.88
3.56 3.54 3.52 3.35 3.29 3.27 3.25 2.90 2.74 2.52 2.52 2.51 2.50
1.73 1.70 1.62 1.60
1.00 1.08 2.06 1.00 1.09 2.79 3.00 2.23 1.05 3.57 3.95 3.16 3.15 4.03
f1 (ppm)

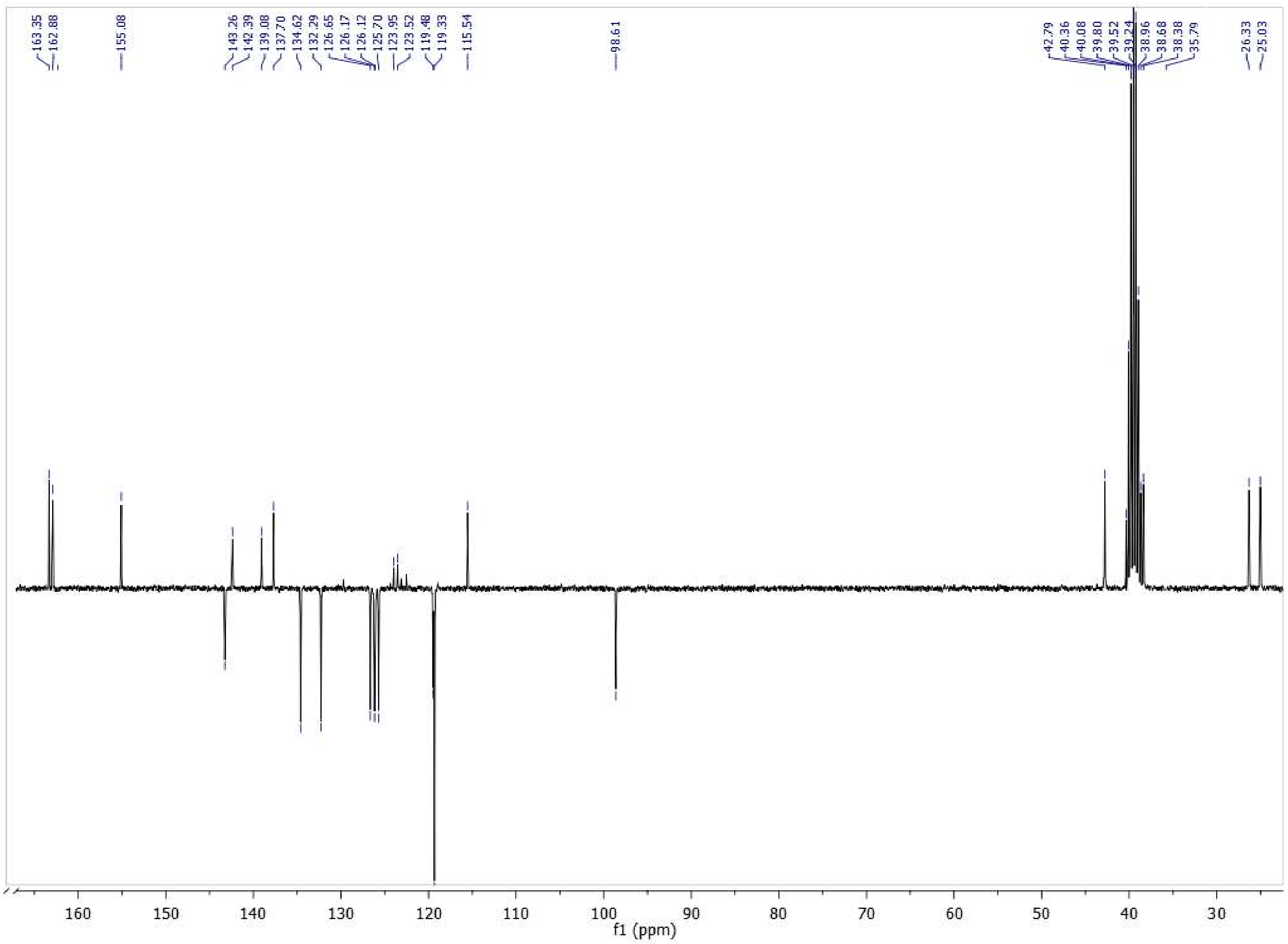
163.35
162.88
155.08
143.26
142.39
139.08
137.70
134.62
132.29
126.65
126.17
126.12
125.70
123.95
123.52
119.48
119.33
115.54
98.61
42.79
40.36
40.08
39.80
39.52
39.24
38.96
38.68
38.38
35.79
26.33
25.03
160
150
140
130
120
110
100
90
80
70
60
50
40
30
f1 (ppm)
